# Supplementary material for: Reduced health services at under-electrified primary healthcare facilities: Evidence from India
Source: PLoS One. 2021 Jun 4;16(6):e0252705. doi: 10.1371/journal.pone.0252705 (PMC8177862; doi:10.1371/journal.pone.0252705)
Supplement: S1 Appendix — (DOCX) [file pone.0252705.s001.docx]

**S1 Appendix**

**Fig A1: Proportion of PHCs and SCs in India without electricity supply.**

**Table A1: Coefficients for full model specification (Model 3) for all three outcome variables.**

|  | | | |
| --- | --- | --- | --- |
|  | Dependent variable: | | |
|  |  | | |
|  | **Deliveries** | **In-Patient** | **Out-Patient** |
|  | zero-inflated | zero-inflated | negative |
|  | count data | count data | binomial |
|  | (1) | (2) | (3) |
|  | | | |
| **Electricity: Irregular Electricity** | 0.97 | 1.05 | 0.94 |
| **Electricity: No Electricity** | 0.36*** | 1.52* | 0.62*** |
| **Generator** | 1.03 | 1.22** | 1.25*** |
|  |  |  |  |
| Urban | 0.78*** | 0.81** | 0.95 |
| Population1000 | 1.05*** | 1.02*** | 1.02*** |
| Facility open `24x7` | 1.45*** | 1.36*** | 1.08* |
| Number of Beds | 1.01*** | 1.05*** | 1.00 |
| Medical Officers | 1.05** | 1.10*** | 1.10*** |
| Lady medical Officers | 0.99 | 0.91 | 0.98 |
| Nurses | 1.01 | 1.07*** | 1.06*** |
| Lady Health Visitors | 1.07*** | 0.98 | 1.02 |
| Auxiliary Nurse and Midwifes | 1.04*** | 1.03 | 1.02* |
| Pharmacists | 0.97 | 1.03 | 1.06* |
| Medical Officer Residing | 1.12** | 1.33*** | 1.08** |
| Autoclave | 1.08 | 1.19** | 1.08* |
| Radiant Warmer | 1.28*** |  |  |
| Deep Freezer (large) |  | 0.97 | 1.07 |
| Ice Lined Refrigerator (Large) |  | 1.16 | 1.02 |
| Centrifuge |  | 1.19** | 1.19*** |
| Government Building | 0.97 | 1.17* | 1.03 |
| Building Condition | 0.95 | 0.95 | 1.01 |
| Water Availability | 1.11** | 0.94 | 1.05* |
| Toilet Availability | 0.75*** | 0.87* | 1.13*** |
|  |  |  |  |
| **State Fixed Effects** |  |  |  |
| Andhra Pradesh | 5.07*** |  |  |
| Arunachal Pradesh | 0.74 | 0.24*** | 0.29*** |
| Assam | 6.11*** | 0.30*** | 0.93 |
| Bihar | 23.10*** | 5.34*** | 1.87*** |
| Chhattisgarh | 3.42*** | 0.69* | 0.45*** |
| Goa | 4.94*** | 0.51* | 0.69* |
| Haryana | 5.95*** | 0.94 | 1.03 |
| Himachal Pradesh | 1.97* | 0.20*** | 0.66** |
| Jharkhand | 8.32*** | 0.76 | 0.60*** |
| Karnataka | 3.77*** | 0.97 | 0.52*** |
| Kerala | 6.75*** | 2.66*** | 0.85 |
| Madhya Pradesh | 8.37*** | 0.87 | 0.42*** |
| Maharashtra | 3.42*** | 1.37 | 0.08*** |
| Manipur | 1.73 | 0.91 | 0.19*** |
| Meghalaya | 2.54*** | 0.65* | 0.49*** |
| Mizoram | 1.51 | 0.44*** | 0.22*** |
| Nagaland | 0.94 | 0.21** | 0.18*** |
| Odisha | 4.97*** | 1.10 | 1.17 |
| Puducherry | 13.59*** |  |  |
| Punjab | 4.64*** | 0.0000 | 0.22*** |
| Rajasthan | 3.81*** |  |  |
| Sikkim | 1.31 | 0.54** | 0.42*** |
| Tamil Nadu | 4.27*** | 10.14*** | 3.94*** |
| Telangana | 3.12*** | 1.49* | 1.35** |
| Tripura | 2.81*** | 1.16 | 0.49*** |
| Uttar Pradesh | 7.40*** | 1.10 | 0.78** |
| Uttarakhand | 2.53*** | 0.59** | 0.51*** |
| West Bengal | 2.87*** | 0.56 | 2.62*** |
|  |  |  |  |
| **Interaction Effects** |  |  |  |
| Irregular Electricity: Generator | 0.92 | 1.02 | 0.89** |
| No Electricity: Generator | 2.10*** | 1.78* | 0.89 |
| Irregular Electricity:`24x7` | 1.04 | 0.98 | 1.00 |
| No Electricity:`24x7` | 1.46** | 0.58** | 0.88 |
| Irregular Electricity: Medical Officers | 0.97 | 1.00 | 1.02 |
| No Electricity: Medical Officers | 0.77*** | 0.97 | 1.10** |
| Irregular Electricity: Lady medical Officers | 0.96 | 1.13 | 1.03 |
| No Electricity: Lady medical Officers | 0.70 | 0.54* | 0.97 |
| Irregular Electricity: Nurses | 1.06*** | 1.01 | 0.98 |
| No Electricity: Nurses | 1.12 | 0.95 | 0.98 |
| Irregular Electricity: Lady Health Visitors | 0.98 | 1.17** | 1.02 |
| No Electricity: Lady Health Visitors | 1.60*** | 1.36 | 0.97 |
| Irregular Electricity: Auxiliary Nurse and Midwifes | 0.98* | 0.95** | 1.01 |
| No Electricity: Auxiliary Nurse and Midwifes | 1.21*** | 0.89 | 1.09*** |
| Irregular Electricity: Pharmacists | 1.10** | 1.02 | 1.03 |
| No Electricity: Pharmacists | 0.94 | 0.77 | 1.26*** |
| Irregular Electricity: Medical Officer Residing | 1.22*** | 1.02 | 1.06 |
| No Electricity: Medical Officer Residing | 1.03 | 0.78 | 0.98 |
| Irregular Electricity: Autoclave | 1.02 | 0.85 | 0.97 |
| No Electricity: Autoclave | 0.99 | 0.71* | 1.05 |
| Irregular Electricity: Radiant Warmer | 1.11 |  |  |
| No Electricity: Radiant Warmer | 1.53 |  |  |
| Irregular Electricity: Deep Freezer (large) |  | 1.13 | 0.99 |
| No Electricity: Deep Freezer (large) |  | 1.52 | 1.51 |
| Irregular Electricity: Ice Lined Refrigerator (Large) |  | 0.95 | 1.03 |
| No Electricity: Ice Lined Refrigerator (Large) |  | 0.67 | 0.82 |
| Irregular Electricity: Centrifuge |  | 1.06 | 0.97 |
| No Electricity: Centrifuge |  | 1.27 | 0.91 |
|  |  |  |  |
| Constant | 1.74* | 12.19*** | 513.16*** |
|  | | | |
| Observations | 7,805 | 4,540 | 4,782 |
| Log Likelihood | -22,415.63 | -14,396.67 | -35,832.35 |
| theta |  |  | 1.87*** (0.04) |
| Akaike Inf. Crit. |  |  | 71,812.70 |
|  | | | |
| Note: | ^*^p<0.1, ^**^p<0.5, ^***^p<0.01 | | |

**Table A2: Sensitivity analysis - Incidence Rate Ratios (IRR) (exponentiated log-odds) for the focal variables in the regression models for Deliveries outcome variable, under different outlier threshold conditions.**

| **Dependent Variable** | **Deliveries (DEL)** | | | | |
| --- | --- | --- | --- | --- | --- |
|  |  |  |  |  |  |
| **Condition** | No Limit | DEL<300 | DEL<200 | DEL<100 | DEL<50 |
|  |  |  |  |  |  |
| **Primary Focal Variables** |  |  |  |  |  |
| Irregular Electricity | 0.97 | 0.96 | 0.95 | 0.91 | 1.00 |
| No Electricity | 0.36^**^ | 0.37^**^ | 0.33^**^ | 0.36^***^ | 0.39^***^ |
|  |  |  |  |  |  |
| **Control for Interactions** | Yes | Yes | Yes | Yes | Yes |
| **Control for Covariates** | Yes | Yes | Yes | Yes | Yes |
| **State Fixed Effects** | Yes | Yes | Yes | Yes | Yes |
|  |  |  |  |  |  |
| Percent of PHCs excluded | 0 | 0.5 | 1.3 | 3.6 | 6.9 |
| Observations | 7,805 | 7,768 | 7,701 | 7,524 | 7,265 |
| Log Likelihood | -22,416 | -22,105 | -21,635 | -20,339 | -18,413 |
| *Note:* | ^*^p < 0.1, ^**^p < 0.05, ^***^p<0.01 | | | | |

**Table A3: Sensitivity analysis - Incidence Rate Ratios (IRR) (exponentiated log-odds) for the focal variables in the regression models for Out-Patient outcome variable, under different outlier threshold conditions.**

| **Dependent Variable** | **Out-Patient (OP)** | | | | |
| --- | --- | --- | --- | --- | --- |
|  |  |  |  |  |  |
| **Condition** | No Limit | OP<6000 | OP<5000 | OP<4000 | OP<3000 |
|  |  |  |  |  |  |
| **Primary Focal Variables** |  |  |  |  |  |
| Irregular Electricity | 0.94 | 0.99 | 0.98 | 0.97 | 0.96 |
| No Electricity | 0.62^***^ | 0.70^***^ | 0.70^***^ | 0.70^***^ | 0.73^***^ |
|  |  |  |  |  |  |
| **Control for Interactions** | Yes | Yes | Yes | Yes | Yes |
| **Control for Covariates** | Yes | Yes | Yes | Yes | Yes |
| **State Fixed Effects** | Yes | Yes | Yes | Yes | Yes |
|  |  |  |  |  |  |
| Percent of PHCs excluded | 0 | 0.4 | 1.3 | 3.1 | 3.8 |
| Observations | 4,782 | 4,708 | 4,665 | 4,610 | 4,505 |
| Log Likelihood | -35,832 | -35,007 | -34,586 | -34,042 | -33,043 |
| *Note:* | ^*^p < 0.1, ^**^p < 0.05, ^***^p<0.01 | | | | |

**Table A4: Incidence Rate Ratios (IRR) (exponentiated log-odds) for the focal variables with original five categories in the regression models for three outcome variables of interest.**

| **Dependent Variable** | **Deliveries** | **In-Patient** | **Out-Patient** |
| --- | --- | --- | --- |
|  |  |  |  |
| **Model** | Zero-Inflated  Negative Binomial | Zero-Inflated  Negative Binomial | Negative Binomial |
|  |  |  |  |
| **Focal Variables** |  |  |  |
| Occasional Power Cut | 0.94 | 1.07 | 1.05 |
| Summer Power Cut | 0.64 | 0.16 | 0.99 |
| Regular Power Cut | 1.02 | 1.35 | 0.82^***^ |
| No Electricity | 0.36^***^ | 1.77 | 0.62^***^ |
|  |  |  |  |
| **Control for Interactions** | Yes | Yes | Yes |
| **Control for Covariates** | Yes | Yes | Yes |
| **State Fixed Effects** | Yes | Yes | Yes |
|  |  |  |  |
| Observations | 7,805 | 4,540 | 4,782 |
| Log Likelihood | -22,398 | -14,371 | -35,818 |
| *Note:* | ^*^p < 0.1, ^**^p < 0.05, ^***^p<0.01 | | |
