# Supplementary material for: Reduced health services at under-electrified primary healthcare facilities: Evidence from India
Source: PLoS One. 2021 Jun 4;16(6):e0252705. doi: 10.1371/journal.pone.0252705 (PMC8177862; doi:10.1371/journal.pone.0252705)
Supplement: S1 Replication materials — (ZIP) [file pone.0252705.s002.zip › Replication material - PLOS ONE Review - Revised/Results/All_Models.html]

**All Models**

|  | | | |
|  | *Dependent variable:* | | |
|  |  | | |
|  | Deliveries | IPD | OPD |
|  | *zero-inflated* | *zero-inflated* | *negative* |
|  | *count data* | *count data* | *binomial* |
|  | (1) | (2) | (3) |
|  | | | |
| ElectricityIrregular Electricity | 0.97 | 1.05 | 0.94 |
| ElectricityNo Electricity | 0.36\*\*\* | 1.52\* | 0.62\*\*\* |
| Generator | 1.03 | 1.22\*\* | 1.25\*\*\* |
| Urban | 0.78\*\*\* | 0.81\*\* | 0.95 |
| Population10000 | 1.05\*\*\* | 1.02\*\*\* | 1.02\*\*\* |
| `24x7` | 1.45\*\*\* | 1.36\*\*\* | 1.08\* |
| Beds | 1.01\*\*\* | 1.05\*\*\* | 1.00 |
| MO\_Total | 1.04\*\* | 1.10\*\*\* | 1.10\*\*\* |
| LMO\_Total | 1.00 | 0.91 | 0.98 |
| Nurse\_Total | 1.01 | 1.07\*\*\* | 1.06\*\*\* |
| LHV\_Total | 1.07\*\*\* | 0.98 | 1.02 |
| ANM\_Total | 1.04\*\*\* | 1.03 | 1.02\* |
| Pharma\_Total | 0.97 | 1.03 | 1.06\* |
| MO\_Residing | 1.12\*\* | 1.33\*\*\* | 1.08\*\* |
| Autoclave | 1.07 | 1.19\*\* | 1.08\* |
| RadiantWarmer | 1.28\*\*\* |  |  |
| DF\_Large |  | 0.97 | 1.07 |
| ILR\_Large |  | 1.16 | 1.02 |
| Centrifuge |  | 1.19\*\* | 1.19\*\*\* |
| Govt\_Building | 0.97 | 1.17\* | 1.03 |
| Condition | 0.95 | 0.95 | 1.01 |
| Water | 1.11\*\* | 0.94 | 1.05\* |
| Toilet | 0.75\*\*\* | 0.87\* | 1.13\*\*\* |
| StateAndra Pradesh | 5.04\*\*\* |  |  |
| StateArunachal Pradesh | 0.74 | 0.24\*\*\* | 0.29\*\*\* |
| StateAssam | 6.10\*\*\* | 0.30\*\*\* | 0.93 |
| StateBihar | 22.80\*\*\* | 5.34\*\*\* | 1.87\*\*\* |
| StateChhattisgarh | 3.42\*\*\* | 0.69\* | 0.45\*\*\* |
| StateGoa | 4.93\*\*\* | 0.51\* | 0.69\* |
| StateHaryana | 5.94\*\*\* | 0.94 | 1.03 |
| StateHimachal Pradesh | 1.97\* | 0.20\*\*\* | 0.66\*\* |
| StateJharkhand | 8.29\*\*\* | 0.76 | 0.60\*\*\* |
| StateKarnataka | 3.76\*\*\* | 0.97 | 0.52\*\*\* |
| StateKerala | 6.73\*\*\* | 2.66\*\*\* | 0.85 |
| StateMadhya Pradesh | 8.34\*\*\* | 0.87 | 0.42\*\*\* |
| StateMaharashtra | 3.41\*\*\* | 1.37 | 0.08\*\*\* |
| StateManipur | 1.73 | 0.91 | 0.19\*\*\* |
| StateMeghalaya | 2.54\*\*\* | 0.65\* | 0.49\*\*\* |
| StateMizoram | 1.51 | 0.44\*\*\* | 0.22\*\*\* |
| StateNagaland | 0.94 | 0.21\*\* | 0.18\*\*\* |
| StateOdisha | 4.96\*\*\* | 1.10 | 1.17 |
| StatePuducherry | 13.59\*\*\* |  |  |
| StatePunjab | 4.63\*\*\* | 0.0000 | 0.22\*\*\* |
| StateRajasthan | 3.81\*\*\* |  |  |
| StateSikkim | 1.31 | 0.54\*\* | 0.42\*\*\* |
| StateTamil Nadu | 4.26\*\*\* | 10.14\*\*\* | 3.94\*\*\* |
| StateTelangana | 3.15\*\*\* | 1.49\* | 1.35\*\* |
| StateTripura | 2.81\*\*\* | 1.16 | 0.49\*\*\* |
| StateUttar Pradesh | 7.38\*\*\* | 1.10 | 0.78\*\* |
| StateUttrakhand | 2.52\*\*\* | 0.59\*\* | 0.51\*\*\* |
| StateWest Bengal | 2.86\*\*\* | 0.56 | 2.62\*\*\* |
| ElectricityIrregular Electricity:Generator | 0.91 | 1.02 | 0.89\*\* |
| ElectricityNo Electricity:Generator | 2.10\*\*\* | 1.78\* | 0.89 |
| ElectricityIrregular Electricity:`24x7` | 1.04 | 0.98 | 1.00 |
| ElectricityNo Electricity:`24x7` | 1.46\*\* | 0.58\*\* | 0.88 |
| ElectricityIrregular Electricity:MO\_Total | 0.97 | 1.00 | 1.02 |
| ElectricityNo Electricity:MO\_Total | 0.77\*\*\* | 0.97 | 1.10\*\* |
| ElectricityIrregular Electricity:LMO\_Total | 0.96 | 1.13 | 1.03 |
| ElectricityNo Electricity:LMO\_Total | 0.70 | 0.54\* | 0.97 |
| ElectricityIrregular Electricity:Nurse\_Total | 1.06\*\*\* | 1.01 | 0.98 |
| ElectricityNo Electricity:Nurse\_Total | 1.12 | 0.95 | 0.98 |
| ElectricityIrregular Electricity:LHV\_Total | 0.98 | 1.17\*\* | 1.02 |
| ElectricityNo Electricity:LHV\_Total | 1.60\*\*\* | 1.36 | 0.97 |
| ElectricityIrregular Electricity:ANM\_Total | 0.98\* | 0.95\*\* | 1.01 |
| ElectricityNo Electricity:ANM\_Total | 1.21\*\*\* | 0.89 | 1.09\*\*\* |
| ElectricityIrregular Electricity:Pharma\_Total | 1.10\*\* | 1.02 | 1.03 |
| ElectricityNo Electricity:Pharma\_Total | 0.94 | 0.77 | 1.26\*\*\* |
| ElectricityIrregular Electricity:MO\_Residing | 1.22\*\*\* | 1.02 | 1.06 |
| ElectricityNo Electricity:MO\_Residing | 1.03 | 0.78 | 0.98 |
| ElectricityIrregular Electricity:Autoclave | 1.02 | 0.85 | 0.97 |
| ElectricityNo Electricity:Autoclave | 0.99 | 0.71\* | 1.05 |
| ElectricityIrregular Electricity:RadiantWarmer | 1.11 |  |  |
| ElectricityNo Electricity:RadiantWarmer | 1.53 |  |  |
| ElectricityIrregular Electricity:DF\_Large |  | 1.13 | 0.99 |
| ElectricityNo Electricity:DF\_Large |  | 1.52 | 1.51 |
| ElectricityIrregular Electricity:ILR\_Large |  | 0.95 | 1.03 |
| ElectricityNo Electricity:ILR\_Large |  | 0.67 | 0.82 |
| ElectricityIrregular Electricity:Centrifuge |  | 1.06 | 0.97 |
| ElectricityNo Electricity:Centrifuge |  | 1.27 | 0.91 |
| Constant | 1.75\* | 12.19\*\*\* | 513.16\*\*\* |
|  | | | |
| Observations | 7,802 | 4,540 | 4,782 |
| Log Likelihood | -22,406.50 | -14,396.67 | -35,832.35 |
| theta |  |  | 1.87\*\*\* (0.04) |
| Akaike Inf. Crit. |  |  | 71,812.70 |
|  | | | |
| *Note:* | \*p<0.1; \*\*p<0.05; \*\*\*p<0.01 | | |
