# Supplementary material for: Reduced health services at under-electrified primary healthcare facilities: Evidence from India
Source: PLoS One. 2021 Jun 4;16(6):e0252705. doi: 10.1371/journal.pone.0252705 (PMC8177862; doi:10.1371/journal.pone.0252705)
Supplement: S1 Replication materials — (ZIP) [file pone.0252705.s002.zip › Replication material - PLOS ONE Review - Revised/Results/All_Models_District FE.html]

**All ModelsFE**

|  | | | |
|  | *Dependent variable:* | | |
|  |  | | |
|  | Deliveries | IPD | OPD |
|  | *zero-inflated* | *zero-inflated* | *negative* |
|  | *count data* | *count data* | *binomial* |
|  | (1) | (2) | (3) |
|  | | | |
| ElectricityIrregular Electricity | 0.90 | 0.85 | 0.97 |
| ElectricityNo Electricity | 0.38 | 1.42 | 0.68\*\*\* |
| Generator | 1.05 | 1.29 | 1.23\*\*\* |
| Urban | 0.80 | 0.87 | 0.94 |
| Population10000 | 1.05 | 1.03 | 1.02\*\*\* |
| `24x7` | 1.47 | 1.42 | 1.19\*\*\* |
| Beds | 1.02 | 1.03 | 1.01\*\*\* |
| MO\_Total | 1.08 | 1.16 | 1.13\*\*\* |
| LMO\_Total | 0.97 | 0.92 | 1.04 |
| Nurse\_Total | 1.05 | 1.16 | 1.04\*\* |
| LHV\_Total | 1.05 | 1.02 | 1.04 |
| ANM\_Total | 1.04 | 1.00 | 1.02\*\* |
| Pharma\_Total | 1.01 | 1.04 | 1.06\* |
| MO\_Residing | 1.20 | 1.35 | 1.08\*\* |
| Autoclave | 1.08 | 1.00 | 1.04 |
| RadiantWarmer | 1.22 |  |  |
| DF\_Large |  | 1.01 | 1.06 |
| ILR\_Large |  | 1.17 | 1.03 |
| Centrifuge |  | 1.21 | 1.17\*\*\* |
| Govt\_Building | 0.95 | 0.99 | 0.99 |
| Condition | 0.92 | 0.94 | 1.01 |
| Water | 1.12 | 1.05 | 1.06\*\* |
| Toilet | 0.83 | 0.98 | 1.05 |
| DistrictAgra | 0.49 |  |  |
| DistrictAhmadnagar | 0.44 |  |  |
| DistrictAizawl | 0.27 | 1.04 | 0.34\*\* |
| DistrictAjmer | 0.78 |  |  |
| DistrictAkola | 0.67 |  |  |
| DistrictAlappuzha | 0.00 | 0.00 | 5.96\*\*\* |
| DistrictAligarh | 1.45 | 1.90 | 1.31 |
| DistrictAllahabad | 0.65 | 15.94 | 2.31\*\*\* |
| DistrictAlmora | 0.55 | 1.12 | 0.67 |
| DistrictAlwar | 0.24 |  |  |
| DistrictAmbala | 0.96 | 5.30 | 2.02\*\* |
| DistrictAmbedkar Nagar | 0.83 | 12.80 | 1.08 |
| DistrictAmrawati | 0.51 |  |  |
| DistrictAmritsar | 1.06 |  |  |
| DistrictAnantpur | 1.36 |  |  |
| DistrictAnjaw | 0.07 | 0.22 | 0.27\*\*\* |
| DistrictAnugul | 1.43 | 2.16 | 1.68\* |
| DistrictAraria | 3.65 | 2.25 | 2.09\*\* |
| DistrictAriyalur | 1.65 | 23.31 | 6.63\*\*\* |
| DistrictAuraiya | 0.95 | 2.05 | 1.72\*\* |
| DistrictAurangabad | 1.41 | 9.78 | 2.53\*\*\* |
| DistrictAzamgarh | 1.23 | 2.99 | 1.18 |
| DistrictBagalkot | 0.77 | 2.16 | 0.94 |
| DistrictBageshwar | 0.11 | 2.01 | 0.43\*\* |
| DistrictBaghpat | 1.38 | 0.37 | 1.48 |
| DistrictBahraich | 3.79 | 5.68 | 1.73\*\* |
| DistrictBalaghat | 1.65 | 2.28 | 0.64\* |
| DistrictBalangir | 0.46 | 0.14 | 1.59\* |
| DistrictBaleshwar | 0.49 | 1.76 | 3.04\*\*\* |
| DistrictBallia | 0.50 | 0.56 | 0.66 |
| DistrictBalrampur | 2.90 | 7.64 | 2.13\* |
| DistrictBanda | 1.75 | 1.65 | 0.85 |
| DistrictBangalore | 1.35 | 2.52 | 1.05 |
| DistrictBangalore Rural | 0.41 | 1.14 | 1.11 |
| DistrictBanka | 13.16 | 29.97 | 3.58\*\*\* |
| DistrictBankura | 0.08 |  | 3.62\*\*\* |
| DistrictBanswara | 1.35 |  |  |
| DistrictBarabanki | 1.97 | 4.58 | 1.41 |
| DistrictBaran | 1.05 |  |  |
| DistrictBarddhaman | 1.38 |  | 1.80 |
| DistrictBareilly | 1.08 | 5.72 | 0.96 |
| DistrictBargarh | 1.64 | 2.24 | 1.73\*\* |
| DistrictBarmer | 1.12 |  |  |
| DistrictBarnala | 0.73 |  |  |
| DistrictBarpeta | 2.70 | 7.15 | 2.06\*\*\* |
| DistrictBarwani | 3.30 | 5.26 | 1.11 |
| DistrictBastar | 0.69 | 2.45 | 0.70 |
| DistrictBasti | 0.95 | 3.54 | 1.10 |
| DistrictBaudh | 0.20 | 0.00 | 2.73\*\*\* |
| DistrictBegusarai | 5.86 | 6.74 | 2.46\*\*\* |
| DistrictBelgaum | 0.93 | 3.28 | 0.74 |
| DistrictBellary | 0.98 | 2.11 | 0.86 |
| DistrictBetul | 1.33 | 2.02 | 1.02 |
| DistrictBhadrak | 1.12 | 7.45 | 3.42\*\*\* |
| DistrictBhagalpur | 6.09 | 10.12 | 3.49\*\*\* |
| DistrictBhandara | 0.44 |  |  |
| DistrictBharatpur | 0.87 |  |  |
| DistrictBhathinda | 0.03 |  |  |
| DistrictBhilwara | 0.79 |  |  |
| DistrictBhind | 4.13 | 3.43 | 1.14 |
| DistrictBhiwani | 1.10 | 1.39 | 1.12 |
| DistrictBhojpur | 9.44 | 18.38 | 4.21\*\*\* |
| DistrictBhopal | 0.73 | 1.55 | 0.51\* |
| DistrictBid | 0.52 | 2.05 | 0.00 |
| DistrictBidar | 0.88 | 1.66 | 0.75 |
| DistrictBijapur | 0.94 | 3.79 | 0.90 |
| DistrictBijnor | 0.86 | 3.94 | 1.32 |
| DistrictBikaner | 1.30 |  |  |
| DistrictBilaspur | 0.62 | 1.26 | 0.63\*\* |
| DistrictBirbhum | 0.41 | 0.17 | 2.31\*\* |
| DistrictBishnupur | 0.02 | 7.40 | 0.42\*\* |
| DistrictBokaro | 1.39 | 0.31 | 0.72 |
| DistrictBongaigaon | 1.34 | 6.50 | 1.44 |
| DistrictBudaun | 1.52 | 0.0000 | 0.84 |
| DistrictBulandshahar | 2.94 | 4.70 | 1.49 |
| DistrictBuldana | 1.02 |  |  |
| DistrictBundi | 0.67 |  |  |
| DistrictBuxar | 5.85 | 14.37 | 2.81\*\*\* |
| DistrictCachar | 0.75 | 0.90 | 1.62\* |
| DistrictChamarajanagar | 0.48 | 2.63 | 0.85 |
| DistrictChamba | 0.41 | 0.00 | 0.88 |
| DistrictChamoli | 0.82 | 1.97 | 1.34 |
| DistrictChampawat | 0.81 | 1.88 | 0.73 |
| DistrictChamphai | 0.31 | 1.06 | 0.32\*\*\* |
| DistrictChandauli | 0.97 | 2.04 | 0.76 |
| DistrictChandel | 0.07 | 9.37 | 0.22\*\*\* |
| DistrictChandrapur | 0.42 |  |  |
| DistrictChanglang | 0.30 | 0.83 | 0.64 |
| DistrictChatra | 2.30 | 2.77 | 1.17 |
| DistrictChhatarpur | 1.06 | 1.44 | 0.23\*\*\* |
| DistrictChhindwara | 0.80 | 0.69 | 0.64\* |
| DistrictChikkaballarpura | 0.83 | 3.93 | 1.09 |
| DistrictChikmagalur | 0.19 | 0.93 | 0.71 |
| DistrictChitoor | 1.59 | 1.08 | 2.45 |
| DistrictChitradurga | 0.55 | 1.14 | 1.10 |
| DistrictChitrakoot | 2.93 | 5.76 | 1.59\* |
| DistrictChittaurgarh | 0.64 |  |  |
| DistrictChurachandpur | 0.23 | 0.61 | 0.32\*\*\* |
| DistrictChuru | 0.61 |  |  |
| DistrictCoimbatore | 0.63 |  |  |
| DistrictCuddalore | 0.59 |  |  |
| DistrictCuttack | 1.12 | 8.41 | 2.14\*\*\* |
| DistrictDakshin Dinajpur | 0.91 |  | 6.24\*\*\* |
| DistrictDakshina Kannada | 1.53 | 2.40 | 1.08 |
| DistrictDamoh | 2.78 | 3.23 | 0.50\*\* |
| DistrictDantewada | 0.54 | 2.61 | 0.97 |
| DistrictDarbhanga | 2.46 | 13.77 | 5.28\*\*\* |
| DistrictDarjiling | 0.25 |  | 0.74 |
| DistrictDarrang | 1.91 | 4.26 | 1.49 |
| DistrictDatai | 1.14 | 1.94 | 0.80 |
| DistrictDausa | 0.89 |  |  |
| DistrictDavangere | 1.09 | 3.11 | 0.74 |
| DistrictDebagarh | 0.71 | 1.99 | 1.63 |
| DistrictDehradun | 0.45 | 1.19 | 0.90 |
| DistrictDeoria | 0.98 | 3.25 | 0.71 |
| DistrictDewas | 2.81 | 2.37 | 0.38\*\*\* |
| DistrictDhalai | 0.54 | 2.44 | 0.95 |
| DistrictDhamtari | 0.52 | 1.48 | 0.98 |
| DistrictDhanbad | 1.59 | 3.09 | 1.15 |
| DistrictDhar | 1.61 | 2.35 | 0.63\* |
| DistrictDharmapuri | 1.72 |  |  |
| DistrictDharwad | 0.64 | 2.38 | 1.18 |
| DistrictDhaulpur | 0.46 |  |  |
| DistrictDhemaji | 1.62 | 0.04 | 1.52 |
| DistrictDhenkanal | 0.93 | 2.43 | 1.71\*\* |
| DistrictDhubri | 1.02 | 0.78 | 2.80\*\*\* |
| DistrictDhule | 0.77 |  |  |
| DistrictDibang Valley | 0.12 | 0.71 | 0.51 |
| DistrictDibrugarh | 0.37 | 0.18 | 1.47 |
| DistrictDimapur | 0.27 | 0.33 | 0.22\*\*\* |
| DistrictDindigul | 1.38 |  |  |
| DistrictDindori | 0.66 | 1.27 | 0.51\*\* |
| DistrictDumka | 0.50 | 0.62 | 0.64 |
| DistrictDungarpur | 0.97 |  |  |
| DistrictDurg | 0.45 | 1.34 | 0.60\*\* |
| DistrictEast | 0.26 | 1.35 | 0.70 |
| DistrictEast Garo Hills | 0.39 | 1.86 | 0.71 |
| DistrictEast Godavari | 1.05 | 5.86 | 1.96\* |
| DistrictEast Kameng | 0.06 | 0.50 | 0.13\*\*\* |
| DistrictEast Khasi Hills | 3.78 | 11.27 | 1.13 |
| DistrictEast Nimar | 0.97 | 1.01 | 0.47\*\*\* |
| DistrictEast Siang | 0.14 | 1.98 | 0.47\*\* |
| DistrictErode | 0.27 |  |  |
| DistrictEtah | 8.00 | 3.63 | 1.36 |
| DistrictEtawah | 3.77 | 2.60 | 2.26\*\*\* |
| DistrictFaizabad | 6.07 | 3.64 | 2.67\*\*\* |
| DistrictFaridabad | 0.85 | 1.84 | 1.80\* |
| DistrictFaridkot | 0.69 |  |  |
| DistrictFarrukhabad | 0.24 | 2.79 | 2.19\*\*\* |
| DistrictFatehabad | 1.73 | 1.71 | 1.35 |
| DistrictFatehgarh Sahib | 0.57 |  |  |
| DistrictFatehpur | 0.29 | 1.79 | 1.17 |
| DistrictFirozabad | 8.22 | 0.45 | 1.05 |
| DistrictFirozpur | 0.79 |  |  |
| DistrictGadag | 0.76 | 2.85 | 0.77 |
| DistrictGadchiroli | 0.75 |  |  |
| DistrictGajapati | 1.02 | 2.74 | 1.86\*\* |
| DistrictGanganagar | 0.88 |  |  |
| DistrictGanjam | 3.48 | 1.04 | 1.74\*\* |
| DistrictGarhwa | 1.89 | 1.60 | 1.08 |
| DistrictGarhwal | 0.29 | 1.20 | 1.00 |
| DistrictGautam Buddha Nagar | 1.46 | 3.75 | 1.41 |
| DistrictGaya | 4.74 | 13.14 | 2.73\*\*\* |
| DistrictGhaziabad | 0.04 | 1.04 | 0.87 |
| DistrictGhazipur | 2.70 | 1.56 | 1.54 |
| DistrictGiridih | 1.22 | 0.83 | 0.29\*\* |
| DistrictGoalpara | 0.97 | 1.32 | 1.66\*\* |
| DistrictGodda | 1.37 | 2.99 | 0.61 |
| DistrictGolaghat | 0.64 | 0.55 | 1.20 |
| DistrictGonda | 1.67 | 2.50 | 1.92\*\* |
| DistrictGondiya | 0.57 |  |  |
| DistrictGopalganj | 6.13 | 18.51 | 4.06\*\*\* |
| DistrictGorakhpur | 0.96 | 1.68 | 1.12 |
| DistrictGulbarga | 0.81 | 2.87 | 0.71 |
| DistrictGuna | 1.64 | 2.90 | 0.38\*\*\* |
| DistrictGuntur | 0.77 |  |  |
| DistrictGurgaon | 2.02 | 3.80 | 2.24\*\*\* |
| DistrictGwalior | 0.61 | 1.50 | 0.58\* |
| DistrictHailakandi | 0.32 | 0.40 | 1.86 |
| DistrictHamirpur | 0.83 | 4.78 | 0.95 |
| DistrictHamumagarh | 0.34 |  |  |
| DistrictHaora | 1.57 | 1.39 | 5.08\*\*\* |
| DistrictHarda | 0.89 | 4.31 | 0.21\*\*\* |
| DistrictHardoi | 12.76 | 3.29 | 0.75 |
| DistrictHardwar | 1.14 | 0.77 | 2.62\*\* |
| DistrictHassan | 0.16 | 0.34 | 0.40\*\*\* |
| DistrictHathras | 0.25 | 1.70 | 1.41 |
| DistrictHaveri | 0.72 | 3.16 | 0.91 |
| DistrictHazaribagh | 1.49 | 1.84 | 1.54 |
| DistrictHingoli | 0.95 | 1.57 | 0.24\*\* |
| DistrictHisar | 0.96 | 1.83 | 1.61\* |
| DistrictHoshangabad | 0.60 | 1.90 | 0.41\*\*\* |
| DistrictHoshiarpur | 0.97 | 0.00 | 0.00 |
| DistrictHugli | 0.28 |  |  |
| DistrictIdukki | 1.44 | 9.72 | 0.51\*\* |
| DistrictImphal East | 0.05 | 0.02 | 0.26\*\*\* |
| DistrictImphal West | 2.15 | 0.43 | 0.45\*\* |
| DistrictIndore | 1.60 | 1.61 | 1.61\* |
| DistrictJabalpur | 0.89 | 1.31 | 0.46\*\*\* |
| DistrictJagatsinghapur | 0.00 | 0.0000 | 1.78\*\* |
| DistrictJaintia Hills | 0.26 | 0.77 | 0.62\* |
| DistrictJaipur | 1.25 |  |  |
| DistrictJaisalmer | 0.77 |  |  |
| DistrictJajapur | 0.72 | 1.67 | 2.40\*\*\* |
| DistrictJalandhar | 1.08 |  |  |
| DistrictJalaun | 0.80 | 4.37 | 1.29 |
| DistrictJalgaon | 0.64 |  |  |
| DistrictJalna | 0.57 |  |  |
| DistrictJalore | 0.82 |  |  |
| DistrictJalpaiguri | 0.33 | 1.51 | 4.65\*\*\* |
| DistrictJamui | 6.98 | 16.17 | 3.41\*\*\* |
| DistrictJanjgir - Champa | 0.54 | 1.12 | 0.48\*\*\* |
| DistrictJashpur | 0.55 | 3.29 | 0.94 |
| DistrictJaunpur | 2.09 | 1.82 | 0.87 |
| DistrictJehanabad | 5.38 | 13.62 | 2.84\*\*\* |
| DistrictJhabua | 2.93 | 3.66 | 0.64 |
| DistrictJhajjar | 1.50 | 2.06 | 1.50 |
| DistrictJhalawar | 0.47 |  |  |
| DistrictJhansi | 0.45 | 1.33 | 0.61\* |
| DistrictJharsuguda | 0.39 | 2.70 | 1.49 |
| DistrictJhunjhunun | 0.39 |  |  |
| DistrictJind | 0.95 | 2.51 | 2.09\*\*\* |
| DistrictJodhpur | 1.39 |  |  |
| DistrictJorhat | 0.53 | 0.55 | 1.02 |
| DistrictJyotiba Phule Nagar | 3.06 | 4.44 | 1.50 |
| DistrictKaimur (Bhabua) | 6.66 | 12.85 | 3.23\*\*\* |
| DistrictKaithal | 1.06 | 2.42 | 2.16\*\*\* |
| DistrictKalahandi | 0.58 | 5.40 | 1.86\*\* |
| DistrictKamrup | 1.24 | 0.28 | 1.72\*\* |
| DistrictKancheepuram | 1.02 |  |  |
| DistrictKandhamal | 1.40 | 6.43 | 1.36 |
| DistrictKangra | 0.21 |  |  |
| DistrictKanker | 0.30 | 1.98 | 0.68 |
| DistrictKannauj | 1.95 | 2.38 | 1.27 |
| DistrictKanniyakumari | 0.44 |  |  |
| DistrictKannur | 3.31 | 0.00 | 2.55\*\*\* |
| DistrictKanpur Dehat | 0.0000 | 2.71 | 1.99\*\* |
| DistrictKanpur Nagar | 1.20 | 1.57 | 1.39 |
| DistrictKapurthala | 1.44 |  |  |
| DistrictKaraikal | 0.19 |  |  |
| DistrictKarauli | 0.73 |  |  |
| DistrictKarbi Anglong | 1.94 | 2.68 | 0.82 |
| DistrictKarimganj | 1.47 | 1.26 | 2.93\*\*\* |
| DistrictKarimnagar | 0.30 | 1.92 | 1.61 |
| DistrictKarnal | 1.13 | 2.06 | 1.67\* |
| DistrictKarur | 0.28 |  |  |
| DistrictKasaragod | 2.01 | 12.41 | 0.52\*\* |
| DistrictKatihar | 5.00 | 8.77 | 3.22\*\*\* |
| DistrictKatni | 2.21 | 3.10 | 0.56\* |
| DistrictKaushambi | 2.10 | 3.04 | 0.91 |
| DistrictKawardha | 1.02 | 4.01 | 0.89 |
| DistrictKendrapara | 0.97 | 2.50 | 2.20\*\*\* |
| DistrictKendujhar | 0.35 | 1.76 | 1.43 |
| DistrictKhagaria | 7.25 | 9.02 | 3.86\*\*\* |
| DistrictKhammam | 0.22 |  |  |
| DistrictKheri | 1.29 | 3.69 | 1.74\*\* |
| DistrictKhordha | 2.94 | 0.0000 | 2.43\*\*\* |
| DistrictKinnaur | 0.13 |  |  |
| DistrictKiphire | 0.26 |  |  |
| DistrictKishanganj | 2.12 | 3.75 | 2.97\*\*\* |
| DistrictKoch Bihar | 0.18 | 1.54 | 4.17\*\*\* |
| DistrictKodagu | 0.45 | 1.24 | 0.67 |
| DistrictKodarma | 2.30 | 2.85 | 0.75 |
| DistrictKohima | 0.11 | 0.49 | 0.38\*\* |
| DistrictKokrajhar | 2.63 | 0.0000 | 0.60\* |
| DistrictKolar | 0.67 | 1.06 | 1.08 |
| DistrictKOLASIB | 0.28 | 1.49 | 0.48\*\* |
| DistrictKolhapur | 0.78 |  |  |
| Districtkollam | 0.0000 | 0.00 | 4.13\*\*\* |
| DistrictKoppal | 0.99 | 2.58 | 0.88 |
| DistrictKoraput | 0.86 | 2.94 | 1.03 |
| DistrictKorba | 1.20 | 2.35 | 0.61\*\* |
| DistrictKoriya | 1.40 | 3.23 | 1.04 |
| DistrictKota | 0.58 |  |  |
| DistrictKozhikode | 0.00 |  |  |
| DistrictKrishna | 0.81 |  |  |
| DistrictKrishnagiri | 1.06 |  |  |
| DistrictKullu | 0.49 | 1.21 | 2.55\*\*\* |
| DistrictKurnool | 1.57 | 1.73 | 1.85\*\* |
| DistrictKurukshetra | 0.65 | 1.72 | 2.03\*\* |
| DistrictKurung Kamey | 0.00 | 0.15 | 0.47\* |
| DistrictKushinagar | 1.58 | 0.71 | 0.96 |
| DistrictLahul | Spiti | 0.08 | 0.03 |
| DistrictLakhimpur | 0.91 | 0.23 | 1.65\* |
| DistrictLakhisarai | 3.22 | 10.71 | 1.34 |
| DistrictLalitpur | 1.05 | 4.36 | 1.14 |
| DistrictLatur | 0.71 |  |  |
| DistrictLAWNGTLAI | 0.16 | 0.67 | 0.52 |
| DistrictLohardaga | 0.55 | 0.00 | 0.47 |
| DistrictLohit | 0.30 | 1.10 | 0.80 |
| DistrictLongleng | 0.27 |  |  |
| DistrictLower Dibang Valley | 0.05 | 0.17 | 0.56 |
| DistrictLower Subansiri | 0.09 | 0.61 | 0.52\* |
| DistrictLucknow | 3.59 | 2.59 | 1.71\*\* |
| DistrictLUNGLEI | 0.22 | 0.94 | 0.33\*\*\* |
| DistrictMadhepura | 4.09 | 4.74 | 3.21\*\*\* |
| DistrictMadhubani | 2.37 | 9.44 | 1.94\*\* |
| DistrictMadurai | 1.22 |  |  |
| DistrictMahasamund | 0.87 | 2.15 | 0.83 |
| DistrictMahbubnagar | 1.04 | 3.26 | 1.94\* |
| DistrictMahendragarh | 0.21 | 0.46 | 1.24 |
| DistrictMahoba | 0.90 | 2.64 | 0.71 |
| DistrictMahrajganj | 0.95 | 1.54 | 0.86 |
| DistrictMainpuri | 3.27 | 3.22 | 1.61\* |
| DistrictMaldah | 0.00 |  | 5.39\*\*\* |
| DistrictMalkangiri | 1.11 | 2.49 | 1.58 |
| DistrictMallappuram | 0.00 | 4.05 | 0.10\*\*\* |
| DistrictMamit | 0.22 | 1.48 | 0.31\*\*\* |
| DistrictMandi | 1.11 |  |  |
| DistrictMandla | 0.74 | 0.78 | 0.35\*\*\* |
| DistrictMandsaur | 1.35 | 3.84 | 0.42\*\*\* |
| DistrictMandya | 0.87 | 1.35 | 0.83 |
| DistrictMarigaon | 1.44 | 0.09 | 2.16\*\*\* |
| DistrictMathura | 2.17 | 2.10 | 1.25 |
| DistrictMau | 3.29 | 3.00 | 1.11 |
| DistrictMayurbhanj | 0.67 | 3.20 | 2.36\*\*\* |
| DistrictMedak | 0.52 | 4.75 | 2.09\*\* |
| DistrictMeerut | 0.47 | 1.28 | 1.56 |
| DistrictMewat | 1.95 | 12.99 | 2.05\*\* |
| DistrictMirzapur | 0.49 | 1.76 | 0.89 |
| DistrictMoga | 0.96 | 0.00 | 0.51 |
| DistrictMokokchung | 0.08 |  |  |
| DistrictMon | 0.04 |  |  |
| DistrictMoradabad | 0.72 | 3.94 | 1.08 |
| DistrictMorena | 3.96 | 3.53 | 1.05 |
| DistrictMuktsar | 0.90 |  |  |
| DistrictMunger | 5.14 | 5.93 | 2.92\*\*\* |
| DistrictMurshidabad | 1.73 |  |  |
| DistrictMuzaffarnagar | 1.05 | 2.47 | 1.46 |
| DistrictMuzaffarpur | 2.87 | 6.90 | 1.90\*\* |
| DistrictMysore | 0.41 | 2.32 | 0.93 |
| DistrictNabarangapur | 3.70 | 6.85 | 1.46 |
| DistrictNadia | 0.71 |  | 6.28\*\*\* |
| DistrictNagaon | 2.69 | 0.00 | 1.85\*\* |
| DistrictNagapattinam | 0.23 |  |  |
| DistrictNagaur | 0.90 |  |  |
| DistrictNagpur | 0.68 |  |  |
| DistrictNainital | 0.58 | 1.27 | 0.67 |
| DistrictNalanda | 2.47 |  | 2.97\*\*\* |
| DistrictNalbari | 0.44 | 0.01 | 2.00\*\* |
| DistrictNalgonda | 0.47 | 5.47 | 2.94\*\*\* |
| DistrictNamakkal | 0.46 |  |  |
| DistrictNanded | 1.29 |  |  |
| DistrictNandurbar | 0.75 |  |  |
| DistrictNarsimhapur | 1.33 | 2.14 | 1.58\* |
| DistrictNashik | 0.61 |  |  |
| DistrictNawada | 6.92 | 16.56 | 3.94\*\*\* |
| DistrictNayagarh | 0.71 | 0.47 | 2.36\*\*\* |
| DistrictNeemuch | 0.73 | 1.18 | 0.39\*\*\* |
| DistrictNicobar | 0.12 |  |  |
| DistrictNilgiris | 0.78 |  |  |
| DistrictNizamabad | 0.78 | 5.35 | 2.32 |
| DistrictNorth | 0.10 | 0.39 | 0.36\*\*\* |
| DistrictNorth | Middle Andaman | 0.19 |  |
| DistrictNorth 24 Parganas | 0.00 |  |  |
| DistrictNorth Cachar Hills | 0.24 | 0.02 | 0.53 |
| DistrictNorth Goa | 0.84 | 0.96 | 1.14 |
| DistrictNorth Tripura | 0.49 | 3.26 | 0.84 |
| DistrictNuapada | 0.83 | 0.75 | 1.28 |
| DistrictOsmanabad | 0.57 |  |  |
| DistrictPALAKKAD | 1.30 | 4.45 | 0.51\*\* |
| DistrictPalamu | 3.64 | 5.24 | 0.63 |
| DistrictPali | 0.69 |  |  |
| DistrictPalwal | 3.63 | 4.29 | 2.81\*\*\* |
| DistrictPanchkula | 1.15 | 2.48 | 2.73\*\*\* |
| DistrictPanipath | 0.56 |  |  |
| DistrictPanna | 1.93 | 2.11 | 0.47\*\* |
| DistrictPapumpare | 0.00 | 0.00 | 0.15\*\*\* |
| DistrictParbhani | 0.77 |  |  |
| DistrictParen | 0.24 |  |  |
| DistrictPaschim Medinipur | 0.49 |  | 2.15 |
| DistrictPashchim Champaran | 3.69 | 9.53 | 3.04\*\*\* |
| DistrictPashchimi Singhbhum | 2.57 | 4.24 | 1.52 |
| DistrictPathanamthitta | 1.11 |  |  |
| DistrictPatiala | 0.41 |  |  |
| DistrictPatna | 3.12 | 6.48 | 2.45\*\*\* |
| DistrictPerambalur | 0.74 |  |  |
| DistrictPhek | 0.21 |  |  |
| DistrictPilibhit | 0.73 | 1.81 | 0.61 |
| DistrictPithoragarh | 0.44 | 1.55 | 1.05 |
| DistrictPondicherry | 4.43 |  |  |
| DistrictPrakasam | 0.27 | 1.13 | 1.24 |
| DistrictPratapgarh | 1.22 | 3.20 | 1.44 |
| DistrictPudukkottai | 1.22 |  |  |
| DistrictPune | 0.89 | 6.05 |  |
| DistrictPurba Champaran | 2.53 | 3.46 | 2.19\*\*\* |
| DistrictPurba Mednipur | 0.23 | 1.04 | 3.13\*\*\* |
| DistrictPurbi Singhbhum | 0.00 | 0.00 | 0.49 |
| DistrictPuri | 0.20 | 3.20 | 1.90\*\*\* |
| DistrictPurnia | 3.63 | 7.75 | 2.14\*\* |
| DistrictPuruliya | 0.49 |  | 2.03 |
| DistrictRae Bareli | 0.21 | 3.36 | 1.07 |
| DistrictRaichur | 0.83 | 2.40 | 0.75 |
| DistrictRaigarh | 0.56 | 1.31 | 0.64\* |
| DistrictRaipur | 0.71 | 1.65 | 0.84 |
| DistrictRaisen | 1.69 | 2.10 | 0.64 |
| DistrictRajgarh | 3.26 | 3.94 | 0.56\*\* |
| DistrictRajnandgaon | 1.22 | 2.98 | 0.91 |
| DistrictRajsamand | 0.50 |  |  |
| DistrictRamanagara | 0.34 | 8.60 | 0.87 |
| DistrictRamanathapuram | 0.43 |  |  |
| DistrictRampur | 0.25 | 1.38 | 2.74\*\*\* |
| DistrictRanchi | 0.48 | 0.29 | 1.04 |
| DistrictRangareddy | 0.43 | 2.46 | 2.16\*\*\* |
| DistrictRatlam | 3.46 | 4.81 | 0.88 |
| DistrictRatnagiri | 0.29 |  |  |
| DistrictRayagada | 1.01 | 5.62 | 2.05\*\*\* |
| DistrictRewa | 1.04 | 1.31 | 0.78 |
| DistrictRewari | 0.68 | 1.57 | 1.52 |
| DistrictRi Bhoi | 0.41 | 2.18 | 0.88 |
| DistrictRohtak | 0.62 | 1.26 | 1.63\* |
| DistrictRohtas | 6.00 | 16.70 | 3.70\*\*\* |
| DistrictRudraprayag | 1.01 | 0.86 | 1.12 |
| DistrictRupnagar | 0.89 |  |  |
| DistrictSagar | 1.98 | 2.49 | 0.50\*\* |
| DistrictSaharanpur | 0.16 | 2.97 | 1.78\*\* |
| DistrictSaharsa | 4.44 | 22.79 | 5.23\*\*\* |
| DistrictSahibganj | 1.98 | 2.40 | 0.73 |
| DistrictSAIHA | 0.46 | 1.14 | 0.39\*\*\* |
| DistrictSalem | 0.17 |  |  |
| DistrictSamastipur | 3.46 | 14.52 | 2.67\*\*\* |
| DistrictSambalpur | 0.83 | 1.71 | 2.70\*\*\* |
| DistrictSangli | 0.59 |  |  |
| DistrictSant Kabir Nagar | 0.93 | 2.42 | 1.99\*\* |
| DistrictSant Ravidas Nagar Bhadohi | 2.55 | 4.72 | 1.53 |
| DistrictSaran | 5.02 | 6.51 | 2.74\*\*\* |
| DistrictSAS Nagar | 1.21 |  |  |
| DistrictSatara | 0.56 |  |  |
| DistrictSatna | 1.15 | 3.76 | 0.66 |
| DistrictSawai Madhopur | 1.12 |  |  |
| DistrictSehore | 0.40 | 3.20 | 0.68 |
| DistrictSenapati | 0.0000 | 0.00 | 0.42\*\* |
| DistrictSeoni | 1.61 | 2.17 | 0.55\*\* |
| DistrictSERCHHIP | 0.21 | 0.72 | 0.23\*\*\* |
| DistrictShahdol | 1.31 | 3.17 | 1.29 |
| DistrictShahid Bhagat Singh Nagar | 0.56 |  |  |
| DistrictShahjahanpur | 0.78 | 1.93 | 0.87 |
| DistrictShajapur | 2.05 | 1.41 | 0.56\* |
| DistrictSheikhpura | 4.20 | 10.67 | 2.31\*\*\* |
| DistrictSheohar | 2.98 | 5.66 | 4.16\*\*\* |
| DistrictSheopur | 3.11 | 4.60 | 1.05 |
| DistrictShimla | 0.12 |  |  |
| DistrictShimoga | 0.91 | 4.17 | 0.85 |
| DistrictShivpuri | 4.14 | 4.97 | 0.79 |
| DistrictShrawasti | 5.51 | 4.52 | 2.03\*\* |
| DistrictSibsagar | 0.38 | 0.02 | 0.91 |
| DistrictSiddharthnagar | 1.42 | 3.40 | 1.15 |
| DistrictSidhi | 2.25 | 3.03 | 0.53\*\* |
| DistrictSikar | 0.26 |  |  |
| DistrictSindhudurg | 0.45 |  |  |
| DistrictSirmaur | 0.47 |  |  |
| DistrictSirohi | 0.91 |  |  |
| DistrictSirsa | 1.03 |  |  |
| DistrictSitamarhi | 2.57 | 18.23 | 2.35\*\*\* |
| DistrictSitapur | 2.65 | 2.30 | 0.81 |
| DistrictSivaganga | 0.62 |  |  |
| DistrictSiwan | 8.22 | 16.87 | 3.86\*\*\* |
| DistrictSolan | 0.03 | 0.36 | 0.77 |
| DistrictSolapur | 0.48 |  |  |
| DistrictSonapur | 0.53 | 0.84 | 1.60 |
| DistrictSonbhadra | 1.15 | 3.28 | 0.52\*\* |
| DistrictSonipath | 1.95 | 0.55 | 1.45 |
| DistrictSonitpur | 0.83 | 0.02 | 1.40 |
| DistrictSouth | 0.11 | 1.85 | 0.60 |
| DistrictSouth 24 Parganas | 0.63 |  |  |
| DistrictSouth Andamana | 0.14 |  |  |
| DistrictSouth Garo Hills | 0.19 | 1.24 | 0.68 |
| DistrictSouth Goa | 0.36 | 1.16 | 1.00 |
| DistrictSouth Tripura | 0.39 | 0.91 | 0.53 |
| DistrictSri Potti Sriramulu Nellore | 0.47 | 2.46 | 1.77 |
| DistrictSrikakulam | 1.71 |  |  |
| DistrictSultanpur | 1.81 | 4.50 | 1.13 |
| DistrictSundargarh | 0.58 | 3.51 | 1.32 |
| DistrictSupaul | 2.57 | 18.18 | 2.84\*\*\* |
| DistrictSurguja | 0.78 | 2.12 | 0.56\*\* |
| DistrictTamenglong | 0.11 | 0.96 | 0.15\*\*\* |
| DistrictTaran Taran | 1.15 |  |  |
| DistrictTawang | 0.14 | 0.09 | 0.52\* |
| DistrictTehri Garhwal | 0.35 | 2.51 | 0.85 |
| DistrictThane | 0.93 |  |  |
| DistrictThanjavur | 0.52 |  |  |
| DistrictTheni | 0.59 |  |  |
| DistrictThiruvallur | 1.13 |  |  |
| DistrictThiruvananthapuram | 0.00 | 5.33 | 4.13\*\*\* |
| DistrictThiruvarur | 0.34 |  |  |
| DistrictThoothukkudi | 0.53 | 0.00 |  |
| DistrictThoubal | 0.08 | 0.33 | 0.39\*\*\* |
| DistrictThrissur | 1.81 | 0.00 | 2.90\*\*\* |
| DistrictTikamgarh | 2.48 | 3.77 | 0.43\*\*\* |
| DistrictTinsukia | 0.95 | 0.00 | 1.62 |
| DistrictTirap | 0.16 | 0.19 | 0.87 |
| DistrictTiruchirappalli | 1.33 |  |  |
| DistrictTirunelveli | 0.39 |  |  |
| DistrictTiruppur | 0.67 |  |  |
| DistrictTiruvannamalai | 1.09 |  |  |
| DistrictTonk | 0.82 |  |  |
| DistrictTuensang | 0.58 |  |  |
| DistrictTumkur | 0.42 | 1.29 | 0.79 |
| DistrictUdaipur | 1.61 |  |  |
| DistrictUdham Singh Nagar | 0.86 | 1.20 | 0.50\*\*\* |
| DistrictUdupi | 0.02 | 1.64 | 1.47 |
| DistrictUjjain | 0.30 | 3.61 | 0.95 |
| DistrictUkhrul | 0.22 | 0.79 | 0.14\*\*\* |
| DistrictUmaria | 2.78 | 2.92 | 0.70 |
| DistrictUna | 0.29 | 0.14 | 1.04 |
| DistrictUnnao | 1.40 | 1.55 | 1.23 |
| DistrictUpper Siang | 0.09 | 0.42 | 0.22\*\*\* |
| DistrictUpper Subansiri | 0.10 | 0.05 | 0.19\*\*\* |
| DistrictUttar Dinajpur | 2.75 |  | 6.93\*\*\* |
| DistrictUttara Kannada | 0.71 | 2.08 | 0.65\* |
| DistrictUttarkashi | 0.09 | 2.02 | 0.86 |
| DistrictVaishali | 3.63 | 10.69 | 2.91\*\*\* |
| DistrictVaranasi | 1.26 | 2.69 | 0.85 |
| DistrictVellore | 1.53 |  |  |
| DistrictVidisha | 1.23 | 17.04 | 0.43\*\*\* |
| DistrictViluppuram | 1.19 |  |  |
| DistrictVirudhunagar | 0.82 |  |  |
| DistrictVishakapatnam | 0.95 | 3.16 | 1.36 |
| DistrictVizianagaram | 1.40 |  |  |
| DistrictWardha | 0.28 |  |  |
| DistrictWarngal | 1.66 |  |  |
| DistrictWashim | 0.49 |  |  |
| DistrictWayanand | 0.03 | 0.54 | 1.21 |
| DistrictWest | 0.45 | 2.42 | 0.74 |
| DistrictWest Garo Hills | 0.29 | 1.41 | 0.66 |
| DistrictWest Godavari | 0.68 | 2.59 | 1.17 |
| DistrictWest Kameng | 0.25 | 1.07 | 0.57 |
| DistrictWest Khasi Hills | 0.28 | 0.72 | 0.83 |
| DistrictWest Nimar | 2.14 | 1.89 | 0.59\*\* |
| DistrictWest Siang | 0.07 | 0.51 | 0.45\*\*\* |
| DistrictWest Tripura | 0.22 | 1.93 | 0.60 |
| DistrictWokha | 0.03 |  |  |
| DistrictY.S.R. | 0.81 | 0.00 | 0.01\*\*\* |
| DistrictYadgir | 1.36 | 2.17 | 0.93 |
| DistrictYamunanagar | 0.54 | 0.80 | 1.34 |
| DistrictYavatmal | 1.21 |  |  |
| DistrictZunheboto | 0.22 |  |  |
| ElectricityIrregular Electricity:Generator | 0.94 | 0.97 | 0.92\* |
| ElectricityNo Electricity:Generator | 1.87 | 0.87 | 0.93 |
| ElectricityIrregular Electricity:`24x7` | 1.04 | 0.88 | 0.94 |
| ElectricityNo Electricity:`24x7` | 1.55 | 0.71 | 0.92 |
| ElectricityIrregular Electricity:MO\_Total | 1.01 | 1.04 | 0.99 |
| ElectricityNo Electricity:MO\_Total | 0.82 | 1.01 | 1.08\*\* |
| ElectricityIrregular Electricity:LMO\_Total | 1.01 | 1.06 | 0.96 |
| ElectricityNo Electricity:LMO\_Total | 0.99 | 0.45 | 0.85\* |
| ElectricityIrregular Electricity:Nurse\_Total | 1.01 | 0.97 | 0.99 |
| ElectricityNo Electricity:Nurse\_Total | 0.98 | 0.82 | 0.97 |
| ElectricityIrregular Electricity:LHV\_Total | 0.99 | 1.10 | 1.01 |
| ElectricityNo Electricity:LHV\_Total | 1.47 | 1.13 | 0.90 |
| ElectricityIrregular Electricity:ANM\_Total | 0.98 | 1.01 | 1.01 |
| ElectricityNo Electricity:ANM\_Total | 1.23 | 0.98 | 1.08\*\*\* |
| ElectricityIrregular Electricity:Pharma\_Total | 1.09 | 1.02 | 1.04 |
| ElectricityNo Electricity:Pharma\_Total | 0.95 | 0.69 | 1.27\*\*\* |
| ElectricityIrregular Electricity:MO\_Residing | 1.10 | 0.97 | 1.12\*\* |
| ElectricityNo Electricity:MO\_Residing | 0.99 | 0.75 | 0.96 |
| ElectricityIrregular Electricity:Autoclave | 1.07 | 1.13 | 1.02 |
| ElectricityNo Electricity:Autoclave | 0.87 | 1.08 | 1.02 |
| ElectricityIrregular Electricity:RadiantWarmer | 1.09 |  |  |
| ElectricityNo Electricity:RadiantWarmer | 1.23 |  |  |
| ElectricityIrregular Electricity:DF\_Large |  | 0.95 | 0.97 |
| ElectricityNo Electricity:DF\_Large |  | 1.64 | 1.26 |
| ElectricityIrregular Electricity:ILR\_Large |  | 0.97 | 1.05 |
| ElectricityNo Electricity:ILR\_Large |  | 0.44 | 1.03 |
| ElectricityIrregular Electricity:Centrifuge |  | 1.17 | 0.95 |
| ElectricityNo Electricity:Centrifuge |  | 2.02 | 1.02 |
| Constant | 7.29 | 4.36 | 307.85\*\*\* |
|  | | | |
| Observations | 7,981 | 4,684 | 4,942 |
| Log Likelihood | -22,062.39 | -14,343.52 | -36,420.50 |
| theta |  |  | 2.37\*\*\* (0.05) |
| Akaike Inf. Crit. |  |  | 73,733.01 |
|  | | | |
| *Note:* | \*p<0.1; \*\*p<0.05; \*\*\*p<0.01 | | |
