# Supplementary material for: Reduced health services at under-electrified primary healthcare facilities: Evidence from India
Source: PLoS One. 2021 Jun 4;16(6):e0252705. doi: 10.1371/journal.pone.0252705 (PMC8177862; doi:10.1371/journal.pone.0252705)
Supplement: S1 Replication materials — (ZIP) [file pone.0252705.s002.zip › Replication material - PLOS ONE Review - Revised/Results/All_Models_DLHS3.html]

**DLHS-3 only**

|  | | | |
|  | *Dependent variable:* | | |
|  |  | | |
|  | Deliveries | IPD | OPD |
|  | *zero-inflated* | *zero-inflated* | *negative* |
|  | *count data* | *count data* | *binomial* |
|  | (1) | (2) | (3) |
|  | | | |
| ElectricityIrregular Electricity | 0.76 | 0.47\*\*\* | 0.88 |
| ElectricityNo Electricity | 0.33\*\*\* | 0.49\*\* | 0.71\*\*\* |
| Generator | 0.94 | 1.13 | 0.99 |
| Urban | 0.87\* | 0.83\*\* | 0.89\*\* |
| Population10000 | 1.07\*\*\* | 1.03\*\*\* | 1.04\*\*\* |
| `24x7` | 1.22\*\*\* | 1.38\*\*\* | 1.08\* |
| Beds | 1.01\*\*\* | 1.04\*\*\* | 1.01\*\*\* |
| MO | 1.06 | 0.78\* | 1.00 |
| LMO | 1.17\*\* | 1.29\*\*\* | 1.09\*\* |
| Nurse | 1.34\*\*\* | 1.18\*\* | 1.15\*\*\* |
| LHV | 1.17\*\* | 1.03 | 1.08\* |
| ANM | 1.09 | 0.95 | 1.10\* |
| Pharma | 0.98 | 1.18\*\* | 1.13\*\* |
| MO\_Residing | 1.01 | 1.26\*\*\* | 1.05 |
| Autoclave | 1.19\*\* | 0.85\* | 1.18\*\*\* |
| RadiantWarmer | 1.24\*\* |  |  |
| DF\_Large |  | 0.96 | 1.10\* |
| ILR\_Large |  | 1.11 | 1.01 |
| Centrifuge |  | 1.34\*\*\* | 1.13\*\*\* |
| Govt\_Building | 0.89\* | 1.36\*\*\* | 0.94\* |
| Condition | 0.88\*\*\* | 1.01 | 0.96 |
| Water | 1.07\* | 1.03 | 1.01 |
| Toilet | 1.10\* | 0.94 | 1.18\*\*\* |
| StateAndra Pradesh | 22.76\*\*\* | 1.36 | 3.03\*\*\* |
| StateArunachal Pradesh | 1.31 | 0.82 | 0.51\*\*\* |
| StateAssam | 3.54\*\*\* | 3.08\*\*\* | 1.23 |
| StateBihar | 9.41\*\*\* | 4.22\*\*\* | 1.08 |
| StateChhattisgarh | 3.06\*\*\* | 0.51\* | 0.60\*\* |
| StateGoa | 0.32\*\* | 0.83 | 0.75 |
| StateHaryana | 3.75\*\*\* | 0.43\*\* | 1.05 |
| StateHimachal Pradesh | 1.30 | 0.82 | 0.91 |
| StateJharkhand | 2.06\* | 0.99 | 1.02 |
| StateKarnataka | 3.48\*\*\* | 0.89 | 0.90 |
| StateKerala | 0.06\*\*\* | 0.44\*\* | 1.73\*\* |
| StateMadhya Pradesh | 9.03\*\*\* | 1.52 | 0.51\*\*\* |
| StateMaharashtra | 4.45\*\*\* | 0.82 | 0.80 |
| StateManipur | 1.57 | 1.15 | 0.41\*\*\* |
| StateMeghalaya | 1.45 | 0.58 | 0.63\* |
| StateMizoram | 1.54 | 0.66 | 0.19\*\*\* |
| StateOdisha | 2.68\*\*\* | 1.40 | 0.82 |
| StatePuducherry | 1.07 | 0.91 | 3.41\*\*\* |
| StatePunjab | 3.74\*\*\* | 0.73 | 0.87 |
| StateRajasthan | 3.55\*\*\* | 0.94 | 0.66\* |
| StateSikkim | 1.20 | 0.69 | 0.74 |
| StateTamil Nadu | 3.82\*\*\* | 1.28 | 3.05\*\*\* |
| StateTripura | 2.23\*\* | 4.88\*\*\* | 1.08 |
| StateUttar Pradesh | 2.40\*\* | 0.68 | 0.94 |
| StateUttrakhand | 1.95\* | 0.56 | 0.73 |
| StateWest Bengal | 1.81 | 0.92 | 3.82\*\*\* |
| ElectricityIrregular Electricity:Generator | 1.27\*\*\* | 1.10 | 1.32\*\*\* |
| ElectricityNo Electricity:Generator | 1.24 | 1.61\*\* | 1.35\*\*\* |
| ElectricityIrregular Electricity:`24x7` | 0.88 | 0.85\* | 0.97 |
| ElectricityNo Electricity:`24x7` | 0.86 | 0.93 | 1.23\*\*\* |
| ElectricityIrregular Electricity:MO | 1.37\*\* | 1.61\*\*\* | 1.09 |
| ElectricityNo Electricity:MO | 1.58\*\* | 1.40 | 1.12 |
| ElectricityIrregular Electricity:LMO | 0.90 | 0.98 | 1.00 |
| ElectricityNo Electricity:LMO | 0.73\* | 0.74 | 0.77\*\* |
| ElectricityIrregular Electricity:Nurse | 0.86\*\* | 0.89 | 0.88\*\*\* |
| ElectricityNo Electricity:Nurse | 1.65\*\*\* | 0.82 | 0.93 |
| ElectricityIrregular Electricity:LHV | 0.93 | 1.09 | 0.93 |
| ElectricityNo Electricity:LHV | 0.94 | 0.73\*\* | 0.99 |
| ElectricityIrregular Electricity:ANM | 1.27\*\* | 1.25\* | 1.01 |
| ElectricityNo Electricity:ANM | 2.01\*\*\* | 1.98\*\*\* | 0.97 |
| ElectricityIrregular Electricity:Pharma | 0.95 | 0.92 | 0.98 |
| ElectricityNo Electricity:Pharma | 1.22 | 0.77 | 1.10 |
| ElectricityIrregular Electricity:MO\_Residing | 1.22\*\* | 1.08 | 1.11\*\* |
| ElectricityNo Electricity:MO\_Residing | 1.07 | 0.77 | 0.92 |
| ElectricityIrregular Electricity:Autoclave | 0.80\*\* | 1.14 | 1.01 |
| ElectricityNo Electricity:Autoclave | 0.87 | 0.91 | 0.94 |
| ElectricityIrregular Electricity:RadiantWarmer | 0.94 |  |  |
| ElectricityNo Electricity:RadiantWarmer | 1.04 |  |  |
| ElectricityIrregular Electricity:DF\_Large |  | 1.05 | 0.91 |
| ElectricityNo Electricity:DF\_Large |  | 0.85 | 0.97 |
| ElectricityIrregular Electricity:ILR\_Large |  | 0.94 | 1.02 |
| ElectricityNo Electricity:ILR\_Large |  | 0.81 | 1.14 |
| ElectricityIrregular Electricity:Centrifuge |  | 0.99 | 1.00 |
| ElectricityNo Electricity:Centrifuge |  | 1.22 | 0.82 |
| Constant | 1.91 | 13.54\*\*\* | 416.41\*\*\* |
|  | | | |
| Observations | 7,096 | 7,023 | 7,214 |
| Log Likelihood | -23,674.83 | -22,089.53 | -55,588.73 |
| theta |  |  | 1.34\*\*\* (0.02) |
| Akaike Inf. Crit. |  |  | 111,327.50 |
|  | | | |
| *Note:* | \*p<0.1; \*\*p<0.05; \*\*\*p<0.01 | | |
