# Supplementary material for: Reduced health services at under-electrified primary healthcare facilities: Evidence from India
Source: PLoS One. 2021 Jun 4;16(6):e0252705. doi: 10.1371/journal.pone.0252705 (PMC8177862; doi:10.1371/journal.pone.0252705)
Supplement: S1 Replication materials — (ZIP) [file pone.0252705.s002.zip › Replication material - PLOS ONE Review - Revised/Results/All_Models_Electricity_2cat.html]

**All Models - Electricity - 2 categories**

|  | | | |
|  | *Dependent variable:* | | |
|  |  | | |
|  | Deliveries | IPD | OPD |
|  | *zero-inflated* | *zero-inflated* | *negative* |
|  | *count data* | *count data* | *binomial* |
|  | (1) | (2) | (3) |
|  | | | |
| Electricity\_2catIrregular Electricity | 0.84\* | 1.10 | 0.84\*\*\* |
| Generator | 1.02 | 1.22\*\* | 1.25\*\*\* |
| Urban | 0.79\*\*\* | 0.80\*\*\* | 0.97 |
| Population10000 | 1.05\*\*\* | 1.02\*\*\* | 1.02\*\*\* |
| `24x7` | 1.45\*\*\* | 1.36\*\*\* | 1.08\* |
| Beds | 1.01\*\*\* | 1.05\*\*\* | 1.00 |
| MO\_Total | 1.05\*\* | 1.11\*\*\* | 1.10\*\*\* |
| LMO\_Total | 0.99 | 0.92 | 0.99 |
| Nurse\_Total | 1.00 | 1.07\*\* | 1.06\*\*\* |
| LHV\_Total | 1.07\*\*\* | 0.98 | 1.02 |
| ANM\_Total | 1.04\*\*\* | 1.03 | 1.02\*\* |
| Pharma\_Total | 0.96 | 1.02 | 1.05\* |
| MO\_Residing | 1.11\*\* | 1.32\*\*\* | 1.08\* |
| Autoclave | 1.07 | 1.19\*\* | 1.08\* |
| RadiantWarmer | 1.28\*\*\* |  |  |
| DF\_Large |  | 0.97 | 1.07 |
| ILR\_Large |  | 1.16 | 1.02 |
| Centrifuge |  | 1.18\*\* | 1.19\*\*\* |
| Govt\_Building | 0.98 | 1.15 | 1.03 |
| Condition | 0.96 | 0.95 | 1.01 |
| Water | 1.11\*\*\* | 0.95 | 1.06\*\* |
| Toilet | 0.79\*\*\* | 0.86\*\* | 1.16\*\*\* |
| StateAndra Pradesh | 4.96\*\*\* |  |  |
| StateArunachal Pradesh | 0.74 | 0.24\*\*\* | 0.28\*\*\* |
| StateAssam | 5.95\*\*\* | 0.31\*\*\* | 0.93 |
| StateBihar | 21.54\*\*\* | 5.25\*\*\* | 1.80\*\*\* |
| StateChhattisgarh | 3.36\*\*\* | 0.69\* | 0.46\*\*\* |
| StateGoa | 4.77\*\*\* | 0.53 | 0.68\* |
| StateHaryana | 5.86\*\*\* | 0.96 | 1.05 |
| StateHimachal Pradesh | 1.94\* | 0.20\*\*\* | 0.67\*\* |
| StateJharkhand | 7.13\*\*\* | 0.72 | 0.56\*\*\* |
| StateKarnataka | 3.70\*\*\* | 0.99 | 0.52\*\*\* |
| StateKerala | 6.75\*\*\* | 2.68\*\*\* | 0.86 |
| StateMadhya Pradesh | 8.20\*\*\* | 0.87 | 0.42\*\*\* |
| StateMaharashtra | 3.36\*\*\* | 1.38 | 0.08\*\*\* |
| StateManipur | 1.61 | 1.04 | 0.18\*\*\* |
| StateMeghalaya | 2.54\*\*\* | 0.67\* | 0.49\*\*\* |
| StateMizoram | 1.51 | 0.45\*\*\* | 0.22\*\*\* |
| StateNagaland | 0.89 | 0.21\*\* | 0.19\*\*\* |
| StateOdisha | 5.08\*\*\* | 1.09 | 1.19 |
| StatePuducherry | 13.69\*\*\* |  |  |
| StatePunjab | 4.62\*\*\* | 0.0000 | 0.23\*\*\* |
| StateRajasthan | 3.81\*\*\* |  |  |
| StateSikkim | 1.29 | 0.55\*\* | 0.42\*\*\* |
| StateTamil Nadu | 4.20\*\*\* | 10.09\*\*\* | 3.99\*\*\* |
| StateTelangana | 3.02\*\*\* | 1.44 | 1.32\* |
| StateTripura | 2.78\*\*\* | 1.19 | 0.49\*\*\* |
| StateUttar Pradesh | 7.15\*\*\* | 1.06 | 0.78\*\* |
| StateUttrakhand | 2.50\*\*\* | 0.58\*\* | 0.51\*\*\* |
| StateWest Bengal | 2.78\*\*\* | 0.56 | 2.65\*\*\* |
| Electricity\_2catIrregular Electricity:Generator | 0.98 | 1.04 | 0.92\* |
| Electricity\_2catIrregular Electricity:`24x7` | 1.07 | 0.92 | 0.99 |
| Electricity\_2catIrregular Electricity:MO\_Total | 0.96 | 0.98 | 1.03 |
| Electricity\_2catIrregular Electricity:LMO\_Total | 0.96 | 1.10 | 1.02 |
| Electricity\_2catIrregular Electricity:Nurse\_Total | 1.07\*\*\* | 1.02 | 0.98 |
| Electricity\_2catIrregular Electricity:LHV\_Total | 0.99 | 1.21\*\*\* | 1.02 |
| Electricity\_2catIrregular Electricity:ANM\_Total | 0.98 | 0.95\*\* | 1.02 |
| Electricity\_2catIrregular Electricity:Pharma\_Total | 1.11\*\* | 1.00 | 1.09\*\* |
| Electricity\_2catIrregular Electricity:MO\_Residing | 1.23\*\*\* | 1.03 | 1.07 |
| Electricity\_2catIrregular Electricity:Autoclave | 1.04 | 0.83\* | 0.99 |
| Electricity\_2catIrregular Electricity:RadiantWarmer | 1.12 |  |  |
| Electricity\_2catIrregular Electricity:DF\_Large |  | 1.19 | 1.02 |
| Electricity\_2catIrregular Electricity:ILR\_Large |  | 0.93 | 1.02 |
| Electricity\_2catIrregular Electricity:Centrifuge |  | 1.08 | 0.97 |
| Constant | 1.66 | 12.74\*\*\* | 481.38\*\*\* |
|  | | | |
| Observations | 7,805 | 4,540 | 4,782 |
| Log Likelihood | -22,454.49 | -14,409.36 | -35,852.58 |
| theta |  |  | 1.86\*\*\* (0.04) |
| Akaike Inf. Crit. |  |  | 71,825.15 |
|  | | | |
| *Note:* | \*p<0.1; \*\*p<0.05; \*\*\*p<0.01 | | |
