# Supplementary material for: Reduced health services at under-electrified primary healthcare facilities: Evidence from India
Source: PLoS One. 2021 Jun 4;16(6):e0252705. doi: 10.1371/journal.pone.0252705 (PMC8177862; doi:10.1371/journal.pone.0252705)
Supplement: S1 Replication materials — (ZIP) [file pone.0252705.s002.zip › Replication material - PLOS ONE Review - Revised/Results/All_Models_Electricity_5cat.html]

**All Models - Electricity - 5 categories**

|  | | | |
|  | *Dependent variable:* | | |
|  |  | | |
|  | Deliveries | IPD | OPD |
|  | *zero-inflated* | *zero-inflated* | *negative* |
|  | *count data* | *count data* | *binomial* |
|  | (1) | (2) | (3) |
|  | | | |
| Electricity\_5catOccasional Powercut | 0.94 | 1.07 | 1.05 |
| Electricity\_5catSummer Powercut | 0.64 | 0.16 | 0.99 |
| Electricity\_5catRegular Powercut | 1.02 | 1.35 | 0.82\*\*\* |
| Electricity\_5catNo Electricity | 0.36\*\*\* | 1.77 | 0.62\*\*\* |
| Generator | 1.03 | 1.20 | 1.25\*\*\* |
| Urban | 0.79\*\*\* | 0.82 | 0.96 |
| Population10000 | 1.05\*\*\* | 1.02 | 1.02\*\*\* |
| `24x7` | 1.45\*\*\* | 1.35 | 1.09\*\* |
| Beds | 1.01\*\* | 1.05 | 1.00 |
| MO\_Total | 1.05\*\* | 1.10 | 1.10\*\*\* |
| LMO\_Total | 1.00 | 0.92 | 0.98 |
| Nurse\_Total | 1.01 | 1.08 | 1.06\*\*\* |
| LHV\_Total | 1.07\*\*\* | 0.98 | 1.02 |
| ANM\_Total | 1.04\*\*\* | 1.03 | 1.02\* |
| Pharma\_Total | 0.97 | 1.03 | 1.06\* |
| MO\_Residing | 1.12\*\* | 1.32 | 1.08\*\* |
| Autoclave | 1.08 | 1.19 | 1.08\*\* |
| RadiantWarmer | 1.28\*\*\* |  |  |
| DF\_Large |  | 0.96 | 1.07 |
| Electricity\_2catIrregular Electricity |  | 0.89 |  |
| ILR\_Large |  | 1.16 | 1.02 |
| Centrifuge |  | 1.20 | 1.19\*\*\* |
| Govt\_Building | 0.99 | 1.17 | 1.02 |
| Condition | 0.95 | 0.95 | 1.01 |
| Water | 1.10\*\* | 0.95 | 1.06\*\* |
| Toilet | 0.76\*\*\* | 0.87 | 1.12\*\*\* |
| StateAndra Pradesh | 5.10\*\*\* |  |  |
| StateArunachal Pradesh | 0.72 | 0.25 | 0.28\*\*\* |
| StateAssam | 6.15\*\*\* | 0.28 | 0.92 |
| StateBihar | 23.37\*\*\* | 5.44 | 1.86\*\*\* |
| StateChhattisgarh | 3.51\*\*\* | 0.72 | 0.44\*\*\* |
| StateGoa | 4.99\*\*\* | 0.50 | 0.70 |
| StateHaryana | 5.88\*\*\* | 0.92 | 1.02 |
| StateHimachal Pradesh | 2.00\*\* | 0.20 | 0.65\*\* |
| StateJharkhand | 8.43\*\*\* | 0.79 | 0.59\*\*\* |
| StateKarnataka | 3.75\*\*\* | 0.94 | 0.51\*\*\* |
| StateKerala | 6.75\*\*\* | 2.62 | 0.85 |
| StateMadhya Pradesh | 8.48\*\*\* | 0.89 | 0.41\*\*\* |
| StateMaharashtra | 3.47\*\*\* | 1.38 | 0.08\*\*\* |
| StateManipur | 1.73 | 0.76 | 0.18\*\*\* |
| StateMeghalaya | 2.43\*\*\* | 0.60 | 0.49\*\*\* |
| StateMizoram | 1.59 | 0.48 | 0.21\*\*\* |
| StateNagaland | 0.96 | 0.25 | 0.17\*\*\* |
| StateOdisha | 5.09\*\*\* | 1.10 | 1.18 |
| StatePuducherry | 13.31\*\*\* |  |  |
| StatePunjab | 4.77\*\*\* | 0.0000 | 0.22\*\*\* |
| StateRajasthan | 3.88\*\*\* |  |  |
| StateSikkim | 1.46 | 0.55 | 0.42\*\*\* |
| StateTamil Nadu | 4.32\*\*\* | 10.35 | 3.87\*\* |
| StateTelangana | 3.21\*\*\* | 1.34 | 1.34\* |
| StateTripura | 2.92\*\*\* | 1.18 | 0.49\*\*\* |
| StateUttar Pradesh | 7.55\*\*\* | 1.09 | 0.76\*\* |
| StateUttrakhand | 2.55\*\*\* | 0.65 | 0.50\*\*\* |
| StateWest Bengal | 2.86\*\*\* | 0.55 | 2.62\*\*\* |
| Electricity\_5catOccasional Powercut:Generator | 0.96 | 0.92 | 0.89\* |
| Electricity\_5catSummer Powercut:Generator | 0.90 | 2.84 | 1.11 |
| Electricity\_5catRegular Powercut:Generator | 0.91 | 1.12 | 0.90\* |
| Electricity\_5catNo Electricity:Generator | 2.12\*\*\* | 1.80 | 0.89 |
| Electricity\_5catOccasional Powercut:`24x7` | 0.91 | 1.01 | 0.95 |
| Electricity\_5catSummer Powercut:`24x7` | 1.80\* | 3.53 | 1.07 |
| Electricity\_5catRegular Powercut:`24x7` | 1.17\* | 0.95 | 1.05 |
| Electricity\_5catNo Electricity:`24x7` | 1.46\*\* | 0.58 | 0.88 |
| Electricity\_5catOccasional Powercut:MO\_Total | 1.02 | 1.10 | 1.01 |
| Electricity\_5catSummer Powercut:MO\_Total | 1.00 | 1.11 | 0.90 |
| Electricity\_5catRegular Powercut:MO\_Total | 0.92\*\* | 0.95 | 1.04 |
| Electricity\_5catNo Electricity:MO\_Total | 0.77\*\*\* | 0.97 | 1.10\*\* |
| Electricity\_5catOccasional Powercut:LMO\_Total | 0.96 | 1.16 | 0.99 |
| Electricity\_5catSummer Powercut:LMO\_Total | 0.74\* | 0.64 | 0.84 |
| Electricity\_5catRegular Powercut:LMO\_Total | 0.99 | 1.13 | 1.08 |
| Electricity\_5catNo Electricity:LMO\_Total | 0.70 | 0.51 | 0.96 |
| Electricity\_5catOccasional Powercut:Nurse\_Total | 1.04 | 0.97 | 0.97 |
| Electricity\_5catSummer Powercut:Nurse\_Total | 1.11 | 0.89 | 0.98 |
| Electricity\_5catRegular Powercut:Nurse\_Total | 1.08\*\*\* | 1.04 | 0.98 |
| Electricity\_5catNo Electricity:Nurse\_Total | 1.12 | 0.92 | 0.98 |
| Electricity\_5catOccasional Powercut:LHV\_Total | 0.96 | 1.09 | 1.05 |
| Electricity\_5catSummer Powercut:LHV\_Total | 1.03 | 0.73 | 0.76 |
| Electricity\_5catRegular Powercut:LHV\_Total | 0.96 | 1.21 | 1.01 |
| Electricity\_5catNo Electricity:LHV\_Total | 1.60\*\*\* | 1.36 | 0.97 |
| Electricity\_5catOccasional Powercut:ANM\_Total | 0.99 | 0.99 | 1.01 |
| Electricity\_5catSummer Powercut:ANM\_Total | 1.04 | 1.08 | 1.06 |
| Electricity\_5catRegular Powercut:ANM\_Total | 0.97\*\* | 0.94 | 1.01 |
| Electricity\_5catNo Electricity:ANM\_Total | 1.21\*\*\* | 0.88 | 1.09\*\*\* |
| Electricity\_5catOccasional Powercut:Pharma\_Total | 1.10\* | 1.06 | 1.00 |
| Electricity\_5catSummer Powercut:Pharma\_Total | 0.99 | 0.73 | 1.11 |
| Electricity\_5catRegular Powercut:Pharma\_Total | 1.12\* | 1.01 | 1.08 |
| Electricity\_5catNo Electricity:Pharma\_Total | 0.93 | 0.77 | 1.27\*\*\* |
| Electricity\_5catOccasional Powercut:MO\_Residing | 1.14 | 0.98 | 1.05 |
| Electricity\_5catSummer Powercut:MO\_Residing | 1.11 | 1.02 | 1.64\* |
| Electricity\_5catRegular Powercut:MO\_Residing | 1.29\*\*\* | 1.07 | 1.05 |
| Electricity\_5catNo Electricity:MO\_Residing | 1.03 | 0.80 | 0.99 |
| Electricity\_5catOccasional Powercut:Autoclave | 1.02 | 0.96 | 0.96 |
| Electricity\_5catSummer Powercut:Autoclave | 1.01 | 1.08 | 0.81 |
| Electricity\_5catRegular Powercut:Autoclave | 1.00 | 0.73 | 0.98 |
| Electricity\_5catNo Electricity:Autoclave | 0.98 | 0.70 | 1.05 |
| Electricity\_5catOccasional Powercut:RadiantWarmer | 1.27\*\*\* |  |  |
| Electricity\_5catSummer Powercut:RadiantWarmer | 0.63\* |  |  |
| Electricity\_5catRegular Powercut:RadiantWarmer | 0.98 |  |  |
| Electricity\_5catNo Electricity:RadiantWarmer | 1.52 |  |  |
| Electricity\_5catOccasional Powercut:DF\_Large |  | 0.96 | 1.07 |
| Electricity\_5catSummer Powercut:DF\_Large |  | 0.66 | 0.61 |
| Electricity\_5catRegular Powercut:DF\_Large |  | 1.43 | 0.95 |
| Electricity\_5catNo Electricity:DF\_Large |  | 1.30 | 1.50 |
| Electricity\_2catIrregular Electricity:ILR\_Large |  | 0.92 |  |
| Electricity\_5catOccasional Powercut:ILR\_Large |  |  | 0.95 |
| Electricity\_5catSummer Powercut:ILR\_Large |  |  | 1.90\* |
| Electricity\_5catRegular Powercut:ILR\_Large |  |  | 1.10 |
| Electricity\_5catNo Electricity:ILR\_Large |  |  | 0.83 |
| Electricity\_5catOccasional Powercut:Centrifuge |  | 0.93 | 0.98 |
| Electricity\_5catSummer Powercut:Centrifuge |  | 2.06 | 0.65\* |
| Electricity\_5catRegular Powercut:Centrifuge |  | 1.12 | 0.98 |
| Electricity\_5catNo Electricity:Centrifuge |  | 1.28 | 0.91 |
| Constant | 1.68 | 11.73 | 520.26\*\*\* |
|  | | | |
| Observations | 7,805 | 4,540 | 4,782 |
| Log Likelihood | -22,397.97 | -14,371.40 | -35,818.03 |
| theta |  |  | 1.88\*\*\* (0.04) |
| Akaike Inf. Crit. |  |  | 71,840.07 |
|  | | | |
| *Note:* | \*p<0.1; \*\*p<0.05; \*\*\*p<0.01 | | |
