# Supplementary material for: Reduced health services at under-electrified primary healthcare facilities: Evidence from India
Source: PLoS One. 2021 Jun 4;16(6):e0252705. doi: 10.1371/journal.pone.0252705 (PMC8177862; doi:10.1371/journal.pone.0252705)
Supplement: S1 Replication materials — (ZIP) [file pone.0252705.s002.zip › Replication material - PLOS ONE Review - Revised/Results/All_Models_Linear.html]

**All Models - Linear**

|  | | | |
|  | *Dependent variable:* | | |
|  |  | | |
|  | Deliveries | IPD | OPD |
|  | *OLS* | *OLS* | *OLS* |
|  | (1) | (2) | (3) |
|  | | | |
| ElectricityIrregular Electricity | 2.68 | 20.49\*\*\* | 16.56 |
| ElectricityNo Electricity | -15.51\*\*\* | 16.75\*\* | -359.97\*\*\* |
| Generator | 1.84 | 7.71\*\* | 185.30\*\*\* |
| Urban | -1.02 | -7.50\* | -4.98 |
| Population10000 | 1.78\*\*\* | 2.75\*\*\* | 39.42\*\*\* |
| `24x7` | 11.06\*\*\* | 20.69\*\*\* | 179.40\*\*\* |
| Beds | 0.17\* | 3.51\*\*\* | -9.80\*\* |
| MO\_Total | 7.65\*\*\* | 15.47\*\*\* | 280.89\*\*\* |
| LMO\_Total | -2.38\*\*\* | 7.56\*\* | -24.97 |
| Nurse\_Total | -2.21\*\*\* | -3.17\*\* | 48.18\*\*\* |
| LHV\_Total | 0.47 | 4.32\* | -73.87\*\*\* |
| ANM\_Total | 0.59\*\*\* | 1.01 | 5.05 |
| Pharma\_Total | -2.27\*\*\* | 9.04\*\*\* | -33.63 |
| MO\_Residing | 1.48 | 9.03\*\* | -2.82 |
| Autoclave | 4.98\*\*\* | 5.21 | 119.52\*\*\* |
| RadiantWarmer | 12.51\*\*\* |  |  |
| DF\_Large |  | 9.34\* | 10.22 |
| ILR\_Large |  | -12.15\*\* | 43.27 |
| Centrifuge |  | 6.87\* | 61.56 |
| Govt\_Building | 1.14 | 0.69 | 72.86 |
| Condition | -1.41 | -6.57\*\* | -27.33 |
| Water | 1.90\*\* | 0.43 | 48.20 |
| Toilet | -0.23 | 1.14 | 109.94\*\*\* |
| StateAndra Pradesh | 8.88 |  |  |
| StateArunachal Pradesh | 11.78 | 2.95 | -591.89\*\*\* |
| StateAssam | 12.31 | -8.50 | -159.67 |
| StateBihar | 74.28\*\*\* | 243.33\*\*\* | 1,394.02\*\*\* |
| StateChhattisgarh | 11.63 | 10.10 | -529.99\*\*\* |
| StateGoa | 2.59 | -34.21\* | -422.83\* |
| StateHaryana | 9.83 | 9.94 | 21.29 |
| StateHimachal Pradesh | 11.77 | 1.49 | -368.64\* |
| StateJharkhand | 25.92\*\*\* | 14.36 | -399.93\*\* |
| StateKarnataka | 9.22 | 8.30 | -524.34\*\*\* |
| StateKerala | 19.42\*\* | 87.17\*\*\* | 186.78 |
| StateMadhya Pradesh | 17.83\*\* | 7.33 | -648.72\*\*\* |
| StateMaharashtra | -3.24 | 28.70 | -685.24 |
| StateManipur | -4.04 | -17.16 | -1,106.28\*\*\* |
| StateMeghalaya | -1.83 | -27.17\*\* | -711.84\*\*\* |
| StateMizoram | 4.08 | -13.48 | -964.61\*\*\* |
| StateNagaland | 6.58 | -24.83 | -1,176.04\*\*\* |
| StateOdisha | 13.58\* | 19.98\* | 24.74 |
| StatePuducherry | 21.39\* |  |  |
| StatePunjab | 11.37 | 12.66 | -805.83\* |
| StateRajasthan | 9.48 |  |  |
| StateSikkim | -3.20 | -9.32 | -597.68\*\*\* |
| StateTamil Nadu | 3.66 | 184.46\*\*\* | 2,653.27\*\*\* |
| StateTelangana | -1.27 | 21.81 | 687.17\*\*\* |
| StateTripura | -1.33 | 34.02\* | -872.28\*\*\* |
| StateUttar Pradesh | 15.95\*\* | 16.19 | -270.31\*\* |
| StateUttrakhand | -5.04 | -11.92 | -642.89\*\*\* |
| StateWest Bengal | 9.49 | -4.70 | 1,320.90\*\*\* |
| ElectricityIrregular Electricity:Generator | -0.05 | -4.54 | -100.73\* |
| ElectricityNo Electricity:Generator | 16.09\*\*\* | 9.30 | 220.46\* |
| ElectricityIrregular Electricity:`24x7` | -4.05\*\* | -12.22\*\* | -101.77\* |
| ElectricityNo Electricity:`24x7` | 5.03 | -7.04 | 45.21 |
| ElectricityIrregular Electricity:MO\_Total | -4.84\*\*\* | -9.28\*\*\* | -108.86\*\*\* |
| ElectricityNo Electricity:MO\_Total | -10.61\*\*\* | -19.52\*\*\* | -256.81\*\*\* |
| ElectricityIrregular Electricity:LMO\_Total | 4.06\*\*\* | -8.28\* | -2.42 |
| ElectricityNo Electricity:LMO\_Total | 8.57\* | -19.54 | -8.91 |
| ElectricityIrregular Electricity:Nurse\_Total | 2.88\*\*\* | 6.77\*\*\* | -33.54 |
| ElectricityNo Electricity:Nurse\_Total | 0.07 | 10.14\* | -207.19\*\*\* |
| ElectricityIrregular Electricity:LHV\_Total | 1.40 | 0.15 | 128.17\*\*\* |
| ElectricityNo Electricity:LHV\_Total | 1.84 | -15.14\*\* | 121.53 |
| ElectricityIrregular Electricity:ANM\_Total | -0.53\* | -0.94 | 17.82 |
| ElectricityNo Electricity:ANM\_Total | -1.19 | -2.01 | 87.05\*\* |
| ElectricityIrregular Electricity:Pharma\_Total | 4.16\*\*\* | -6.85\* | 36.93 |
| ElectricityNo Electricity:Pharma\_Total | 18.90\*\*\* | 7.49 | 367.04\*\*\* |
| ElectricityIrregular Electricity:MO\_Residing | 3.15\* | 1.26 | 115.85\* |
| ElectricityNo Electricity:MO\_Residing | 8.05\* | 6.85 | 6.13 |
| ElectricityIrregular Electricity:Autoclave | -1.59 | -1.80 | -65.72 |
| ElectricityNo Electricity:Autoclave | -1.89 | -8.22 | -42.53 |
| ElectricityIrregular Electricity:RadiantWarmer | -5.65\*\*\* |  |  |
| ElectricityNo Electricity:RadiantWarmer | 24.64\*\*\* |  |  |
| ElectricityIrregular Electricity:DF\_Large |  | 5.11 | 63.06 |
| ElectricityNo Electricity:DF\_Large |  | 14.89 | 885.40\*\*\* |
| ElectricityIrregular Electricity:ILR\_Large |  | 11.57 | 49.01 |
| ElectricityNo Electricity:ILR\_Large |  | -6.65 | -66.44 |
| ElectricityIrregular Electricity:Centrifuge |  | -2.49 | 32.70 |
| ElectricityNo Electricity:Centrifuge |  | 11.23 | -19.56 |
| Constant | -26.80\*\*\* | -50.71\*\*\* | 366.23\*\* |
|  | | | |
| Observations | 7,805 | 4,540 | 4,782 |
| R2 | 0.50 | 0.57 | 0.57 |
| Adjusted R2 | 0.49 | 0.57 | 0.56 |
| Residual Std. Error | 29.72 (df = 7734) | 66.31 (df = 4466) | 817.57 (df = 4708) |
| F Statistic | 108.53\*\*\* (df = 70; 7734) | 81.86\*\*\* (df = 73; 4466) | 84.51\*\*\* (df = 73; 4708) |
|  | | | |
| *Note:* | \*p<0.1; \*\*p<0.05; \*\*\*p<0.01 | | |
