# Supplementary material for: Reduced health services at under-electrified primary healthcare facilities: Evidence from India
Source: PLoS One. 2021 Jun 4;16(6):e0252705. doi: 10.1371/journal.pone.0252705 (PMC8177862; doi:10.1371/journal.pone.0252705)
Supplement: S1 Replication materials — (ZIP) [file pone.0252705.s002.zip › Replication material - PLOS ONE Review - Revised/Results/All_Models_Linear_District_HDFE.html]

**All Models - Linear - High dimension fixed effect**

|  | | | |
|  | *Dependent variable:* | | |
|  |  | | |
|  | Deliveries | IPD | OPD |
|  | *felm* | *felm* | *felm* |
|  | (1) | (2) | (3) |
|  | | | |
| ElectricityIrregular Electricity | 2.14 | 10.47\* | 14.25 |
| ElectricityNo Electricity | -17.46\*\*\* | 13.98\* | -259.35\*\*\* |
| Generator | 1.86 | 5.89 | 137.34\*\*\* |
| Urban | -2.11 | -7.02\* | -48.43 |
| Population10000 | 1.69\*\*\* | 2.84\*\*\* | 35.53\*\*\* |
| `24x7` | 11.67\*\*\* | 19.64\*\*\* | 264.94\*\*\* |
| Beds | 0.10 | 2.06\*\*\* | 1.77 |
| MO\_Total | 8.06\*\*\* | 13.22\*\*\* | 286.68\*\*\* |
| LMO\_Total | -1.99\*\* | -0.61 | 71.34\* |
| Nurse\_Total | -1.77\*\*\* | -0.96 | 24.91 |
| LHV\_Total | 0.18 | 1.76 | -56.76\* |
| ANM\_Total | 0.76\*\*\* | -0.10 | 9.27 |
| Pharma\_Total | -1.81\*\* | 3.95 | -7.89 |
| MO\_Residing | 1.32 | 4.99 | -15.63 |
| Autoclave | 5.02\*\*\* | 3.26 | 90.68\*\* |
| RadiantWarmer | 11.81\*\*\* |  |  |
| DF\_Large |  | 9.74\*\* | -3.78 |
| ILR\_Large |  | -7.83 | 64.88 |
| Centrifuge |  | 10.24\*\*\* | 45.72 |
| Govt\_Building | 1.38 | 1.78 | 70.22 |
| Condition | -1.09 | -4.86\* | -48.12 |
| Water | 1.90\*\* | 1.43 | 26.26 |
| Toilet | -0.44 | 1.83 | 70.21\* |
| ElectricityIrregular Electricity:Generator | -0.40 | -3.67 | -32.21 |
| ElectricityNo Electricity:Generator | 15.26\*\*\* | 3.22 | 185.85 |
| ElectricityIrregular Electricity:`24x7` | -4.48\*\* | -11.13\*\* | -170.21\*\*\* |
| ElectricityNo Electricity:`24x7` | 3.11 | -14.22\* | -6.64 |
| ElectricityIrregular Electricity:MO\_Total | -3.86\*\*\* | -4.77\*\* | -108.92\*\*\* |
| ElectricityNo Electricity:MO\_Total | -8.76\*\*\* | -23.62\*\*\* | -206.21\*\*\* |
| ElectricityIrregular Electricity:LMO\_Total | 4.92\*\*\* | 3.27 | -87.43 |
| ElectricityNo Electricity:LMO\_Total | 6.63 | -3.30 | 42.34 |
| ElectricityIrregular Electricity:Nurse\_Total | 2.34\*\*\* | 3.53\* | -19.87 |
| ElectricityNo Electricity:Nurse\_Total | -0.38 | 7.04 | -227.36\*\*\* |
| ElectricityIrregular Electricity:LHV\_Total | 1.61\* | 0.88 | 113.09\*\*\* |
| ElectricityNo Electricity:LHV\_Total | 1.33 | -5.31 | 122.71 |
| ElectricityIrregular Electricity:ANM\_Total | -0.70\*\* | 0.62 | 13.04 |
| ElectricityNo Electricity:ANM\_Total | -1.34 | -2.89 | 72.05\*\* |
| ElectricityIrregular Electricity:Pharma\_Total | 3.73\*\*\* | -8.02\*\* | 44.49 |
| ElectricityNo Electricity:Pharma\_Total | 17.83\*\*\* | 7.57 | 229.27\*\*\* |
| ElectricityIrregular Electricity:MO\_Residing | 2.73 | 3.75 | 155.56\*\* |
| ElectricityNo Electricity:MO\_Residing | 8.08\* | 24.08\*\* | -23.69 |
| ElectricityIrregular Electricity:Autoclave | -0.87 | 0.61 | -35.07 |
| ElectricityNo Electricity:Autoclave | -1.36 | -10.17 | -78.25 |
| ElectricityIrregular Electricity:RadiantWarmer | -5.51\*\*\* |  |  |
| ElectricityNo Electricity:RadiantWarmer | 18.59\*\*\* |  |  |
| ElectricityIrregular Electricity:DF\_Large |  | 3.25 | 79.87 |
| ElectricityNo Electricity:DF\_Large |  | 17.50 | 606.47\*\* |
| ElectricityIrregular Electricity:ILR\_Large |  | 8.36 | 20.94 |
| ElectricityNo Electricity:ILR\_Large |  | -93.79\*\*\* | 180.09 |
| ElectricityIrregular Electricity:Centrifuge |  | -3.45 | 25.58 |
| ElectricityNo Electricity:Centrifuge |  | 19.84 | 219.83 |
|  | | | |
| Observations | 7,805 | 4,540 | 4,782 |
| R2 | 0.57 | 0.69 | 0.64 |
| Adjusted R2 | 0.53 | 0.66 | 0.61 |
| Residual Std. Error | 28.52 (df = 7227) | 58.61 (df = 4104) | 774.90 (df = 4338) |
|  | | | |
| *Note:* | \*p<0.1; \*\*p<0.05; \*\*\*p<0.01 | | |
