# Supplementary material for: Reduced health services at under-electrified primary healthcare facilities: Evidence from India
Source: PLoS One. 2021 Jun 4;16(6):e0252705. doi: 10.1371/journal.pone.0252705 (PMC8177862; doi:10.1371/journal.pone.0252705)
Supplement: S1 Replication materials — (ZIP) [file pone.0252705.s002.zip › Replication material - PLOS ONE Review - Revised/Results/All_Models_Linear_DistrictFE.html]

**All Models - Linear**

|  | | | |
|  | *Dependent variable:* | | |
|  |  | | |
|  | Deliveries | IPD | OPD |
|  | *OLS* | *OLS* | *OLS* |
|  | (1) | (2) | (3) |
|  | | | |
| ElectricityIrregular Electricity | 2.14 | 10.47\* | 14.25 |
| ElectricityNo Electricity | -17.46\*\*\* | 13.98\* | -259.35\*\*\* |
| Generator | 1.86 | 5.89 | 137.34\*\*\* |
| Urban | -2.11 | -7.02\* | -48.43 |
| Population10000 | 1.69\*\*\* | 2.84\*\*\* | 35.53\*\*\* |
| `24x7` | 11.67\*\*\* | 19.64\*\*\* | 264.94\*\*\* |
| Beds | 0.10 | 2.06\*\*\* | 1.77 |
| MO\_Total | 8.06\*\*\* | 13.22\*\*\* | 286.68\*\*\* |
| LMO\_Total | -1.99\*\* | -0.61 | 71.34\* |
| Nurse\_Total | -1.77\*\*\* | -0.96 | 24.91 |
| LHV\_Total | 0.18 | 1.76 | -56.76\* |
| ANM\_Total | 0.76\*\*\* | -0.10 | 9.27 |
| Pharma\_Total | -1.81\*\* | 3.95 | -7.89 |
| MO\_Residing | 1.32 | 4.99 | -15.63 |
| Autoclave | 5.02\*\*\* | 3.26 | 90.68\*\* |
| RadiantWarmer | 11.81\*\*\* |  |  |
| DF\_Large |  | 9.74\*\* | -3.78 |
| ILR\_Large |  | -7.83 | 64.88 |
| Centrifuge |  | 10.24\*\*\* | 45.72 |
| Govt\_Building | 1.38 | 1.78 | 70.22 |
| Condition | -1.09 | -4.86\* | -48.12 |
| Water | 1.90\*\* | 1.43 | 26.26 |
| Toilet | -0.44 | 1.83 | 70.21\* |
| DistrictAgra | 6.99 |  |  |
| DistrictAhmadnagar | -7.72 |  |  |
| DistrictAizawl | 2.20 | -11.11 | -748.57 |
| DistrictAjmer | 7.47 |  |  |
| DistrictAkola | -7.87 |  |  |
| DistrictAlappuzha | 9.12 | -2.84 | 2,500.39\*\*\* |
| DistrictAligarh | 14.32 | 6.67 | 186.17 |
| DistrictAllahabad | 7.95 | 79.86\*\*\* | 541.28 |
| DistrictAlmora | -8.73 | -19.20 | -440.38 |
| DistrictAlwar | 0.74 |  |  |
| DistrictAmbala | 1.40 | 54.77\*\* | 664.11\* |
| DistrictAmbedkar Nagar | 9.73 | 46.25\* | -20.26 |
| DistrictAmrawati | -15.84\* |  |  |
| DistrictAmritsar | 11.29 |  |  |
| DistrictAnantpur | 9.29 |  |  |
| DistrictAnjaw | 13.53 | 19.46 | -351.55 |
| DistrictAnugul | 6.80 | 5.15 | 227.03 |
| DistrictAraria | 41.69\*\*\* | 55.13 | 1,046.23\*\*\* |
| DistrictAriyalur | 11.27 | 259.74\*\*\* | 3,089.68\*\*\* |
| DistrictAuraiya | 11.33 | 7.61 | 327.35 |
| DistrictAurangabad | 10.87 | 328.34\*\*\* | 1,571.49\*\*\* |
| DistrictAzamgarh | 16.23\*\* | 10.05 | 149.87 |
| DistrictBagalkot | 4.72 | 12.03 | -121.37 |
| DistrictBageshwar | 5.64 | 10.26 | -285.56 |
| DistrictBaghpat | 13.78 | -1.20 | 169.56 |
| DistrictBahraich | 21.58\*\* | 14.49 | 334.53 |
| DistrictBalaghat | 16.93\* | 2.94 | -251.05 |
| DistrictBalangir | 14.60 | 13.57 | 327.90 |
| DistrictBaleshwar | 7.88 | 10.13 | 864.10\*\*\* |
| DistrictBallia | 16.54\* | 6.91 | -42.45 |
| DistrictBalrampur | 36.06\*\*\* | 51.85 | 644.99 |
| DistrictBanda | 21.60\*\* | 13.20 | 110.84 |
| DistrictBangalore | 9.19 | -8.08 | -71.26 |
| DistrictBangalore Rural | 4.48 | -3.31 | 49.37 |
| DistrictBanka | 117.09\*\*\* | 649.11\*\*\* | 1,345.30\*\*\* |
| DistrictBankura | 10.06 |  | 1,543.57\*\*\* |
| DistrictBanswara | 15.42\* |  |  |
| DistrictBarabanki | 16.20\*\* | 23.20 | 93.32 |
| DistrictBaran | 13.16 |  |  |
| DistrictBarddhaman | 12.63 |  | 393.17 |
| DistrictBareilly | 9.12 | 14.07 | -81.36 |
| DistrictBargarh | 13.33 | 11.28 | 329.41 |
| DistrictBarmer | 13.32\* |  |  |
| DistrictBarnala | 8.70 |  |  |
| DistrictBarpeta | 14.00 | -1.33 | 387.82 |
| DistrictBarwani | 33.53\*\*\* | 14.89 | 13.84 |
| DistrictBastar | 13.08 | 13.96 | -77.54 |
| DistrictBasti | 5.69 | 17.95 | -17.19 |
| DistrictBaudh | 5.23 | 3.32 | 698.25\* |
| DistrictBegusarai | 127.43\*\*\* | 122.56\*\*\* | 1,680.49\*\*\* |
| DistrictBelgaum | 3.96 | 15.34 | -292.99 |
| DistrictBellary | 8.49 | 7.36 | -176.14 |
| DistrictBetul | 12.60 | 1.99 | -72.56 |
| DistrictBhadrak | 10.72 | 15.48 | 1,016.70\*\*\* |
| DistrictBhagalpur | 90.63\*\*\* | 276.33\*\*\* | 1,957.25\*\*\* |
| DistrictBhandara | -9.75 |  |  |
| DistrictBharatpur | 9.51 |  |  |
| DistrictBhathinda | -15.21 |  |  |
| DistrictBhilwara | 7.96 |  |  |
| DistrictBhind | 29.16\*\*\* | 20.29 | -44.99 |
| DistrictBhiwani | 5.48 | -5.10 | -57.28 |
| DistrictBhojpur | 115.22\*\*\* | 305.71\*\*\* | 1,834.00\*\*\* |
| DistrictBhopal | -18.88 | -38.61 | -960.20\*\* |
| DistrictBid | -6.43 | 5.67 | 285.89 |
| DistrictBidar | 7.64 | -0.43 | -249.72 |
| DistrictBijapur | 8.80 | 33.45 | -118.61 |
| DistrictBijnor | 8.44 | 11.35 | 61.21 |
| DistrictBikaner | 10.95 |  |  |
| DistrictBilaspur | 9.24 | 4.12 | -278.69 |
| DistrictBirbhum | 8.50 | -34.24 | 856.89\* |
| DistrictBishnupur | -13.80 | 62.88\*\* | -752.94\* |
| DistrictBokaro | 21.16 | 0.19 | -195.02 |
| DistrictBongaigaon | 13.39\* | -4.97 | 227.98 |
| DistrictBudaun | 25.06\* | 11.05 | 71.19 |
| DistrictBulandshahar | 21.59\*\* | 26.44 | 229.47 |
| DistrictBuldana | -4.79 |  |  |
| DistrictBundi | 3.01 |  |  |
| DistrictBuxar | 69.04\*\*\* | 258.26\*\*\* | 1,333.92\*\*\* |
| DistrictCachar | -0.20 | -20.41 | 286.51 |
| DistrictChamarajanagar | 6.88 | 4.66 | -23.68 |
| DistrictChamba | 12.66 | 6.54 | 27.22 |
| DistrictChamoli | -10.31 | -6.00 | 98.98 |
| DistrictChampawat | -4.51 | -7.89 | -384.55 |
| DistrictChamphai | -1.18 | -14.50 | -744.43\* |
| DistrictChandauli | 7.39 | 5.08 | -188.01 |
| DistrictChandel | -9.82 | 1.71 | -771.65 |
| DistrictChandrapur | -10.53 |  |  |
| DistrictChanglang | -8.18 | -22.33 | -286.94 |
| DistrictChatra | 28.26\* | 13.56 | 227.36 |
| DistrictChhatarpur | 11.47 | 2.18 | -538.41 |
| DistrictChhindwara | 7.60 | -8.59 | -302.48 |
| DistrictChikkaballarpura | 13.91 | 14.49 | -50.85 |
| DistrictChikmagalur | 6.34 | -2.59 | -98.74 |
| DistrictChitoor | 12.79\* | -13.41 | 1,213.03 |
| DistrictChitradurga | 5.56 | -1.59 | 23.87 |
| DistrictChitrakoot | 16.67\* | 24.63 | 326.24 |
| DistrictChittaurgarh | 5.82 |  |  |
| DistrictChurachandpur | 8.06 | -3.84 | -348.24 |
| DistrictChuru | 7.33 |  |  |
| DistrictCoimbatore | -5.29 |  |  |
| DistrictCuddalore | -5.22 |  |  |
| DistrictCuttack | 13.11\* | 18.38 | 567.92\*\* |
| DistrictDakshin Dinajpur | 9.72 |  | 2,229.38\*\*\* |
| DistrictDakshina Kannada | 7.64 | -15.93 | -23.69 |
| DistrictDamoh | 35.09\*\*\* | 11.87 | -371.96 |
| DistrictDantewada | 9.77 | 10.79 | -5.64 |
| DistrictDarbhanga | 36.70\*\*\* | 246.95\*\*\* | 4,353.80\*\*\* |
| DistrictDarjiling | 2.92 |  | -184.18 |
| DistrictDarrang | 18.96 | -7.85 | 110.88 |
| DistrictDatai | 7.11 | 0.26 | -126.38 |
| DistrictDausa | 7.17 |  |  |
| DistrictDavangere | 8.95 | 8.79 | -206.32 |
| DistrictDebagarh | 16.63 | 10.00 | 332.13 |
| DistrictDehradun | -13.22 | -19.77 | -355.75 |
| DistrictDeoria | 7.77 | 12.57 | -322.37 |
| DistrictDewas | 32.92\*\*\* | 4.00 | -387.91 |
| DistrictDhalai | -1.68 | 29.67 | -434.32 |
| DistrictDhamtari | 7.28 | 5.43 | 43.56 |
| DistrictDhanbad | 13.69 | 1.46 | -88.18 |
| DistrictDhar | 4.75 | -7.75 | -394.86 |
| DistrictDharmapuri | 10.07 |  |  |
| DistrictDharwad | 10.74 | 52.32 | 715.75 |
| DistrictDhaulpur | 8.71 |  |  |
| DistrictDhemaji | 18.06\* | -29.94 | 353.10 |
| DistrictDhenkanal | 12.21 | 15.32 | 350.78 |
| DistrictDhubri | 8.51 | -15.19 | 1,232.21\*\*\* |
| DistrictDhule | -7.17 |  |  |
| DistrictDibrugarh | -1.94 | -23.26 | 294.87 |
| DistrictDimapur | -3.92 | -21.53 | -773.25 |
| DistrictDindigul | 9.00 |  |  |
| DistrictDindori | 10.96 | 8.97 | -235.56 |
| DistrictDumka | 9.23 | 4.11 | -186.84 |
| DistrictDungarpur | 11.53 |  |  |
| DistrictDurg | 8.71 | 2.56 | -230.75 |
| DistrictEast | -2.95 | -0.40 | -203.00 |
| DistrictEast Garo Hills | -3.10 | -16.19 | -363.12 |
| DistrictEast Godavari | 6.08 | 17.44 | 491.11 |
| DistrictEast Kameng | 16.54 | 4.62 | -222.99 |
| DistrictEast Khasi Hills | 70.76\*\*\* | 198.51\*\*\* | 123.45 |
| DistrictEast Nimar | 6.27 | -7.73 | -446.99 |
| DistrictEast Siang | 8.11 | 5.82 | -197.22 |
| DistrictErode | -16.54 |  |  |
| DistrictEtah | 26.31\*\*\* | 12.01 | 298.39 |
| DistrictEtawah | 18.74\*\* | 21.44 | 579.50\* |
| DistrictFaizabad | 10.24 | 8.96 | 630.60 |
| DistrictFaridabad | -9.02 | -18.12 | 319.57 |
| DistrictFaridkot | 4.23 |  |  |
| DistrictFarrukhabad | 12.62 | 12.56 | 619.14\* |
| DistrictFatehabad | 22.23\*\* | -2.14 | 149.29 |
| DistrictFatehgarh Sahib | -8.23 |  |  |
| DistrictFatehpur | 14.91 | 12.08 | 135.49 |
| DistrictFirozabad | 20.39\*\* | 10.73 | 90.86 |
| DistrictFirozpur | 9.23 |  |  |
| DistrictGadag | 7.92 | 18.53 | -152.54 |
| DistrictGadchiroli | -7.01 |  |  |
| DistrictGajapati | 7.08 | 9.00 | 444.18 |
| DistrictGanganagar | 6.03 |  |  |
| DistrictGanjam | 15.51\* | 14.37 | 347.26 |
| DistrictGarhwa | 28.60\*\* | 25.01 | 105.23 |
| DistrictGarhwal | -10.56 | -23.33 | -89.42 |
| DistrictGautam Buddha Nagar | 18.16\*\* | 12.64 | 231.42 |
| DistrictGaya | 131.92\*\*\* | 369.94\*\*\* | 1,901.99\*\*\* |
| DistrictGhaziabad | 4.23 | 3.71 | -187.41 |
| DistrictGhazipur | 15.16 | 1.33 | 228.52 |
| DistrictGiridih | 32.42 | 28.57 | -11.96 |
| DistrictGoalpara | 5.90 | -15.93 | 260.40 |
| DistrictGodda | 14.20 | 13.40 | -420.88 |
| DistrictGolaghat | 6.73 | -10.07 | 45.37 |
| DistrictGonda | 20.89\*\* | 14.75 | 432.61 |
| DistrictGondiya | -12.15 |  |  |
| DistrictGopalganj | 85.34\*\*\* | 386.10\*\*\* | 2,410.38\*\*\* |
| DistrictGorakhpur | 7.05 | 6.43 | -2.64 |
| DistrictGulbarga | 5.48 | 20.64 | -195.57 |
| DistrictGuna | 13.09 | -4.40 | -619.67\* |
| DistrictGuntur | 0.80 |  |  |
| DistrictGurgaon | 16.99 | 20.55 | 836.23\*\* |
| DistrictGwalior | 4.22 | 0.43 | -264.13 |
| DistrictHailakandi | -10.20 | -26.19 | 594.52 |
| DistrictHamirpur | 11.70 | 23.74 | 16.55 |
| DistrictHamumagarh | 0.50 |  |  |
| DistrictHaora | 14.22 | 15.13 | 1,951.03\*\*\* |
| DistrictHarda | 19.79 | 15.54 | -367.66 |
| DistrictHardoi | 51.38\*\*\* | 26.40 | -243.26 |
| DistrictHardwar | 9.54 | -4.03 | 922.78\* |
| DistrictHassan | 5.81 | -11.58 | -432.27 |
| DistrictHathras | 18.16\* | 9.73 | 284.81 |
| DistrictHaveri | 5.43 | 16.25 | -117.90 |
| DistrictHazaribagh | 15.12 | 1.91 | 45.92 |
| DistrictHingoli | -2.20 | -18.12 | -1,013.26 |
| DistrictHisar | 6.89 | 9.98 | 416.66 |
| DistrictHoshangabad | -0.40 | -4.32 | -519.64 |
| DistrictHoshiarpur | 9.36 | -10.46 | -620.72 |
| DistrictHugli | 6.69 |  |  |
| DistrictIdukki | 20.46\* | 311.22\*\*\* | -927.77\*\* |
| DistrictImphal East | -26.60\*\* | -57.98\* | -1,148.28\*\*\* |
| DistrictImphal West | -7.14 | -46.10 | -1,006.35\*\* |
| DistrictIndore | 13.46 | -14.34 | 424.49 |
| DistrictJabalpur | 6.94 | -4.38 | -458.66 |
| DistrictJagatsinghapur | 12.36 | 13.19 | 331.26 |
| DistrictJaintia Hills | -9.19 | -31.98 | -564.03\* |
| DistrictJaipur | 9.72 |  |  |
| DistrictJaisalmer | 7.12 |  |  |
| DistrictJajapur | 10.05 | 10.58 | 654.61\*\* |
| DistrictJalandhar | 8.81 |  |  |
| DistrictJalaun | 19.55 | 18.60 | 140.94 |
| DistrictJalgaon | -6.50 |  |  |
| DistrictJalna | -3.79 |  |  |
| DistrictJalore | 8.94 |  |  |
| DistrictJalpaiguri | 6.62 | 9.20 | 2,021.41\*\*\* |
| DistrictJamui | 105.10\*\*\* | 422.70\*\*\* | 2,331.50\*\*\* |
| DistrictJanjgir - Champa | 11.11 | 5.72 | -211.27 |
| DistrictJashpur | 10.82 | 29.58 | 54.77 |
| DistrictJaunpur | 17.31\* | 8.35 | -26.56 |
| DistrictJehanabad | 67.82\*\*\* | 267.14\*\*\* | 1,334.98\*\*\* |
| DistrictJhabua | 26.14\*\*\* | 10.82 | -329.32 |
| DistrictJhajjar | 9.36 | -3.09 | 206.85 |
| DistrictJhalawar | 5.86 |  |  |
| DistrictJhansi | 6.13 | -0.22 | -249.13 |
| DistrictJharsuguda | 2.52 | 14.82 | 70.41 |
| DistrictJhunjhunun | 6.42 |  |  |
| DistrictJind | 7.41 | 15.13 | 634.63\*\* |
| DistrictJodhpur | 14.46\* |  |  |
| DistrictJorhat | 1.71 | -7.38 | -117.86 |
| DistrictJyotiba Phule Nagar | 12.62 | 19.96 | 190.42 |
| DistrictKaimur (Bhabua) | 65.73\*\*\* | 275.94\*\*\* | 1,794.03\*\*\* |
| DistrictKaithal | 6.39 | 5.22 | 709.42\*\* |
| DistrictKalahandi | 9.81 | 22.59 | 385.12 |
| DistrictKamrup | 12.62\* | -5.36 | 313.68 |
| DistrictKancheepuram | 6.23 |  |  |
| DistrictKandhamal | 15.13 | 18.08 | 138.60 |
| DistrictKangra | 0.78 |  |  |
| DistrictKanker | 2.44 | 8.03 | -204.59 |
| DistrictKannauj | 18.39\*\* | 8.14 | 222.98 |
| DistrictKanniyakumari | -13.50 |  |  |
| DistrictKannur | 6.88 | 6.55 | 812.49\* |
| DistrictKanpur Dehat | 13.07 | 9.27 | 513.07 |
| DistrictKanpur Nagar | 13.31 | 9.64 | 134.77 |
| DistrictKapurthala | 17.12 |  |  |
| DistrictKaraikal | 1.77 |  |  |
| DistrictKarauli | -1.18 |  |  |
| DistrictKarbi Anglong | 22.57\*\* | -3.64 | -157.81 |
| DistrictKarimganj | 19.12 | -22.85 | 960.86\*\* |
| DistrictKarimnagar | -9.70 | -13.25 | 379.25 |
| DistrictKarnal | 9.18 | 10.75 | 496.47 |
| DistrictKarur | -7.90 |  |  |
| DistrictKasaragod | 42.20\*\*\* | 357.24\*\*\* | -364.25 |
| DistrictKatihar | 86.57\*\*\* | 169.88\*\*\* | 2,144.30\*\*\* |
| DistrictKatni | 19.17\* | 10.43 | -430.29 |
| DistrictKaushambi | 14.99 | 7.40 | -64.71 |
| DistrictKawardha | 9.14 | 8.80 | -99.43 |
| DistrictKendrapara | 10.29 | 11.23 | 522.86\* |
| DistrictKendujhar | 8.25 | 12.30 | 171.71 |
| DistrictKhagaria | 130.37\*\*\* | 242.28\*\*\* | 3,087.45\*\*\* |
| DistrictKhammam | -15.28 |  |  |
| DistrictKheri | 15.57\* | 12.83 | 347.81 |
| DistrictKhordha | 10.48 | 2.37 | 547.01\* |
| DistrictKinnaur | 12.51 |  |  |
| DistrictKiphire | 10.46 |  |  |
| DistrictKishanganj | 56.07\*\*\* | 85.48\*\* | 1,876.69\*\*\* |
| DistrictKoch Bihar | 2.84 | 4.61 | 2,155.26\*\*\* |
| DistrictKodagu | 1.14 | -9.58 | -210.16 |
| DistrictKodarma | 8.72 | 1.76 | -625.14 |
| DistrictKohima | -7.31 | -26.10 | -856.13\* |
| DistrictKokrajhar | 25.51\*\* | -2.06 | -230.31 |
| DistrictKolar | 3.15 | -1.85 | -63.26 |
| DistrictKOLASIB | 0.46 | 13.55 | -475.70 |
| DistrictKolhapur | 2.03 |  |  |
| Districtkollam | 8.88 | -16.30 | 2,601.13\*\*\* |
| DistrictKoppal | 6.53 | 13.56 | -239.20 |
| DistrictKoraput | 8.84 | 0.41 | -51.74 |
| DistrictKorba | 12.40 | 9.80 | -257.17 |
| DistrictKoriya | 16.66\* | 16.81 | 121.18 |
| DistrictKota | -0.50 |  |  |
| DistrictKozhikode | -3.03 |  |  |
| DistrictKrishna | -1.18 |  |  |
| DistrictKrishnagiri | -3.03 |  |  |
| DistrictKullu | 12.54 | 8.50 | 993.63\*\* |
| DistrictKurnool | 15.29\*\* | -6.63 | 467.33 |
| DistrictKurukshetra | -2.68 | 1.44 | 744.20\*\* |
| DistrictKurung Kamey | 14.46 | 17.08 | -337.09 |
| DistrictKushinagar | 13.43 | 4.63 | -39.77 |
| DistrictLahul | Spiti | 5.83 | -19.45 |
| DistrictLakhimpur | -1.17 | -23.78 | 242.17 |
| DistrictLakhisarai | 67.67\*\*\* | 350.86\*\*\* | 521.16 |
| DistrictLalitpur | 8.98 | 18.16 | 62.09 |
| DistrictLatur | -4.15 |  |  |
| DistrictLAWNGTLAI | 22.11 | -40.84 | 503.85 |
| DistrictLohardaga | 44.52\*\* | 37.80 | 136.62 |
| DistrictLohit | -4.44 | -13.40 | -360.85 |
| DistrictLongleng | 4.49 |  |  |
| DistrictLower Dibang Valley | -1.15 | -19.82 | -194.82 |
| DistrictLower Subansiri | 15.70 | -2.67 | -265.11 |
| DistrictLucknow | 12.07 | 2.50 | 249.11 |
| DistrictLUNGLEI | -4.46 | -14.10 | -717.79\* |
| DistrictMadhepura | 61.80\*\*\* | 77.69\*\* | 1,402.04\*\*\* |
| DistrictMadhubani | 28.12\*\*\* | 139.10\*\*\* | 786.06\*\* |
| DistrictMadurai | 4.02 |  |  |
| DistrictMahasamund | 7.66 | 3.46 | -116.62 |
| DistrictMahbubnagar | 1.50 | 36.80 | 967.38\*\* |
| DistrictMahendragarh | -1.16 | -9.57 | 55.73 |
| DistrictMahoba | 4.23 | 11.79 | -339.11 |
| DistrictMahrajganj | 5.41 | -1.48 | -314.97 |
| DistrictMainpuri | 18.93\*\* | 11.88 | 252.92 |
| DistrictMaldah | 1.41 |  | 1,875.22\*\*\* |
| DistrictMalkangiri | 16.45 | 20.27 | 338.85 |
| DistrictMallappuram | 29.15\*\*\* | 27.07 | 87.21 |
| DistrictMamit | -1.12 | -8.92 | -647.03\* |
| DistrictMandi | 15.84 |  |  |
| DistrictMandla | 7.28 | -5.75 | -444.20 |
| DistrictMandsaur | 10.09 | -2.30 | -409.76 |
| DistrictMandya | 6.25 | -9.27 | -211.41 |
| DistrictMarigaon | 11.45 | -28.30 | 547.45\* |
| DistrictMathura | 19.21\*\* | 10.65 | 205.39 |
| DistrictMau | 24.23\*\*\* | 19.25 | 85.50 |
| DistrictMayurbhanj | 10.16 | 13.43 | 630.65\*\* |
| DistrictMedak | -10.15 | 80.94\*\* | 1,313.04\*\*\* |
| DistrictMeerut | 7.90 | 2.88 | 192.17 |
| DistrictMewat | 37.61\*\*\* | 104.17\*\*\* | 576.96 |
| DistrictMirzapur | 8.60 | 12.31 | -130.72 |
| DistrictMoga | 9.72 | 7.44 | -243.58 |
| DistrictMokokchung | -1.82 |  |  |
| DistrictMon | -2.03 |  |  |
| DistrictMoradabad | 12.52 | 14.03 | 57.95 |
| DistrictMorena | 35.19\*\*\* | -6.86 | 11.42 |
| DistrictMuktsar | 9.58 |  |  |
| DistrictMunger | 73.52\*\*\* | 124.72\*\*\* | 2,269.28\*\*\* |
| DistrictMurshidabad | 11.70 |  |  |
| DistrictMuzaffarnagar | 10.69 | 14.95 | 221.93 |
| DistrictMuzaffarpur | 63.56\*\*\* | 254.19\*\*\* | 1,502.67\*\*\* |
| DistrictMysore | 6.51 | 15.13 | 68.48 |
| DistrictNabarangapur | 34.49\*\*\* | 20.65 | 232.01 |
| DistrictNadia | 8.76 |  | 2,824.51\*\*\* |
| DistrictNagaon | 21.21\*\* | -16.93 | 356.93 |
| DistrictNagapattinam | 5.07 |  |  |
| DistrictNagaur | 8.80 |  |  |
| DistrictNagpur | -8.81 |  |  |
| DistrictNainital | -23.31 | -34.81 | -934.68\*\* |
| DistrictNalanda | 24.71\*\*\* |  | 1,817.98\*\*\* |
| DistrictNalbari | 0.05 | -15.28 | 533.79 |
| DistrictNalgonda | -1.69 | 53.27\*\* | 1,582.58\*\*\* |
| DistrictNamakkal | -10.64 |  |  |
| DistrictNanded | 6.26 |  |  |
| DistrictNandurbar | -5.11 |  |  |
| DistrictNarsimhapur | 6.72 | -0.10 | 287.61 |
| DistrictNashik | -7.56 |  |  |
| DistrictNawada | 173.36\*\*\* | 314.91\*\*\* | 2,336.45\*\*\* |
| DistrictNayagarh | 13.39 | 10.20 | 644.75\*\* |
| DistrictNeemuch | 1.69 | -8.18 | -519.94 |
| DistrictNicobar | -7.93 |  |  |
| DistrictNilgiris | -3.37 |  |  |
| DistrictNizamabad | -6.03 | 49.38 | 953.52 |
| DistrictNorth | -7.57 | -27.08 | -496.41 |
| DistrictNorth | Middle Andaman | 0.32 |  |
| DistrictNorth 24 Parganas | -1.88 |  |  |
| DistrictNorth Cachar Hills | -4.29 | -29.63 | -554.50 |
| DistrictNorth Goa | -3.96 | -35.04 | -19.49 |
| DistrictNorth Tripura | -6.06 | 59.10\*\* | -518.69 |
| DistrictNuapada | 7.37 | 6.48 | 81.67 |
| DistrictOsmanabad | -5.28 |  |  |
| DistrictPALAKKAD | 20.20\*\* | 29.99 | -195.15 |
| DistrictPalamu | 30.80\*\* | 15.76 | -315.97 |
| DistrictPali | 2.36 |  |  |
| DistrictPalwal | 41.62\*\*\* | 35.81 | 1,067.78\*\*\* |
| DistrictPanchkula | 0.61 | 12.21 | 1,481.90\*\*\* |
| DistrictPanipath | -4.54 |  |  |
| DistrictPanna | 16.26 | -2.43 | -593.08 |
| DistrictPapumpare | 23.46\* | 25.33 | -253.84 |
| DistrictParbhani | -1.97 |  |  |
| DistrictParen | -0.06 |  |  |
| DistrictPaschim Medinipur | 1.51 |  | 425.80 |
| DistrictPashchim Champaran | 103.70\*\*\* | 181.83\*\*\* | 2,149.67\*\*\* |
| DistrictPashchimi Singhbhum | 36.39\*\* | 23.67 | 2.58 |
| DistrictPathanamthitta | -8.45 |  |  |
| DistrictPatiala | -14.93 |  |  |
| DistrictPatna | 46.12\*\*\* | 169.65\*\*\* | 1,627.04\*\*\* |
| DistrictPerambalur | -1.47 |  |  |
| DistrictPhek | 9.77 |  |  |
| DistrictPilibhit | 7.92 | 3.01 | -270.44 |
| DistrictPithoragarh | -10.79 | -8.04 | 12.82 |
| DistrictPondicherry | 26.48\*\* |  |  |
| DistrictPrakasam | -3.15 | -25.09 | 178.44 |
| DistrictPratapgarh | 10.43 | 9.37 | 145.83 |
| DistrictPudukkottai | 26.17\*\* |  |  |
| DistrictPune | 1.71 | 83.24 |  |
| DistrictPurba Champaran | 48.88\*\*\* | 37.68 | 1,470.12\*\*\* |
| DistrictPurba Mednipur | -0.34 | -32.84 | 1,046.21\*\*\* |
| DistrictPurbi Singhbhum | 14.35 | 8.07 | -146.20 |
| DistrictPuri | 13.09 | 14.15 | 436.63 |
| DistrictPurnia | 80.85\*\*\* | 412.00\*\*\* | 1,566.05\*\*\* |
| DistrictPuruliya | 1.88 |  | 380.96 |
| DistrictRae Bareli | 6.64 | 14.34 | -22.18 |
| DistrictRaichur | 5.02 | 10.44 | -262.86 |
| DistrictRaigarh | -0.45 | -5.55 | -335.05 |
| DistrictRaipur | 11.63 | 2.35 | -99.86 |
| DistrictRaisen | 10.89 | -9.43 | -461.04 |
| DistrictRajgarh | 25.29\*\*\* | 6.11 | -284.12 |
| DistrictRajnandgaon | 12.33 | 13.16 | -72.02 |
| DistrictRajsamand | 0.29 |  |  |
| DistrictRamanagara | 7.16 | 10.28 | -102.24 |
| DistrictRamanathapuram | -4.14 |  |  |
| DistrictRampur | 4.05 | -5.56 | 735.01\*\* |
| DistrictRanchi | 20.22 | 5.30 | 101.62 |
| DistrictRangareddy | -11.39 | -6.79 | 829.76\*\* |
| DistrictRatlam | 32.69\*\*\* | 35.10 | -258.11 |
| DistrictRatnagiri | -11.26 |  |  |
| DistrictRayagada | 12.66 | 22.20 | 548.94\* |
| DistrictRewa | 11.94 | -7.73 | -216.10 |
| DistrictRewari | -2.73 | -0.53 | 324.95 |
| DistrictRi Bhoi | -3.57 | -21.39 | -211.40 |
| DistrictRohtak | -3.71 | -11.38 | 386.86 |
| DistrictRohtas | 108.66\*\*\* | 303.83\*\*\* | 2,119.98\*\*\* |
| DistrictRupnagar | 3.39 |  |  |
| DistrictSagar | 15.85 | 2.68 | -520.85 |
| DistrictSaharanpur | 5.30 | 4.88 | 321.24 |
| DistrictSaharsa | 64.68\*\*\* | 284.02\*\*\* | 3,607.99\*\*\* |
| DistrictSahibganj | 29.67\* | 19.09 | -11.01 |
| DistrictSAIHA | 7.38 | -16.31 | -347.36 |
| DistrictSalem | -5.57 |  |  |
| DistrictSamastipur | 37.06\*\*\* | 494.93\*\*\* | 2,225.46\*\*\* |
| DistrictSambalpur | 12.04 | 13.27 | 787.66\*\* |
| DistrictSangli | -4.01 |  |  |
| DistrictSant Kabir Nagar | 12.44 | 19.75 | 430.76 |
| DistrictSant Ravidas Nagar Bhadohi | 7.52 | 26.64 | 146.86 |
| DistrictSaran | 108.45\*\*\* | 103.56\*\*\* | 689.54\* |
| DistrictSAS Nagar | 13.03 |  |  |
| DistrictSatara | -6.61 |  |  |
| DistrictSatna | 16.45\* | 3.74 | -113.96 |
| DistrictSawai Madhopur | 9.46 |  |  |
| DistrictSehore | 1.76 | -3.83 | -258.33 |
| DistrictSenapati | 1.29 | -19.21 | -405.11 |
| DistrictSeoni | 18.63\*\* | 2.55 | -283.70 |
| DistrictSERCHHIP | -0.34 | -19.76 | -801.09\* |
| DistrictShahdol | 7.05 | 16.44 | 27.79 |
| DistrictShahid Bhagat Singh Nagar | 11.91 |  |  |
| DistrictShahjahanpur | 8.78 | 2.52 | -195.65 |
| DistrictShajapur | 18.76 | -8.21 | -337.78 |
| DistrictSheikhpura | 32.83\*\*\* | 108.81\*\*\* | 767.78\*\* |
| DistrictSheohar | 27.59\*\* | 109.42\*\*\* | 2,155.25\*\*\* |
| DistrictSheopur | 23.03\* | 6.23 | -17.28 |
| DistrictShimla | 6.33 |  |  |
| DistrictShimoga | 9.40 | 14.25 | -80.93 |
| DistrictShivpuri | 63.53\*\*\* | 50.26\* | -313.21 |
| DistrictShrawasti | 28.55\*\* | 27.38 | 534.76 |
| DistrictSibsagar | 3.48 | -9.41 | -115.47 |
| DistrictSiddharthnagar | 19.79\*\* | 16.66 | 211.93 |
| DistrictSidhi | 23.11\*\* | 5.57 | -305.42 |
| DistrictSikar | -0.85 |  |  |
| DistrictSindhudurg | -4.28 |  |  |
| DistrictSirmaur | 13.07 |  |  |
| DistrictSirohi | 5.34 |  |  |
| DistrictSirsa | 1.55 |  |  |
| DistrictSitamarhi | 43.27\*\*\* | 339.47\*\*\* | 1,216.53\*\*\* |
| DistrictSitapur | 12.35 | 7.43 | -156.85 |
| DistrictSivaganga | -2.14 |  |  |
| DistrictSiwan | 131.37\*\*\* | 359.91\*\*\* | 3,569.10\*\*\* |
| DistrictSolan | 6.95 | -2.88 | -216.34 |
| DistrictSolapur | -6.72 |  |  |
| DistrictSonapur | 10.33 | 9.05 | 231.65 |
| DistrictSonbhadra | 15.45 | 15.92 | -260.59 |
| DistrictSonipath | 16.00\* | -26.61 | 131.56 |
| DistrictSonitpur | 6.29 | -14.11 | 219.32 |
| DistrictSouth | -12.10 | -11.38 | -365.97 |
| DistrictSouth 24 Parganas | 4.50 |  |  |
| DistrictSouth Andamana | -8.41 |  |  |
| DistrictSouth Garo Hills | -12.44 | -15.63 | -659.02\* |
| DistrictSouth Goa | -1.47 | -16.03 | -192.68 |
| DistrictSouth Tripura | -6.46 | 3.22 | -624.82 |
| DistrictSri Potti Sriramulu Nellore | 0.50 | 2.64 | 472.40 |
| DistrictSrikakulam | 16.66\* |  |  |
| DistrictSultanpur | 21.43\*\* | 9.91 | 114.21 |
| DistrictSundargarh | 7.06 | 6.38 | 122.35 |
| DistrictSupaul | 33.25\*\*\* | 381.90\*\*\* | 2,038.75\*\*\* |
| DistrictSurguja | 10.71 | 9.89 | -232.50 |
| DistrictTamenglong | -10.69 | -16.00 | -1,014.12\*\* |
| DistrictTaran Taran | 13.08 |  |  |
| DistrictTawang | 8.62 | 9.33 | -348.50 |
| DistrictTehri Garhwal | -12.33 | 7.17 | -325.82 |
| DistrictThane | -1.04 |  |  |
| DistrictThanjavur | -4.65 |  |  |
| DistrictTheni | -4.29 |  |  |
| DistrictThiruvallur | 3.51 |  |  |
| DistrictThiruvananthapuram | 13.73 | 27.27 | 2,624.27\*\*\* |
| DistrictThiruvarur | -6.37 |  |  |
| DistrictThoothukkudi | 0.19 | -13.55 |  |
| DistrictThoubal | -14.87 | -39.98 | -853.96\*\* |
| DistrictThrissur | 17.84\* | 8.39 | 1,063.59\*\* |
| DistrictTikamgarh | 27.15\*\* | 17.53 | -387.89 |
| DistrictTinsukia | 3.69 | -27.42 | 151.88 |
| DistrictTirap | 5.90 | -17.20 | -9.27 |
| DistrictTiruchirappalli | 7.46 |  |  |
| DistrictTirunelveli | -7.93 |  |  |
| DistrictTiruppur | -5.50 |  |  |
| DistrictTiruvannamalai | 0.82 |  |  |
| DistrictTonk | 8.57 |  |  |
| DistrictTuensang | 11.08 |  |  |
| DistrictTumkur | 2.52 | -2.25 | -149.08 |
| DistrictUdaipur | 15.32\* |  |  |
| DistrictUdham Singh Nagar | 1.95 | -7.28 | -335.00 |
| DistrictUdupi | 2.12 | 1.39 | 289.08 |
| DistrictUjjain | -2.84 | -1.30 | -98.98 |
| DistrictUkhrul | -2.68 | 5.82 | -676.82\* |
| DistrictUmaria | 22.89\*\* | 11.09 | -223.72 |
| DistrictUna | 5.47 | -48.20 | 14.74 |
| DistrictUnnao | 13.03 | 11.62 | 57.02 |
| DistrictUpper Siang | 2.47 | 2.75 | -418.14 |
| DistrictUpper Subansiri | 26.93\*\* | 20.01 | -45.37 |
| DistrictUttar Dinajpur | 19.71 |  | 2,656.37\*\*\* |
| DistrictUttara Kannada | 11.59 | 5.98 | -177.07 |
| DistrictUttarkashi | -21.14 | -0.51 | -226.18 |
| DistrictVaishali | 71.82\*\*\* | 217.13\*\*\* | 2,278.20\*\*\* |
| DistrictVaranasi | 4.70 | 6.81 | -122.04 |
| DistrictVellore | 9.18 |  |  |
| DistrictVidisha | 17.70 | 178.60\*\*\* | -369.24 |
| DistrictViluppuram | 10.04 |  |  |
| DistrictVirudhunagar | 3.12 |  |  |
| DistrictVishakapatnam | 2.89 | 9.91 | 375.98 |
| DistrictVizianagaram | 3.63 |  |  |
| DistrictWardha | -14.73\* |  |  |
| DistrictWarngal | 1.31 |  |  |
| DistrictWashim | -11.45 |  |  |
| DistrictWayanand | 11.05 | 3.40 | 304.27 |
| DistrictWest | -3.85 | 8.00 | -218.77 |
| DistrictWest Garo Hills | -11.92 | -26.18 | -439.25 |
| DistrictWest Godavari | 0.62 | -1.66 | 55.51 |
| DistrictWest Kameng | 1.36 | -10.34 | -333.94 |
| DistrictWest Khasi Hills | -14.90 | -41.67 | -488.06 |
| DistrictWest Nimar | 21.72\*\*\* | -2.72 | -260.38 |
| DistrictWest Siang | 6.46 | -2.72 | -247.85 |
| DistrictWest Tripura | -15.62 | 32.89 | -622.87 |
| DistrictWokha | 12.00 |  |  |
| DistrictY.S.R. | 2.49 | -45.42 | -947.66 |
| DistrictYadgir | 17.81\*\* | 10.48 | -73.86 |
| DistrictYamunanagar | -2.18 | -14.09 | 104.99 |
| DistrictYavatmal | 1.14 |  |  |
| DistrictZunheboto | 7.08 |  |  |
| ElectricityIrregular Electricity:Generator | -0.40 | -3.67 | -32.21 |
| ElectricityNo Electricity:Generator | 15.26\*\*\* | 3.22 | 185.85 |
| ElectricityIrregular Electricity:`24x7` | -4.48\*\* | -11.13\*\* | -170.21\*\*\* |
| ElectricityNo Electricity:`24x7` | 3.11 | -14.22\* | -6.64 |
| ElectricityIrregular Electricity:MO\_Total | -3.86\*\*\* | -4.77\*\* | -108.92\*\*\* |
| ElectricityNo Electricity:MO\_Total | -8.76\*\*\* | -23.62\*\*\* | -206.21\*\*\* |
| ElectricityIrregular Electricity:LMO\_Total | 4.92\*\*\* | 3.27 | -87.43 |
| ElectricityNo Electricity:LMO\_Total | 6.63 | -3.30 | 42.34 |
| ElectricityIrregular Electricity:Nurse\_Total | 2.34\*\*\* | 3.53\* | -19.87 |
| ElectricityNo Electricity:Nurse\_Total | -0.38 | 7.04 | -227.36\*\*\* |
| ElectricityIrregular Electricity:LHV\_Total | 1.61\* | 0.88 | 113.09\*\*\* |
| ElectricityNo Electricity:LHV\_Total | 1.33 | -5.31 | 122.71 |
| ElectricityIrregular Electricity:ANM\_Total | -0.70\*\* | 0.62 | 13.04 |
| ElectricityNo Electricity:ANM\_Total | -1.34 | -2.89 | 72.05\*\* |
| ElectricityIrregular Electricity:Pharma\_Total | 3.73\*\*\* | -8.02\*\* | 44.49 |
| ElectricityNo Electricity:Pharma\_Total | 17.83\*\*\* | 7.57 | 229.27\*\*\* |
| ElectricityIrregular Electricity:MO\_Residing | 2.73 | 3.75 | 155.56\*\* |
| ElectricityNo Electricity:MO\_Residing | 8.08\* | 24.08\*\* | -23.69 |
| ElectricityIrregular Electricity:Autoclave | -0.87 | 0.61 | -35.07 |
| ElectricityNo Electricity:Autoclave | -1.36 | -10.17 | -78.25 |
| ElectricityIrregular Electricity:RadiantWarmer | -5.51\*\*\* |  |  |
| ElectricityNo Electricity:RadiantWarmer | 18.59\*\*\* |  |  |
| ElectricityIrregular Electricity:DF\_Large |  | 3.25 | 79.87 |
| ElectricityNo Electricity:DF\_Large |  | 17.50 | 606.47\*\* |
| ElectricityIrregular Electricity:ILR\_Large |  | 8.36 | 20.94 |
| ElectricityNo Electricity:ILR\_Large |  | -93.79\*\*\* | 180.09 |
| ElectricityIrregular Electricity:Centrifuge |  | -3.45 | 25.58 |
| ElectricityNo Electricity:Centrifuge |  | 19.84 | 219.83 |
| Constant | -24.02\*\*\* | -32.54 | -4.02 |
|  | | | |
| Observations | 7,805 | 4,540 | 4,782 |
| R2 | 0.57 | 0.69 | 0.64 |
| Adjusted R2 | 0.53 | 0.66 | 0.61 |
| Residual Std. Error | 28.52 (df = 7227) | 58.61 (df = 4104) | 774.90 (df = 4338) |
| F Statistic | 16.32\*\*\* (df = 577; 7227) | 21.28\*\*\* (df = 435; 4104) | 17.54\*\*\* (df = 443; 4338) |
|  | | | |
| *Note:* | \*p<0.1; \*\*p<0.05; \*\*\*p<0.01 | | |
