# Supplementary material for: Reduced health services at under-electrified primary healthcare facilities: Evidence from India
Source: PLoS One. 2021 Jun 4;16(6):e0252705. doi: 10.1371/journal.pone.0252705 (PMC8177862; doi:10.1371/journal.pone.0252705)
Supplement: S1 Replication materials — (ZIP) [file pone.0252705.s002.zip › Replication material - PLOS ONE Review - Revised/Results/All_Models_Linear_Parsimonious.html]

**All Models - Linear - Parsimonious**

|  | | | |
|  | *Dependent variable:* | | |
|  |  | | |
|  | Deliveries | IPD | OPD |
|  | *OLS* | *OLS* | *OLS* |
|  | (1) | (2) | (3) |
|  | | | |
| ElectricityIrregular Electricity | -5.22\*\*\* | -4.65\* | -239.68\*\*\* |
| ElectricityNo Electricity | -30.22\*\*\* | -26.17\*\*\* | -815.75\*\*\* |
| StateAndra Pradesh | 11.26 |  |  |
| StateArunachal Pradesh | 6.40 | -13.63 | -902.84\*\*\* |
| StateAssam | 11.02 | -20.93\*\* | -241.25\* |
| StateBihar | 107.69\*\*\* | 317.07\*\*\* | 2,156.28\*\*\* |
| StateChhattisgarh | 3.10 | -7.46 | -1,001.01\*\*\* |
| StateGoa | 7.16 | 6.85 | -16.57 |
| StateHaryana | 16.55\* | 11.73 | 12.59 |
| StateHimachal Pradesh | -3.17 | -24.80 | -839.75\*\*\* |
| StateJharkhand | 21.31\*\* | -3.45 | -692.59\*\*\* |
| StateKarnataka | 8.73 | 2.18 | -694.66\*\*\* |
| StateKerala | 8.04 | 85.27\*\*\* | -165.81 |
| StateMadhya Pradesh | 18.87\*\* | -0.07 | -908.61\*\*\* |
| StateMaharashtra | 5.21 | 37.12 | -979.87 |
| StateManipur | 6.24 | 0.24 | -890.83\*\*\* |
| StateMeghalaya | 5.95 | 16.37 | -676.74\*\*\* |
| StateMizoram | 1.86 | 2.81 | -1,071.11\*\*\* |
| StateNagaland | 0.83 | -16.95 | -1,129.96\*\*\* |
| StateOdisha | 3.24 | -18.90\* | -370.93\*\*\* |
| StatePuducherry | 13.06 |  |  |
| StatePunjab | 8.08 | -27.39 | -1,249.82\*\*\* |
| StateRajasthan | 8.17 |  |  |
| StateSikkim | -0.64 | 4.61 | -868.69\*\*\* |
| StateTamil Nadu | 8.04 | 152.78\*\*\* | 2,415.76\*\*\* |
| StateTelangana | 6.73 | 50.32\*\*\* | 810.96\*\*\* |
| StateTripura | 4.19 | 53.82\*\*\* | -795.52\*\*\* |
| StateUttar Pradesh | 13.25 | -3.33 | -493.36\*\*\* |
| StateUttrakhand | 3.25 | -4.39 | -686.31\*\*\* |
| StateWest Bengal | -0.30 | 6.64 | 1,028.05\*\*\* |
| Constant | 6.02 | 28.55\*\*\* | 1,510.24\*\*\* |
|  | | | |
| Observations | 7,805 | 4,540 | 4,782 |
| R2 | 0.34 | 0.50 | 0.45 |
| Adjusted R2 | 0.34 | 0.49 | 0.45 |
| Residual Std. Error | 33.81 (df = 7774) | 71.61 (df = 4512) | 917.04 (df = 4754) |
| F Statistic | 135.73\*\*\* (df = 30; 7774) | 164.49\*\*\* (df = 27; 4512) | 144.14\*\*\* (df = 27; 4754) |
|  | | | |
| *Note:* | \*p<0.1; \*\*p<0.05; \*\*\*p<0.01 | | |
