# Supplementary material for: Reduced health services at under-electrified primary healthcare facilities: Evidence from India
Source: PLoS One. 2021 Jun 4;16(6):e0252705. doi: 10.1371/journal.pone.0252705 (PMC8177862; doi:10.1371/journal.pone.0252705)
Supplement: S1 Replication materials — (ZIP) [file pone.0252705.s002.zip › Replication material - PLOS ONE Review - Revised/Results/All_Models_NoInteractions.html]

**All Models - No interactions**

|  | | | |
|  | *Dependent variable:* | | |
|  |  | | |
|  | Deliveries | IPD | OPD |
|  | *zero-inflated* | *zero-inflated* | *negative* |
|  | *count data* | *count data* | *binomial* |
|  | (1) | (2) | (3) |
|  | | | |
| ElectricityIrregular Electricity | 1.14\*\*\* | 1.05 | 0.93\*\*\* |
| ElectricityNo Electricity | 0.71\*\*\* | 0.90 | 0.80\*\*\* |
| Generator | 1.01 | 1.23\*\*\* | 1.18\*\*\* |
| Urban | 0.79\*\*\* | 0.78\*\*\* | 0.96 |
| Population10000 | 1.05\*\*\* | 1.02\*\*\* | 1.02\*\*\* |
| `24x7` | 1.49\*\*\* | 1.28\*\*\* | 1.06\*\* |
| Beds | 1.01\*\* | 1.05\*\*\* | 1.00 |
| MO\_Total | 1.03 | 1.09\*\*\* | 1.12\*\*\* |
| LMO\_Total | 0.97 | 0.95 | 0.99 |
| Nurse\_Total | 1.04\*\*\* | 1.08\*\*\* | 1.05\*\*\* |
| LHV\_Total | 1.08\*\*\* | 1.07\*\* | 1.03 |
| ANM\_Total | 1.04\*\*\* | 1.00 | 1.03\*\*\* |
| Pharma\_Total | 1.01 | 1.02 | 1.10\*\*\* |
| MO\_Residing | 1.21\*\*\* | 1.35\*\*\* | 1.12\*\*\* |
| Autoclave | 1.08\*\* | 1.05 | 1.07\*\*\* |
| RadiantWarmer | 1.33\*\*\* |  |  |
| DF\_Large |  | 1.07 | 1.08\* |
| ILR\_Large |  | 1.11 | 1.03 |
| Centrifuge |  | 1.22\*\*\* | 1.16\*\*\* |
| Govt\_Building | 0.97 | 1.13 | 1.02 |
| Condition | 0.94 | 0.95 | 1.00 |
| Water | 1.10\*\* | 0.94 | 1.06\*\* |
| Toilet | 0.78\*\*\* | 0.84\*\* | 1.14\*\*\* |
| StateAndra Pradesh | 5.07\*\*\* |  |  |
| StateArunachal Pradesh | 0.81 | 0.25\*\*\* | 0.27\*\*\* |
| StateAssam | 6.34\*\*\* | 0.31\*\*\* | 0.92 |
| StateBihar | 26.76\*\*\* | 5.99\*\*\* | 1.85\*\*\* |
| StateChhattisgarh | 3.62\*\*\* | 0.70\* | 0.45\*\*\* |
| StateGoa | 5.30\*\*\* | 0.57 | 0.66\* |
| StateHaryana | 6.31\*\*\* | 1.00 | 1.02 |
| StateHimachal Pradesh | 2.15\*\* | 0.21\*\*\* | 0.64\*\* |
| StateJharkhand | 8.49\*\*\* | 0.77 | 0.57\*\*\* |
| StateKarnataka | 3.97\*\*\* | 0.98 | 0.50\*\*\* |
| StateKerala | 7.79\*\*\* | 2.41\*\*\* | 0.85 |
| StateMadhya Pradesh | 8.92\*\*\* | 0.90 | 0.42\*\*\* |
| StateMaharashtra | 3.56\*\*\* | 1.34 | 0.08\*\*\* |
| StateManipur | 1.89\* | 1.14 | 0.19\*\*\* |
| StateMeghalaya | 2.76\*\*\* | 0.70 | 0.47\*\*\* |
| StateMizoram | 1.56 | 0.46\*\*\* | 0.22\*\*\* |
| StateNagaland | 1.00 | 0.21\*\* | 0.18\*\*\* |
| StateOdisha | 5.33\*\*\* | 1.09 | 1.16 |
| StatePuducherry | 14.02\*\*\* |  |  |
| StatePunjab | 5.04\*\*\* | 0.0000 | 0.23\*\*\* |
| StateRajasthan | 4.05\*\*\* |  |  |
| StateSikkim | 1.36 | 0.55\*\* | 0.42\*\*\* |
| StateTamil Nadu | 4.49\*\*\* | 10.31\*\*\* | 3.96\*\*\* |
| StateTelangana | 3.24\*\*\* | 1.52\* | 1.29\* |
| StateTripura | 2.66\*\*\* | 1.27 | 0.47\*\*\* |
| StateUttar Pradesh | 7.50\*\*\* | 1.07 | 0.79\*\* |
| StateUttrakhand | 2.67\*\*\* | 0.60\*\* | 0.50\*\*\* |
| StateWest Bengal | 2.99\*\*\* | 0.60 | 2.59\*\*\* |
| Constant | 1.46 | 14.60\*\*\* | 503.70\*\*\* |
|  | | | |
| Observations | 7,805 | 4,540 | 4,782 |
| Log Likelihood | -22,460.46 | -14,418.54 | -35,853.82 |
| theta |  |  | 1.86\*\*\* (0.04) |
| Akaike Inf. Crit. |  |  | 71,803.64 |
|  | | | |
| *Note:* | \*p<0.1; \*\*p<0.05; \*\*\*p<0.01 | | |
