# Supplementary material for: Reduced health services at under-electrified primary healthcare facilities: Evidence from India
Source: PLoS One. 2021 Jun 4;16(6):e0252705. doi: 10.1371/journal.pone.0252705 (PMC8177862; doi:10.1371/journal.pone.0252705)
Supplement: S1 Replication materials — (ZIP) [file pone.0252705.s002.zip › Replication material - PLOS ONE Review - Revised/Results/All_Models_NoInteractions_5cat.html]

**All Models - No interactions - Electricity 5 categories**

|  | | | |
|  | *Dependent variable:* | | |
|  |  | | |
|  | Deliveries | IPD | OPD |
|  | *zero-inflated* | *zero-inflated* | *negative* |
|  | *count data* | *count data* | *binomial* |
|  | (1) | (2) | (3) |
|  | | | |
| Electricity\_5catOccasional Powercut | 1.14\*\*\* | 1.00 | 0.96 |
| Electricity\_5catSummer Powercut | 1.01 | 0.93 | 0.94 |
| Electricity\_5catRegular Powercut | 1.16\*\*\* | 1.13\* | 0.89\*\*\* |
| Electricity\_5catNo Electricity | 0.71\*\*\* | 0.90 | 0.81\*\*\* |
| Generator | 1.01 | 1.23\*\*\* | 1.18\*\*\* |
| Urban | 0.79\*\*\* | 0.78\*\*\* | 0.96 |
| Population10000 | 1.05\*\*\* | 1.02\*\*\* | 1.02\*\*\* |
| `24x7` | 1.49\*\*\* | 1.28\*\*\* | 1.07\*\* |
| Beds | 1.01\*\* | 1.05\*\*\* | 1.00 |
| MO\_Total | 1.03 | 1.09\*\*\* | 1.12\*\*\* |
| LMO\_Total | 0.97 | 0.95 | 0.99 |
| Nurse\_Total | 1.04\*\*\* | 1.08\*\*\* | 1.05\*\*\* |
| LHV\_Total | 1.08\*\*\* | 1.07\* | 1.03 |
| ANM\_Total | 1.04\*\*\* | 1.00 | 1.03\*\*\* |
| Pharma\_Total | 1.01 | 1.02 | 1.10\*\*\* |
| MO\_Residing | 1.22\*\*\* | 1.35\*\*\* | 1.12\*\*\* |
| Autoclave | 1.09\*\* | 1.05 | 1.07\*\*\* |
| RadiantWarmer | 1.33\*\*\* |  |  |
| DF\_Large |  | 1.07 | 1.08\* |
| ILR\_Large |  | 1.11 | 1.03 |
| Centrifuge |  | 1.22\*\*\* | 1.16\*\*\* |
| Govt\_Building | 0.97 | 1.14 | 1.02 |
| Condition | 0.94 | 0.95 | 1.00 |
| Water | 1.10\*\* | 0.94 | 1.06\*\* |
| Toilet | 0.78\*\*\* | 0.84\*\* | 1.14\*\*\* |
| StateAndra Pradesh | 5.07\*\*\* |  |  |
| StateArunachal Pradesh | 0.81 | 0.25\*\*\* | 0.27\*\*\* |
| StateAssam | 6.35\*\*\* | 0.31\*\*\* | 0.91 |
| StateBihar | 26.85\*\*\* | 6.09\*\*\* | 1.82\*\*\* |
| StateChhattisgarh | 3.63\*\*\* | 0.73 | 0.44\*\*\* |
| StateGoa | 5.30\*\*\* | 0.59 | 0.65\*\* |
| StateHaryana | 6.30\*\*\* | 1.00 | 1.02 |
| StateHimachal Pradesh | 2.15\*\* | 0.21\*\*\* | 0.63\*\* |
| StateJharkhand | 8.51\*\*\* | 0.79 | 0.56\*\*\* |
| StateKarnataka | 3.94\*\*\* | 0.96 | 0.50\*\*\* |
| StateKerala | 7.81\*\*\* | 2.48\*\*\* | 0.84 |
| StateMadhya Pradesh | 8.95\*\*\* | 0.93 | 0.41\*\*\* |
| StateMaharashtra | 3.56\*\*\* | 1.37 | 0.08\*\*\* |
| StateManipur | 1.89\* | 1.20 | 0.19\*\*\* |
| StateMeghalaya | 2.75\*\*\* | 0.70 | 0.46\*\*\* |
| StateMizoram | 1.56 | 0.48\*\*\* | 0.21\*\*\* |
| StateNagaland | 1.00 | 0.22\*\* | 0.17\*\*\* |
| StateOdisha | 5.32\*\*\* | 1.09 | 1.16 |
| StatePuducherry | 14.05\*\*\* |  |  |
| StatePunjab | 5.14\*\*\* | 0.0000 | 0.23\*\*\* |
| StateRajasthan | 4.09\*\*\* |  |  |
| StateSikkim | 1.39 | 0.58\* | 0.41\*\*\* |
| StateTamil Nadu | 4.52\*\*\* | 10.60\*\*\* | 3.90\*\* |
| StateTelangana | 3.23\*\*\* | 1.48\* | 1.32\* |
| StateTripura | 2.68\*\*\* | 1.30 | 0.46\*\*\* |
| StateUttar Pradesh | 7.49\*\*\* | 1.11 | 0.78\*\* |
| StateUttrakhand | 2.68\*\*\* | 0.63\*\* | 0.49\*\*\* |
| StateWest Bengal | 2.99\*\*\* | 0.62 | 2.58\*\*\* |
| Constant | 1.44 | 14.17\*\*\* | 513.04\*\*\* |
|  | | | |
| Observations | 7,805 | 4,540 | 4,782 |
| Log Likelihood | -22,459.68 | -14,416.63 | -35,851.48 |
| theta |  |  | 1.86\*\*\* (0.04) |
| Akaike Inf. Crit. |  |  | 71,802.95 |
|  | | | |
| *Note:* | \*p<0.1; \*\*p<0.05; \*\*\*p<0.01 | | |
