# Supplementary material for: Reduced health services at under-electrified primary healthcare facilities: Evidence from India
Source: PLoS One. 2021 Jun 4;16(6):e0252705. doi: 10.1371/journal.pone.0252705 (PMC8177862; doi:10.1371/journal.pone.0252705)
Supplement: S1 Replication materials — (ZIP) [file pone.0252705.s002.zip › Replication material - PLOS ONE Review - Revised/Results/All_Models_Parsimonious.html]

**All Models - Parsimonious**

|  | | | |
|  | *Dependent variable:* | | |
|  |  | | |
|  | Deliveries | IPD | OPD |
|  | *zero-inflated* | *zero-inflated* | *negative* |
|  | *count data* | *count data* | *binomial* |
|  | (1) | (2) | (3) |
|  | | | |
| ElectricityIrregular Electricity | 1.08\*\* | 0.99 | 0.87\*\*\* |
| ElectricityNo Electricity | 0.52\*\*\* | 0.62\*\*\* | 0.61\*\*\* |
| Generator | 1.25\*\*\* | 1.50\*\*\* | 1.42\*\*\* |
| StateAndra Pradesh | 4.46\*\*\* |  |  |
| StateArunachal Pradesh | 0.66 | 0.32\*\*\* | 0.22\*\*\* |
| StateAssam | 4.73\*\*\* | 0.37\*\*\* | 0.79\*\* |
| StateBihar | 46.15\*\*\* | 10.63\*\*\* | 2.42\*\*\* |
| StateChhattisgarh | 2.20\*\* | 0.64\*\* | 0.32\*\*\* |
| StateGoa | 5.23\*\*\* | 1.61 | 1.02 |
| StateHaryana | 5.50\*\*\* | 1.15 | 0.95 |
| StateHimachal Pradesh | 1.14 | 0.18\*\*\* | 0.54\*\*\* |
| StateJharkhand | 5.86\*\*\* | 0.68 | 0.41\*\*\* |
| StateKarnataka | 3.38\*\*\* | 1.02 | 0.45\*\*\* |
| StateKerala | 7.04\*\*\* | 5.76\*\*\* | 0.83 |
| StateMadhya Pradesh | 7.02\*\*\* | 0.96 | 0.31\*\*\* |
| StateMaharashtra | 2.88\*\*\* | 1.69 | 0.07\*\*\* |
| StateManipur | 1.14 | 1.34 | 0.22\*\*\* |
| StateMeghalaya | 3.33\*\*\* | 1.66\*\* | 0.67\*\*\* |
| StateMizoram | 1.23 | 0.80 | 0.21\*\*\* |
| StateNagaland | 0.71 | 0.41 | 0.19\*\*\* |
| StateOdisha | 3.62\*\*\* | 0.88 | 0.75\*\*\* |
| StatePuducherry | 7.25\*\*\* |  |  |
| StatePunjab | 3.93\*\*\* | 0.0000 | 0.13\*\*\* |
| StateRajasthan | 3.00\*\*\* |  |  |
| StateSikkim | 1.26 | 0.94 | 0.40\*\*\* |
| StateTamil Nadu | 3.51\*\*\* | 7.41\*\* | 2.96\* |
| StateTelangana | 3.23\*\*\* | 2.07\*\*\* | 1.49\*\*\* |
| StateTripura | 3.17\*\*\* | 3.17\*\*\* | 0.51\*\*\* |
| StateUttar Pradesh | 5.29\*\*\* | 0.95 | 0.64\*\*\* |
| StateUttrakhand | 2.39\*\*\* | 0.79 | 0.48\*\*\* |
| StateWest Bengal | 1.96\*\* | 0.91 | 2.05\*\*\* |
| Constant | 3.10\*\*\* | 25.84\*\*\* | 1,159.33\*\*\* |
|  | | | |
| Observations | 7,805 | 4,540 | 4,782 |
| Log Likelihood | -22,798.99 | -14,594.79 | -36,114.04 |
| theta |  |  | 1.68\*\*\* (0.03) |
| Akaike Inf. Crit. |  |  | 72,286.09 |
|  | | | |
| *Note:* | \*p<0.1; \*\*p<0.05; \*\*\*p<0.01 | | |
