# Supplementary material for: Reduced health services at under-electrified primary healthcare facilities: Evidence from India
Source: PLoS One. 2021 Jun 4;16(6):e0252705. doi: 10.1371/journal.pone.0252705 (PMC8177862; doi:10.1371/journal.pone.0252705)
Supplement: S1 Replication materials — (ZIP) [file pone.0252705.s002.zip › Replication material - PLOS ONE Review - Revised/Results/All_Models_Subsample_Irr_No.html]

**All Models - Subsample - Irregular and No Electricity**

|  | | | |
|  | *Dependent variable:* | | |
|  |  | | |
|  | Deliveries | IPD | OPD |
|  | *zero-inflated* | *zero-inflated* | *negative* |
|  | *count data* | *count data* | *binomial* |
|  | (1) | (2) | (3) |
|  | | | |
| ElectricityNo Electricity | 0.45\*\*\* | 1.10 | 0.70\*\*\* |
| Generator | 0.96 | 1.24 | 1.10\*\*\* |
| Urban | 0.74\*\*\* | 0.94 | 0.97 |
| Population10000 | 1.06\*\*\* | 1.02 | 1.03\*\*\* |
| `24x7` | 1.47\*\*\* | 1.34 | 1.07\* |
| Beds | 1.01 | 1.02 | 1.01\*\* |
| MO\_Total | 1.07\*\* | 1.09 | 1.12\*\*\* |
| LMO\_Total | 0.94 | 0.94 | 1.00 |
| Nurse\_Total | 1.06\*\*\* | 1.07 | 1.02 |
| LHV\_Total | 1.04 | 1.14 | 1.05\* |
| ANM\_Total | 1.02 | 0.98 | 1.02\*\* |
| Pharma\_Total | 1.00 | 1.05 | 1.13\*\*\* |
| MO\_Residing | 1.32\*\*\* | 1.42 | 1.19\*\*\* |
| Autoclave | 1.08 | 1.00 | 1.04 |
| RadiantWarmer | 1.35\*\*\* |  |  |
| DF\_Large |  | 1.10 | 1.08 |
| ILR\_Large |  | 1.14 | 1.04 |
| Centrifuge |  | 1.23 | 1.11\*\* |
| Govt\_Building | 0.94 | 1.60 | 1.00 |
| Condition | 0.88\* | 0.93 | 1.01 |
| Water | 1.21\*\*\* | 0.91 | 1.01 |
| Toilet | 0.83\*\* | 0.76 | 1.11\*\*\* |
| StateAndra Pradesh | 6.92\*\*\* |  |  |
| StateArunachal Pradesh | 0.77 | 0.26 | 0.24\*\*\* |
| StateAssam | 6.91\*\*\* | 0.48 | 0.89 |
| StateBihar | 22.21\*\*\* | 8.80 | 1.45\*\* |
| StateChhattisgarh | 5.19\*\*\* | 0.80 | 0.43\*\*\* |
| StateGoa | 0.0000 | 0.62 | 0.40\*\* |
| StateHaryana | 7.53\*\*\* | 1.40 | 1.03 |
| StateHimachal Pradesh | 0.74 | 0.00 | 0.41\* |
| StateJharkhand | 10.44\*\*\* | 0.82 | 0.55\*\*\* |
| StateKarnataka | 5.39\*\*\* | 1.25 | 0.47\*\*\* |
| StateKerala | 29.85\*\*\* | 2.95 | 0.16\*\*\* |
| StateMadhya Pradesh | 11.61\*\*\* | 1.09 | 0.34\*\*\* |
| StateMaharashtra | 4.57\*\*\* |  | 0.00 |
| StateManipur | 0.71 | 1.31 | 0.16\*\*\* |
| StateMeghalaya | 8.05\*\*\* | 1.62 | 0.30\*\*\* |
| StateMizoram | 1.82 | 0.33 | 0.17\*\*\* |
| StateNagaland | 1.51 | 0.17 | 0.17\*\*\* |
| StateOdisha | 7.60\*\*\* | 0.73 | 0.98 |
| StatePuducherry | 102.50\*\*\* |  |  |
| StatePunjab | 5.34\*\*\* | 0.0000 | 0.18\*\* |
| StateRajasthan | 4.85\*\*\* |  |  |
| StateSikkim | 1.54 | 1.00 | 0.56 |
| StateTamil Nadu | 6.27\*\*\* |  |  |
| StateTelangana | 3.82\*\* | 2.60 | 1.51\*\* |
| StateTripura | 3.16 | 0.89 | 0.06\*\*\* |
| StateUttar Pradesh | 10.35\*\*\* | 1.17 | 0.66\*\*\* |
| StateUttrakhand | 3.14\*\* | 0.77 | 0.35\*\*\* |
| StateWest Bengal | 4.61\*\*\* | 0.26 | 2.41\*\*\* |
| ElectricityNo Electricity:Generator | 2.09\*\*\* | 1.86 | 1.00 |
| ElectricityNo Electricity:`24x7` | 1.27 | 0.62 | 0.87 |
| ElectricityNo Electricity:MO\_Total | 0.81\*\* | 0.96 | 1.09\*\* |
| ElectricityNo Electricity:LMO\_Total | 0.82 | 0.48 | 0.93 |
| ElectricityNo Electricity:Nurse\_Total | 1.02 | 0.91 | 1.04 |
| ElectricityNo Electricity:LHV\_Total | 1.63\*\*\* | 1.12 | 0.99 |
| ElectricityNo Electricity:ANM\_Total | 1.21\*\*\* | 0.92 | 1.07\*\* |
| ElectricityNo Electricity:Pharma\_Total | 0.88 | 0.93 | 1.13\* |
| ElectricityNo Electricity:MO\_Residing | 0.86 | 0.72 | 0.89 |
| ElectricityNo Electricity:Autoclave | 1.01 | 0.89 | 1.07 |
| ElectricityNo Electricity:RadiantWarmer | 1.24 |  |  |
| ElectricityNo Electricity:DF\_Large |  | 1.24 | 1.54\* |
| ElectricityNo Electricity:ILR\_Large |  | 0.74 | 0.79 |
| ElectricityNo Electricity:Centrifuge |  | 1.29 | 0.97 |
| Constant | 1.31 | 10.10 | 532.92\*\*\* |
|  | | | |
| Observations | 4,047 | 2,784 | 2,935 |
| Log Likelihood | -10,373.86 | -8,219.25 | -21,612.37 |
| theta |  |  | 1.87\*\*\* (0.05) |
| Akaike Inf. Crit. |  |  | 43,342.75 |
|  | | | |
| *Note:* | \*p<0.1; \*\*p<0.05; \*\*\*p<0.01 | | |
