# Supplementary material for: Reduced health services at under-electrified primary healthcare facilities: Evidence from India
Source: PLoS One. 2021 Jun 4;16(6):e0252705. doi: 10.1371/journal.pone.0252705 (PMC8177862; doi:10.1371/journal.pone.0252705)
Supplement: S1 Replication materials — (ZIP) [file pone.0252705.s002.zip › Replication material - PLOS ONE Review - Revised/Results/All_Models_Subsample_Reg_Irr.html]

**All Models - Subsample - Regular and Irregular Electricity**

|  | | | |
|  | *Dependent variable:* | | |
|  |  | | |
|  | Deliveries | IPD | OPD |
|  | *zero-inflated* | *zero-inflated* | *negative* |
|  | *count data* | *count data* | *binomial* |
|  | (1) | (2) | (3) |
|  | | | |
| ElectricityIrregular Electricity | 0.93 | 1.07 | 0.93 |
| Generator | 1.03 | 1.23\*\*\* | 1.25\*\*\* |
| Urban | 0.78\*\*\* | 0.82\*\* | 0.95 |
| Population10000 | 1.05\*\*\* | 1.02\*\*\* | 1.02\*\*\* |
| `24x7` | 1.47\*\*\* | 1.35\*\*\* | 1.09\*\* |
| Beds | 1.01\*\*\* | 1.06\*\*\* | 1.00 |
| MO\_Total | 1.05\*\* | 1.12\*\*\* | 1.10\*\*\* |
| LMO\_Total | 0.99 | 0.90 | 0.99 |
| Nurse\_Total | 1.01 | 1.07\*\* | 1.06\*\*\* |
| LHV\_Total | 1.07\*\*\* | 0.98 | 1.02 |
| ANM\_Total | 1.04\*\*\* | 1.03 | 1.02\*\* |
| Pharma\_Total | 0.96 | 1.02 | 1.06\* |
| MO\_Residing | 1.11\*\* | 1.32\*\*\* | 1.08\*\* |
| Autoclave | 1.07 | 1.20\*\* | 1.08\*\* |
| RadiantWarmer | 1.28\*\*\* |  |  |
| DF\_Large |  | 0.98 | 1.07 |
| ILR\_Large |  | 1.16 | 1.02 |
| Centrifuge |  | 1.19\*\* | 1.19\*\*\* |
| Govt\_Building | 0.99 | 1.09 | 1.08 |
| Condition | 0.97 | 0.91 | 0.98 |
| Water | 1.09\*\* | 0.94 | 1.06\*\* |
| Toilet | 0.75\*\*\* | 0.92 | 1.13\*\*\* |
| StateAndra Pradesh | 5.11\*\*\* |  |  |
| StateArunachal Pradesh | 0.81 | 0.28\*\*\* | 0.31\*\*\* |
| StateAssam | 6.10\*\*\* | 0.32\*\*\* | 0.93 |
| StateBihar | 22.88\*\*\* | 4.98\*\*\* | 1.93\*\*\* |
| StateChhattisgarh | 3.37\*\*\* | 0.72\* | 0.45\*\*\* |
| StateGoa | 4.87\*\*\* | 0.49\* | 0.71 |
| StateHaryana | 6.00\*\*\* | 0.97 | 1.06 |
| StateHimachal Pradesh | 1.95\* | 0.21\*\*\* | 0.68\*\* |
| StateJharkhand | 7.54\*\*\* | 0.81 | 0.53\*\*\* |
| StateKarnataka | 3.76\*\*\* | 0.95 | 0.52\*\*\* |
| StateKerala | 6.73\*\*\* | 2.65\*\*\* | 0.95 |
| StateMadhya Pradesh | 8.20\*\*\* | 0.90 | 0.42\*\*\* |
| StateMaharashtra | 3.44\*\*\* | 1.41 | 0.15\*\*\* |
| StateManipur | 1.77 | 1.06 | 0.20\*\*\* |
| StateMeghalaya | 2.54\*\*\* | 0.66\* | 0.51\*\*\* |
| StateMizoram | 1.54 | 0.48\*\*\* | 0.22\*\*\* |
| StateNagaland | 0.83 | 0.22\*\* | 0.19\*\*\* |
| StateOdisha | 5.06\*\*\* | 1.22 | 1.17 |
| StatePuducherry | 13.83\*\*\* |  |  |
| StatePunjab | 4.73\*\*\* | 0.0000 | 0.23\*\*\* |
| StateRajasthan | 3.83\*\*\* |  |  |
| StateSikkim | 1.29 | 0.55\*\* | 0.42\*\*\* |
| StateTamil Nadu | 4.29\*\*\* | 10.83\*\*\* | 4.08\*\*\* |
| StateTelangana | 3.11\*\*\* | 1.48\* | 1.36\*\* |
| StateTripura | 2.79\*\*\* | 1.18 | 0.50\*\*\* |
| StateUttar Pradesh | 8.09\*\*\* | 1.17 | 0.81\* |
| StateUttrakhand | 2.53\*\*\* | 0.61\*\* | 0.53\*\*\* |
| StateWest Bengal | 2.80\*\*\* | 0.58 | 2.63\*\*\* |
| ElectricityIrregular Electricity:Generator | 0.93 | 1.02 | 0.90\*\* |
| ElectricityIrregular Electricity:`24x7` | 1.04 | 0.98 | 1.00 |
| ElectricityIrregular Electricity:MO\_Total | 0.96 | 0.99 | 1.02 |
| ElectricityIrregular Electricity:LMO\_Total | 0.97 | 1.13 | 1.02 |
| ElectricityIrregular Electricity:Nurse\_Total | 1.07\*\*\* | 1.02 | 0.98 |
| ElectricityIrregular Electricity:LHV\_Total | 0.98 | 1.17\*\* | 1.03 |
| ElectricityIrregular Electricity:ANM\_Total | 0.98\* | 0.96\* | 1.01 |
| ElectricityIrregular Electricity:Pharma\_Total | 1.10\*\* | 1.02 | 1.03 |
| ElectricityIrregular Electricity:MO\_Residing | 1.22\*\*\* | 1.01 | 1.06 |
| ElectricityIrregular Electricity:Autoclave | 1.02 | 0.86 | 0.97 |
| ElectricityIrregular Electricity:RadiantWarmer | 1.12 |  |  |
| ElectricityIrregular Electricity:DF\_Large |  | 1.13 | 0.99 |
| ElectricityIrregular Electricity:ILR\_Large |  | 0.94 | 1.04 |
| ElectricityIrregular Electricity:Centrifuge |  | 1.06 | 0.97 |
| Constant | 1.70 | 11.26\*\*\* | 504.98\*\*\* |
|  | | | |
| Observations | 7,066 | 3,995 | 4,129 |
| Log Likelihood | -21,272.58 | -13,196.62 | -31,057.00 |
| theta |  |  | 2.04\*\*\* (0.04) |
| Akaike Inf. Crit. |  |  | 62,234.00 |
|  | | | |
| *Note:* | \*p<0.1; \*\*p<0.05; \*\*\*p<0.01 | | |
