# Supplementary material for: Reduced health services at under-electrified primary healthcare facilities: Evidence from India
Source: PLoS One. 2021 Jun 4;16(6):e0252705. doi: 10.1371/journal.pone.0252705 (PMC8177862; doi:10.1371/journal.pone.0252705)
Supplement: S1 Replication materials — (ZIP) [file pone.0252705.s002.zip › Replication material - PLOS ONE Review - Revised/Results/All_Models_Subsample_Reg_No.html]

**All Models - Subsample - Regular and No Electricity**

|  | | | |
|  | *Dependent variable:* | | |
|  |  | | |
|  | Deliveries | IPD | OPD |
|  | *zero-inflated* | *zero-inflated* | *negative* |
|  | *count data* | *count data* | *binomial* |
|  | (1) | (2) | (3) |
|  | | | |
| ElectricityNo Electricity | 0.31\*\*\* | 1.69\*\* | 0.64\*\*\* |
| Generator | 0.97 | 1.18\*\* | 1.26\*\*\* |
| Urban | 0.80\*\*\* | 0.71\*\*\* | 0.96 |
| Population10000 | 1.04\*\*\* | 1.03\*\*\* | 1.02\*\*\* |
| `24x7` | 1.53\*\*\* | 1.46\*\*\* | 1.09\* |
| Beds | 1.01\*\*\* | 1.05\*\*\* | 1.00 |
| MO\_Total | 1.02 | 1.09\*\*\* | 1.10\*\*\* |
| LMO\_Total | 0.98 | 0.92 | 0.99 |
| Nurse\_Total | 1.02 | 1.09\*\*\* | 1.08\*\*\* |
| LHV\_Total | 1.08\*\*\* | 0.99 | 1.02 |
| ANM\_Total | 1.05\*\*\* | 1.01 | 1.03\*\* |
| Pharma\_Total | 0.99 | 1.06 | 1.05 |
| MO\_Residing | 1.15\*\*\* | 1.40\*\*\* | 1.07 |
| Autoclave | 1.08 | 1.22\*\* | 1.07 |
| RadiantWarmer | 1.25\*\*\* |  |  |
| DF\_Large |  | 0.96 | 1.07 |
| ILR\_Large |  | 1.12 | 1.02 |
| Centrifuge |  | 1.22\*\*\* | 1.21\*\*\* |
| Govt\_Building | 0.96 | 0.99 | 0.98 |
| Condition | 0.99 | 1.09 | 1.04 |
| Water | 1.08 | 1.01 | 1.12\*\*\* |
| Toilet | 0.73\*\*\* | 0.91 | 1.13\*\*\* |
| StateAndra Pradesh | 4.12\*\*\* |  |  |
| StateArunachal Pradesh | 0.78 | 0.14\*\*\* | 0.32\*\*\* |
| StateAssam | 5.79\*\*\* | 0.10\*\*\* | 1.06 |
| StateBihar | 23.07\*\*\* | 3.53\*\*\* | 2.42\*\*\* |
| StateChhattisgarh | 2.74\*\*\* | 0.44\*\*\* | 0.54\*\*\* |
| StateGoa | 6.84\*\*\* | 0.34\*\* | 0.87 |
| StateHaryana | 5.29\*\*\* | 0.48\*\* | 1.06 |
| StateHimachal Pradesh | 1.68 | 0.13\*\*\* | 0.84 |
| StateJharkhand | 7.60\*\*\* | 0.55 | 0.80 |
| StateKarnataka | 2.75\*\* | 0.71 | 0.62\*\* |
| StateKerala | 6.43\*\*\* | 1.56 | 1.06 |
| StateMadhya Pradesh | 7.15\*\*\* | 0.56\* | 0.54\*\*\* |
| StateMaharashtra | 2.90\*\*\* | 0.85 | 0.09\*\*\* |
| StateManipur | 5.97\*\*\* | 0.09\*\*\* | 0.20\*\*\* |
| StateMeghalaya | 1.41 | 0.28\*\*\* | 0.65\* |
| StateMizoram | 1.30 | 0.31\*\*\* | 0.27\*\*\* |
| StateNagaland | 0.88 | 0.26 | 0.18\*\*\* |
| StateOdisha | 3.64\*\*\* | 1.05 | 1.59\*\* |
| StatePuducherry | 5.23\*\*\* |  |  |
| StatePunjab | 4.08\*\*\* | 0.0000 | 0.27\*\*\* |
| StateRajasthan | 3.22\*\*\* |  |  |
| StateSikkim | 1.10 | 0.30\*\*\* | 0.44\*\*\* |
| StateTamil Nadu | 3.53\*\*\* | 6.43\*\* | 4.70\*\*\* |
| StateTelangana | 2.77\*\* | 0.75 | 1.18 |
| StateTripura | 2.40\*\* | 0.72 | 0.61\* |
| StateUttar Pradesh | 4.57\*\*\* | 0.79 | 0.95 |
| StateUttrakhand | 2.24\* | 0.35\*\*\* | 0.72 |
| StateWest Bengal | 2.47\*\* | 0.40\* | 3.21\*\*\* |
| ElectricityNo Electricity:Generator | 2.08\*\*\* | 1.61 | 0.88 |
| ElectricityNo Electricity:`24x7` | 1.52\*\* | 0.57\*\*\* | 0.88 |
| ElectricityNo Electricity:MO\_Total | 0.78\*\*\* | 0.96 | 1.10\*\* |
| ElectricityNo Electricity:LMO\_Total | 0.65 | 0.54\* | 0.97 |
| ElectricityNo Electricity:Nurse\_Total | 1.11 | 0.95 | 0.96 |
| ElectricityNo Electricity:LHV\_Total | 1.55\*\*\* | 1.45\* | 0.97 |
| ElectricityNo Electricity:ANM\_Total | 1.23\*\*\* | 0.91 | 1.08\*\* |
| ElectricityNo Electricity:Pharma\_Total | 1.03 | 0.70\*\* | 1.32\*\*\* |
| ElectricityNo Electricity:MO\_Residing | 1.01 | 0.73 | 1.03 |
| ElectricityNo Electricity:Autoclave | 1.02 | 0.73 | 1.05 |
| ElectricityNo Electricity:RadiantWarmer | 1.50 |  |  |
| ElectricityNo Electricity:DF\_Large |  | 1.51 | 1.43 |
| ElectricityNo Electricity:ILR\_Large |  | 0.70 | 0.86 |
| ElectricityNo Electricity:Centrifuge |  | 1.32 | 0.88 |
| Constant | 2.06\* | 18.24\*\*\* | 405.23\*\*\* |
|  | | | |
| Observations | 4,497 | 2,301 | 2,500 |
| Log Likelihood | -13,011.24 | -7,319.26 | -18,928.26 |
| theta |  |  | 1.69\*\*\* (0.05) |
| Akaike Inf. Crit. |  |  | 37,976.51 |
|  | | | |
| *Note:* | \*p<0.1; \*\*p<0.05; \*\*\*p<0.01 | | |
