# Supplementary material for: Reduced health services at under-electrified primary healthcare facilities: Evidence from India
Source: PLoS One. 2021 Jun 4;16(6):e0252705. doi: 10.1371/journal.pone.0252705 (PMC8177862; doi:10.1371/journal.pone.0252705)
Supplement: S1 Replication materials — (ZIP) [file pone.0252705.s002.zip › Replication material - PLOS ONE Review - Revised/Results/All_Models_With combined data.html]

**With combined data**

|  | | | |
|  | *Dependent variable:* | | |
|  |  | | |
|  | Deliveries | IPD | OPD |
|  | *zero-inflated* | *zero-inflated* | *negative* |
|  | *count data* | *count data* | *binomial* |
|  | (1) | (2) | (3) |
|  | | | |
| ElectricityIrregular Electricity | 1.00 | 0.92 | 0.95 |
| ElectricityNo Electricity | 0.58\*\*\* | 1.23 | 0.69\*\*\* |
| Generator | 1.00 | 1.18\*\*\* | 1.14\*\*\* |
| Urban | 0.82\*\*\* | 0.80\*\*\* | 0.90\*\*\* |
| Population10000 | 1.06\*\*\* | 1.03\*\*\* | 1.03\*\*\* |
| `24x7` | 1.55\*\*\* | 1.38\*\*\* | 1.12\*\*\* |
| Beds | 1.02\*\*\* | 1.03\*\*\* | 1.01\*\*\* |
| MO | 0.83\*\* | 0.89 | 0.95 |
| LMO | 1.11 | 1.22\*\*\* | 1.05 |
| Nurse | 1.19\*\*\* | 1.18\*\*\* | 1.17\*\*\* |
| LHV | 1.30\*\*\* | 0.99 | 1.06 |
| ANM | 1.01 | 0.98 | 1.05 |
| Pharma | 0.83\*\* | 1.05 | 1.04 |
| MO\_Residing | 1.02 | 1.28\*\*\* | 1.04 |
| Autoclave | 1.10\* | 0.97 | 1.19\*\*\* |
| RadiantWarmer | 1.29\*\*\* |  |  |
| DF\_Large |  | 1.00 | 1.08\*\* |
| ILR\_Large |  | 1.16\*\* | 1.04 |
| Centrifuge |  | 1.25\*\*\* | 1.11\*\*\* |
| Govt\_Building | 0.90\*\* | 1.27\*\*\* | 0.97 |
| Condition | 0.91\*\*\* | 1.00 | 0.99 |
| Water | 1.09\*\*\* | 0.98 | 1.03 |
| Toilet | 1.00 | 0.92\* | 1.19\*\*\* |
| StateAndra Pradesh | 15.43\*\*\* | 1.24 | 2.90\*\*\* |
| StateArunachal Pradesh | 1.24 | 0.62 | 0.45\*\*\* |
| StateAssam | 4.86\*\*\* | 1.97\*\* | 1.27 |
| StateBihar | 15.73\*\*\* | 4.61\*\*\* | 1.78\*\* |
| StateChhattisgarh | 2.98\*\*\* | 0.59\* | 0.58\*\* |
| StateGoa | 2.38\*\* | 0.89 | 1.04 |
| StateHaryana | 5.15\*\*\* | 0.75 | 1.23 |
| StateHimachal Pradesh | 1.30 | 0.72 | 0.90 |
| StateJharkhand | 3.68\*\*\* | 0.87 | 1.00 |
| StateKarnataka | 3.58\*\*\* | 0.88 | 0.81 |
| StateKerala | 2.83\*\*\* | 1.45 | 1.80\*\* |
| StateMadhya Pradesh | 7.74\*\*\* | 1.04 | 0.51\*\*\* |
| StateMaharashtra | 4.06\*\*\* | 0.83 | 0.87 |
| StateManipur | 1.96\*\* | 1.29 | 0.40\*\*\* |
| StateMeghalaya | 2.31\*\*\* | 0.73 | 0.68 |
| StateMizoram | 1.54 | 0.57\* | 0.23\*\*\* |
| StateNagaland | 0.96 | 0.24\*\* | 0.27\*\*\* |
| StateOdisha | 3.40\*\*\* | 1.31 | 1.22 |
| StatePuducherry | 5.38\*\*\* | 0.96 | 3.45\*\*\* |
| StatePunjab | 4.61\*\*\* | 0.68 | 0.90 |
| StateRajasthan | 3.61\*\*\* | 0.80 | 0.67\* |
| StateSikkim | 1.17 | 0.60 | 0.65\* |
| StateTamil Nadu | 4.20\*\*\* | 1.41 | 3.10\*\*\* |
| StateTelangana | 3.76\*\*\* | 1.53 | 2.03\*\*\* |
| StateTripura | 2.53\*\*\* | 3.95\*\*\* | 1.00 |
| StateUttar Pradesh | 3.58\*\*\* | 0.83 | 1.00 |
| StateUttrakhand | 2.21\*\*\* | 0.55\* | 0.70 |
| StateWest Bengal | 2.23\*\*\* | 0.86 | 3.67\*\*\* |
| SurveyDLHS4 | 0.57\*\*\* | 1.08 | 1.02 |
| ElectricityIrregular Electricity:Generator | 1.07 | 1.02 | 1.06 |
| ElectricityNo Electricity:Generator | 1.35\*\* | 1.71\*\*\* | 1.09 |
| ElectricityIrregular Electricity:`24x7` | 0.89\*\* | 0.92 | 0.95 |
| ElectricityNo Electricity:`24x7` | 0.88 | 0.76\*\* | 1.05 |
| ElectricityIrregular Electricity:MO | 1.15 | 1.05 | 1.06 |
| ElectricityNo Electricity:MO | 1.08 | 0.74 | 1.12 |
| ElectricityIrregular Electricity:LMO | 0.91 | 1.00 | 0.97 |
| ElectricityNo Electricity:LMO | 0.43\*\*\* | 0.94 | 0.88 |
| ElectricityIrregular Electricity:Nurse | 0.94 | 0.90 | 0.87\*\*\* |
| ElectricityNo Electricity:Nurse | 1.60\*\*\* | 0.90 | 0.97 |
| ElectricityIrregular Electricity:LHV | 0.82\*\* | 1.08 | 0.94 |
| ElectricityNo Electricity:LHV | 0.74\*\* | 0.76\* | 1.06 |
| ElectricityIrregular Electricity:ANM | 1.12 | 1.06 | 1.02 |
| ElectricityNo Electricity:ANM | 1.51\*\* | 1.65\*\* | 0.93 |
| ElectricityIrregular Electricity:Pharma | 1.09 | 0.91 | 1.04 |
| ElectricityNo Electricity:Pharma | 1.27\* | 0.81 | 1.21\*\*\* |
| ElectricityIrregular Electricity:MO\_Residing | 1.26\*\*\* | 1.06 | 1.13\*\*\* |
| ElectricityNo Electricity:MO\_Residing | 1.03 | 0.74\*\* | 0.88\* |
| ElectricityIrregular Electricity:Autoclave | 0.91 | 1.06 | 0.96 |
| ElectricityNo Electricity:Autoclave | 1.02 | 0.73\*\* | 0.96 |
| ElectricityIrregular Electricity:RadiantWarmer | 1.19\*\*\* |  |  |
| ElectricityNo Electricity:RadiantWarmer | 1.40\* |  |  |
| ElectricityIrregular Electricity:DF\_Large |  | 1.07 | 0.97 |
| ElectricityNo Electricity:DF\_Large |  | 1.09 | 1.16 |
| ElectricityIrregular Electricity:ILR\_Large |  | 0.93 | 1.03 |
| ElectricityNo Electricity:ILR\_Large |  | 0.82 | 1.02 |
| ElectricityIrregular Electricity:Centrifuge |  | 1.10 | 1.00 |
| ElectricityNo Electricity:Centrifuge |  | 1.27 | 0.94 |
| Constant | 2.51\*\*\* | 13.09\*\*\* | 419.39\*\*\* |
|  | | | |
| Observations | 14,898 | 11,563 | 11,996 |
| Log Likelihood | -46,967.32 | -36,763.15 | -91,788.92 |
| theta |  |  | 1.45\*\*\* (0.02) |
| Akaike Inf. Crit. |  |  | 183,733.90 |
|  | | | |
| *Note:* | \*p<0.1; \*\*p<0.05; \*\*\*p<0.01 | | |
