# Supplementary material for: Reduced health services at under-electrified primary healthcare facilities: Evidence from India
Source: PLoS One. 2021 Jun 4;16(6):e0252705. doi: 10.1371/journal.pone.0252705 (PMC8177862; doi:10.1371/journal.pone.0252705)
Supplement: S1 Replication materials — (ZIP) [file pone.0252705.s002.zip › Replication material - PLOS ONE Review - Revised/Results/Deliveries_Sensitivity.html]

**Deliveries Model Sensitivity Analysis**

|  | | | | | |
|  | *Dependent variable:* | | | | |
|  |  | | | | |
|  | Deliveries | | | | |
|  | *zero-inflated* | | | | |
|  | *count data* | | | | |
|  | No Limit | Deliveries<300 | Deliveries<200 | Deliveries<100 | Deliveries<50 |
|  | (1) | (2) | (3) | (4) | (5) |
|  | | | | | |
| ElectricityIrregular Electricity | 0.97 | 0.96 | 0.95 | 0.91 | 1.00 |
| ElectricityNo Electricity | 0.36\*\*\* | 0.37\*\*\* | 0.33\*\*\* | 0.36\*\*\* | 0.39\*\*\* |
| Generator |  |  |  |  |  |
|  | | | | | |
| Observations | 7,805 | 7,768 | 7,701 | 7,524 | 7,265 |
| Log Likelihood | -22,415.63 | -22,104.58 | -21,635.27 | -20,339.18 | -18,412.75 |
|  | | | | | |
| *Note:* | \*p<0.1; \*\*p<0.05; \*\*\*p<0.01 | | | | |
