# Supplementary material for: Reduced health services at under-electrified primary healthcare facilities: Evidence from India
Source: PLoS One. 2021 Jun 4;16(6):e0252705. doi: 10.1371/journal.pone.0252705 (PMC8177862; doi:10.1371/journal.pone.0252705)
Supplement: S1 Replication materials — (ZIP) [file pone.0252705.s002.zip › Replication material - PLOS ONE Review - Revised/Results/IPD_Sensitivity.html]

**IPD Model Sensitivity Analysis**

|  | | | | | | | | | |
|  | *Dependent variable:* | | | | | | | | |
|  |  | | | | | | | | |
|  | IPD | | | | | | | | |
|  | *zero-inflated* | | | | | | | | |
|  | *count data* | | | | | | | | |
|  | No Limit | IPD<750 | IPD<500 | IPD<300 | IPD<250 | IPD<200 | IPD<150 | IPD<100 | IPD<50 |
|  | (1) | (2) | (3) | (4) | (5) | (6) | (7) | (8) | (9) |
|  | | | | | | | | | |
| ElectricityIrregular Electricity | 1.05 | 1.02 | 1.06 | 1.22 | 1.30\*\* | 1.27\* | 1.21 | 1.09 | 1.21 |
| ElectricityNo Electricity | 1.52\* | 1.49\* | 1.48\* | 1.47\* | 0.56\*\* | 0.56\*\* | 0.61\* | 0.79 | 0.86 |
| Generator |  |  |  |  |  |  |  |  |  |
|  | | | | | | | | | |
| Observations | 4,540 | 4,520 | 4,481 | 4,400 | 4,369 | 4,336 | 4,291 | 4,207 | 3,869 |
| Log Likelihood | -14,396.67 | -14,204.62 | -13,855.52 | -13,218.65 | -12,955.69 | -12,703.17 | -12,345.94 | -11,655.48 | -9,460.60 |
|  | | | | | | | | | |
| *Note:* | \*p<0.1; \*\*p<0.05; \*\*\*p<0.01 | | | | | | | | |
