# Supplementary material for: Reduced health services at under-electrified primary healthcare facilities: Evidence from India
Source: PLoS One. 2021 Jun 4;16(6):e0252705. doi: 10.1371/journal.pone.0252705 (PMC8177862; doi:10.1371/journal.pone.0252705)
Supplement: S1 Replication materials — (ZIP) [file pone.0252705.s002.zip › Replication material - PLOS ONE Review - Revised/Results/OPD_Sensitivity.html]

**OPD Model Sensitivity Analysis**

|  | | | | | |
|  | *Dependent variable:* | | | | |
|  |  | | | | |
|  | OPD | | | | |
|  | *negative* | | | | |
|  | *binomial* | | | | |
|  | No Limit | OPD<6000 | OPD<5000 | OPD<4000 | OPD<3000 |
|  | (1) | (2) | (3) | (4) | (5) |
|  | | | | | |
| ElectricityIrregular Electricity | 0.94 | 0.99 | 0.98 | 0.97 | 0.96 |
| ElectricityNo Electricity | 0.62\*\*\* | 0.70\*\*\* | 0.70\*\*\* | 0.70\*\*\* | 0.73\*\*\* |
| Generator |  |  |  |  |  |
|  | | | | | |
| Observations | 4,782 | 4,708 | 4,665 | 4,610 | 4,505 |
| Log Likelihood | -35,832.35 | -35,006.86 | -34,585.72 | -34,042.45 | -33,043.10 |
| theta | 1.87\*\*\* (0.04) | 1.93\*\*\* (0.04) | 1.93\*\*\* (0.04) | 1.94\*\*\* (0.04) | 1.96\*\*\* (0.04) |
| Akaike Inf. Crit. | 71,812.70 | 70,161.71 | 69,319.43 | 68,232.90 | 66,232.19 |
|  | | | | | |
| *Note:* | \*p<0.1; \*\*p<0.05; \*\*\*p<0.01 | | | | |
