# Supplementary figures and images for: Reduced health services at under-electrified primary healthcare facilities: Evidence from India
Source: PLoS One. 2021 Jun 4;16(6):e0252705. doi: 10.1371/journal.pone.0252705 (PMC8177862; doi:10.1371/journal.pone.0252705)

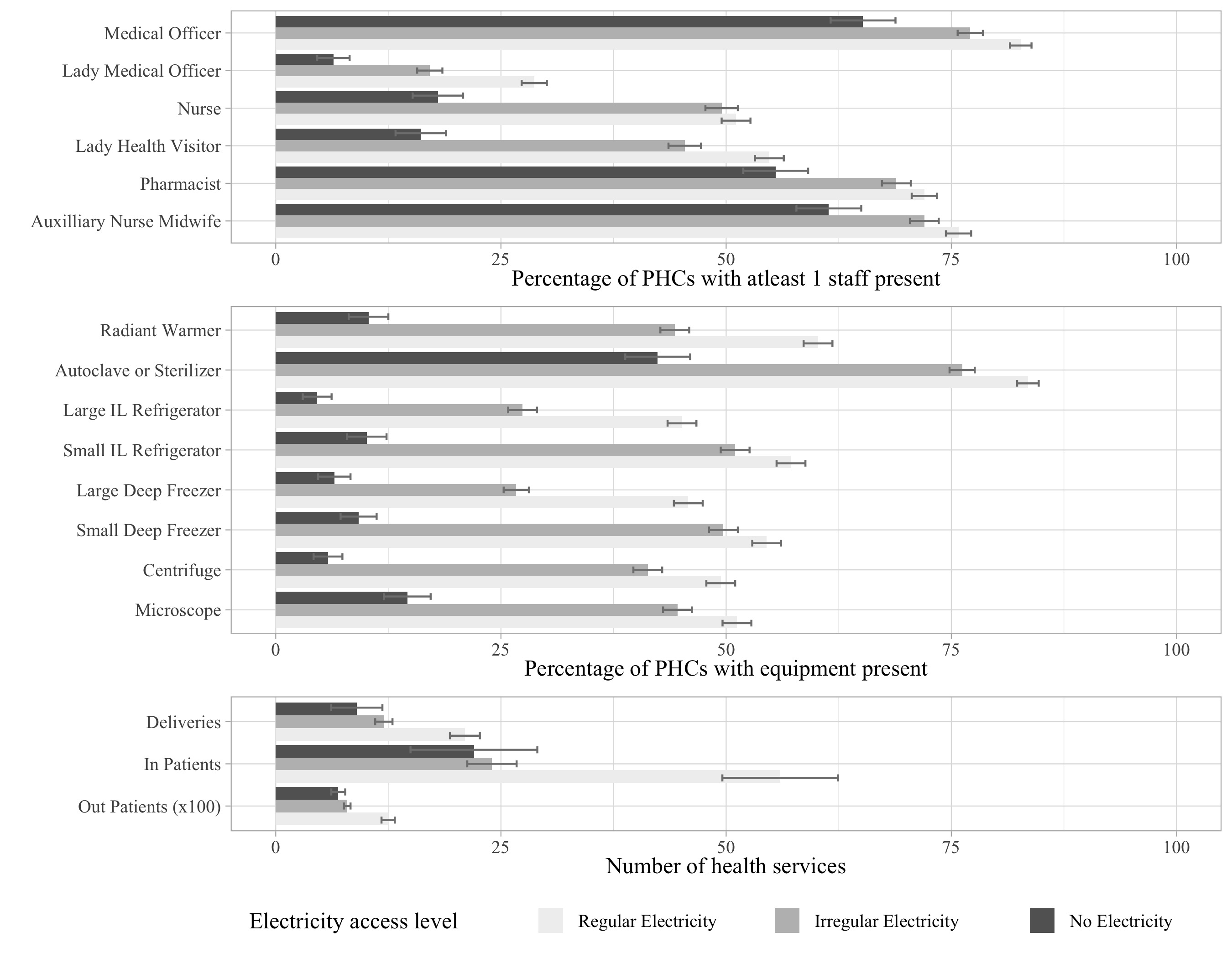

Supplement: S1 Replication materials — (ZIP) [file pone.0252705.s002.zip › Replication material - PLOS ONE Review - Revised/Results/descriptive_FINAL_separate.jpg]

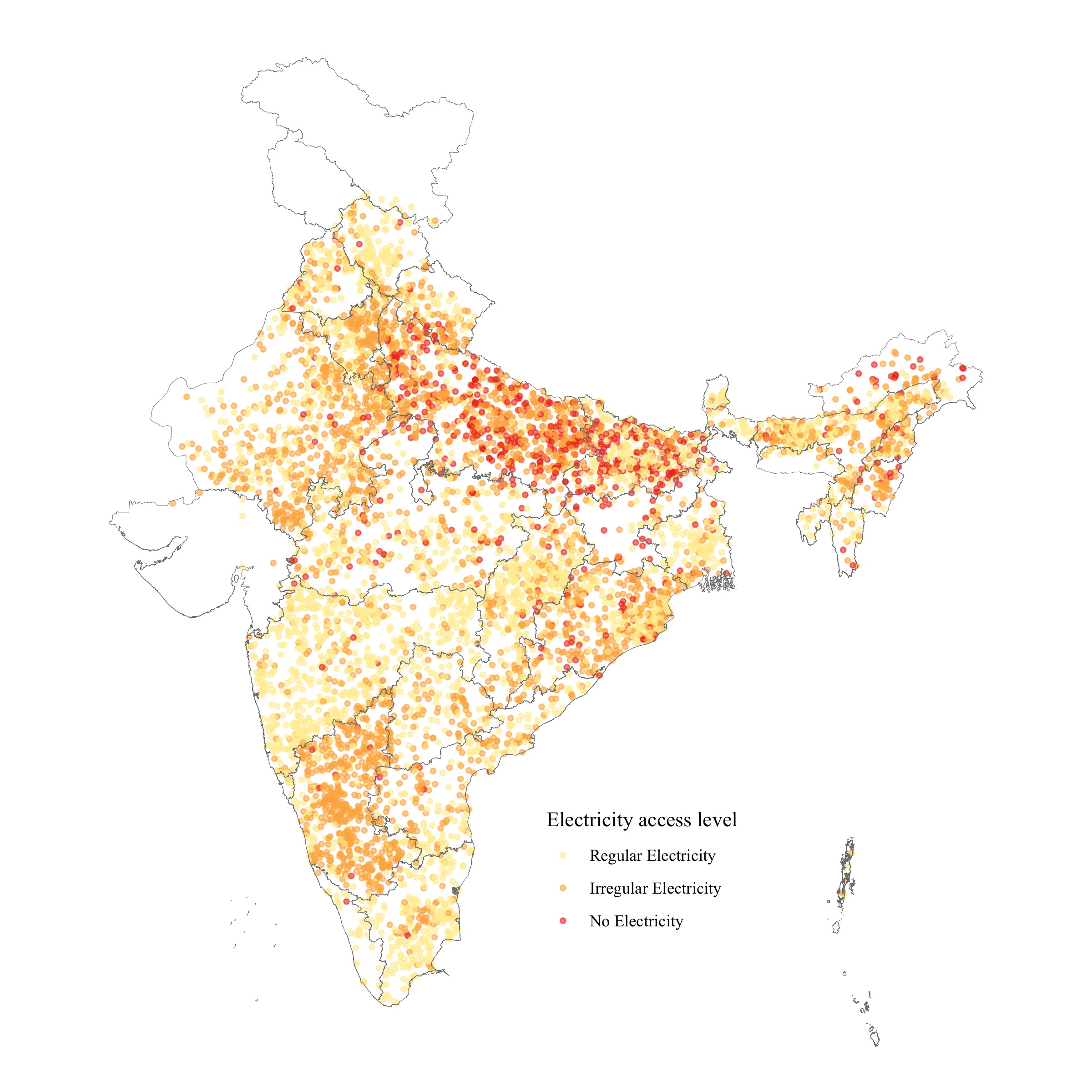

Supplement: S1 Replication materials — (ZIP) [file pone.0252705.s002.zip › Replication material - PLOS ONE Review - Revised/Results/phcmap.jpg]

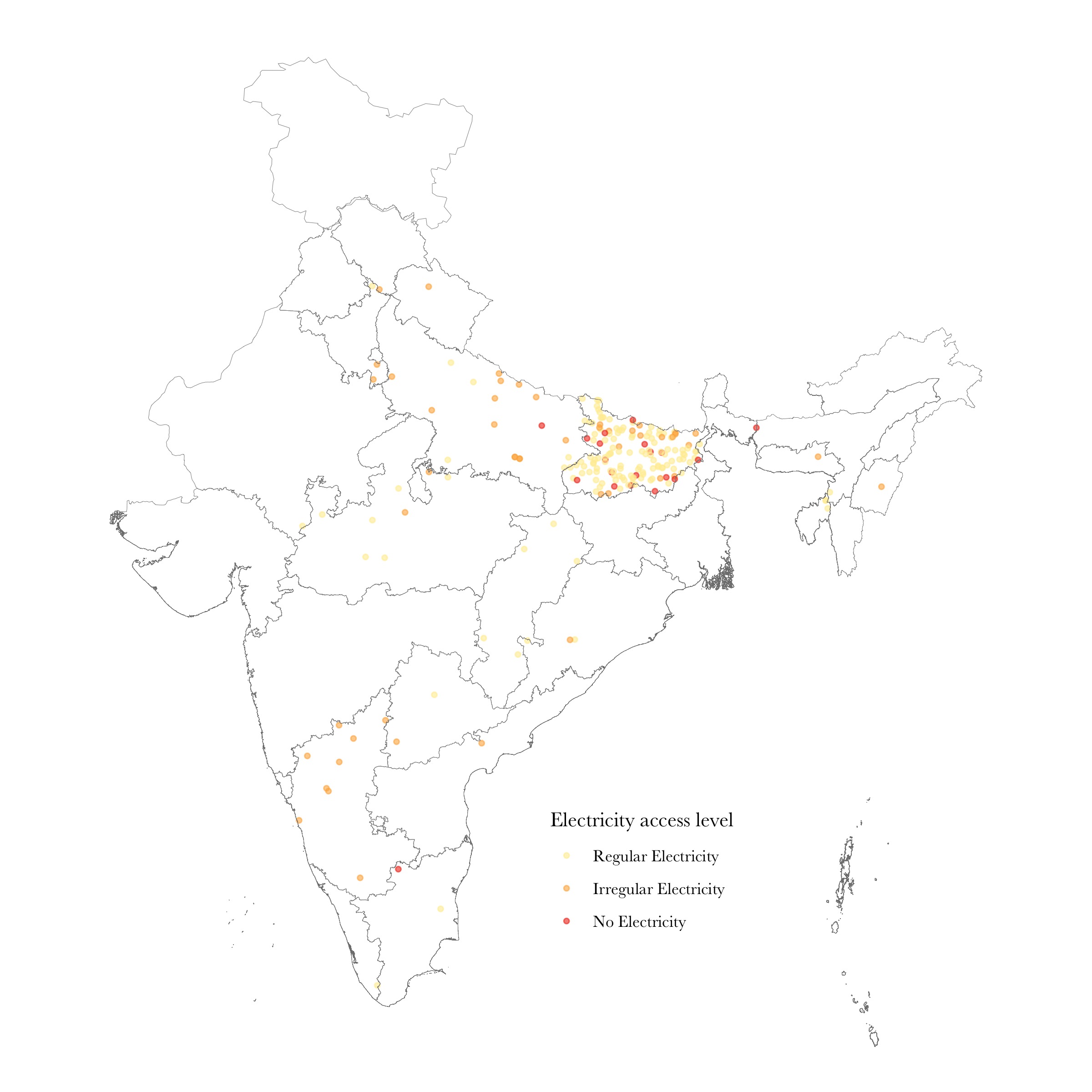

Supplement: S1 Replication materials — (ZIP) [file pone.0252705.s002.zip › Replication material - PLOS ONE Review - Revised/Results/phcmap_IPDabove150.jpg]
