# Supplementary material for: Reduced health services at under-electrified primary healthcare facilities: Evidence from India
Source: PLoS One. 2021 Jun 4;16(6):e0252705. doi: 10.1371/journal.pone.0252705 (PMC8177862; doi:10.1371/journal.pone.0252705)
Supplement: Attachment — Submitted filename: Response to Reviewers.docx [file pone.0252705.s003.docx]

Dear Dr. Zerriffi,

We thank you and the two reviewers for the valuable questions, comments, and suggestions. After careful consideration of each of the comments, we have conducted additional analyses, incorporated previous analyses that we had left out of the original manuscript, and provided additional details and discussion in light of the reviewers’ suggestions.

Below is a summary of the key changes we have made in the revised manuscript. Following this, we offer detailed responses to each of the reviewer’s comments.

- Multiple model specifications: We have now included two more parsimonious model specifications that help in demonstrating the robustness of our main estimates. We have also added additional justification on why OLS regression is not the right approach given the nature of our data. These changes together address one of the main concerns raised by reviewer #1.
- New analysis: We have included additional analysis such as sub-sample regressions (reported in the revised manuscript) and tests for multicollinearity (mentioned in the manuscript and made available in the code). These tests bring additional robustness and transparency to our analysis and they do not change any of our core findings. We thank reviewer #2 for these suggestions.
- Consolidated discussion of robustness tests: We have consolidated our discussion on the robustness checks both in the methods as well as the results sections. Specifically, we focus on testing robustness with respect to model specification, outliers, and sub-sample regressions.
- Clarification of limitations: In the original manuscript we had already acknowledged the absence of contextual data as a weakness in our analysis and that our findings do not establish clear causality. This was in line with reviewer #1’s concern that potential omitted variables limit causal implications that can be drawn from our analysis. Accordingly, in the revised manuscript we have expanded the discussion on these limitations and taken additional steps throughout the manuscript to avoid suggesting any causality in our findings.
- New discussion on irregular electricity: Based on reviewer #2’s observations about the policy relevance of irregular electricity category, we have now included new discussion in the results section about the statistically insignificant estimates for irregular electricity category.
- Additional supporting information: Some of the analyses that we had already conducted but left out of the original manuscript have now been included in the revised manuscript. This includes the outlier sensitivity analyses for the other two outcome variables (deliveries and out-patient) and the regressions with original five electricity-access categories.

We believe these changes have considerably strengthened the paper and bolstered the confidence in our findings, particularly around articulating the various robustness checks performed throughout the analysis.

**Detailed Responses to Comments from Reviewers:**

Please note, the reviewer comments are in black, and our responses are in blue.

**Responses to Reviewer #1**

Reviewer #1: How Energy Access Impacts Primary Healthcare
PONE-D-20-28579


Summary
-------

This paper reports results from cross-sectional analyses of data on primary health centers (PHCs) from one round of India's District Level Household and Facility Survey. Specifically, using zero-inflated negative binomial (ZINB) and negative binomial (NB) regressions, the authors explore the relationship between a set of PHC-level healthcare use and quality metrics in the month prior to the survey, and PHC-level electrification status. The authors conclude that lack of access to electricity is associated with (i) lower availability of essential medical equipment; (ii) lower availability of essential medical staff; and (iii) lower levels of healthcare service delivery (number of childbirths, in-patient visits, and outpatient visits) at the PHC level.


Main comments
-------------

1. Spatial variation in access to electricity is endogenously driven by a host of unobserved regional, institutional and socioeconomic characteristics. These unobserved characteristics can and do induce variation in the composition of households/beneficiaries, investments in complementary infrastructure (such as rural roads), and the presence or absence of other social welfare schemes. All of these can--and do--independently induce variation in the availability, quality, staffing and use of healthcare facilities. Beyond the inclusion of state fixed-effects, the authors have made no effort to account for this inherent endogeneity, which is surprising given the relative richness of their data. Extensions of the existing analyses using basic quasi-experimental approaches that take advantage of the metrics on PHS characteristics available (e.g., propensity score matching or other matching methods; two-step Heckman correction/endogenous treatment regression models) would greatly enhance the analytical rigor of the paper and allow the authors to make a more significant contribution to the literature on energy and healthcare delivery. Absent the use of these additional approaches, the simple cross-sectional regressions presented in the study (even those that control for a host of PHS-level characteristics) are insufficient to support the causal claims that the authors make throughout the paper (e.g., "how energy access impacts primary healthcare" in paper title; "effect on" medical equipment/medical staff/health service delivery in section 3.1/3.2/3.3).

Response: We thank the reviewer for raising these important concerns. We agree with the reviewer that cross-sectional nature of the available data imposes strong limitations on our ability to make causal claims about the relationship between electricity access and health service delivery. Within the bounds of these limitations, however, the rigorousness and transparency of our analysis along with the range of robustness checks we have conducted for the original draft and now in the course of this review process give us confidence in our core finding that there exists a negative association between electricity access and health service delivery that is deserving of policy attention and further research. After careful consideration of the each of the reviewer’s concerns and suggestions, we offer the following responses:

- We recognize that omitted variables are a limitation, and we already acknowledge that in section 2.4 that we are unable to control for many other community level factors that are known to influence healthcare delivery. Within this universe of omitted variables there may be some that may endogenously influence electricity access and healthcare delivery. We have now made this concern explicit in our discussion of the limitations on page 9.
- Despite the richness of the dataset with regard to PHC level characteristics, unfortunately the dataset does not contain information on external community level factors that we can test and control for potential endogeneity. We were unable to find any worthy instrumental variables in the available data that can be used in two-step instrumental variable regression models to treat endogeneity.
- While we did not present this in our manuscript, during our analysis for the original manuscript we did implement propensity score matching as well as propensity score weighted regression but found two serious limitations in this approach for our purposes. First, we found that 80 percent of the PHCs in the matched sample were selected from only three states (Uttar Pradesh, Orissa and Chhattisgarh) and many of the other states are not represented at all. Second, since the calculation of propensity score requires us to have a binary variable, we had to merge the irregular and regular electricity categories together as the “control” category in order to consider the no electricity category as the “treatment” category, thus causing us to loose important nuance in the analysis. Both of these constrains raised serious limitations for our analysis and therefore propensity score methods were abandoned.

Finally, from the outset we were fully aware of the limitations preventing us from making causal claims about the relationship. Throughout the text in the original manuscript, we have referred to our results in terms of associations and not in terms of causation. We do take cognizance of the title itself and some of the headings that the reviewer has pointed to, which convey an unintended causal tone. We have revised these specific instances to remove any causal connotations.

2. Various aspects of the data and empirical specification are unclear or seemingly arbitrary.
(I) why does the sample of PHCs fall by nearly 50% when looking at number of in-patient and outpatient visits (Table 1)?

Response: The sample size for in-patient and out-patient visits drops only because the data for these two variables is missing on the account of the question not being answered by the respondent. We thank the reviewer for raising this question. To address it, in Table 1 on page 7 we have now included a brief explanation for the drop in sample size.

(II) Why would an acute encephalitis outbreak that affected only one state (Bihar, p. 13) necessitate the removal of hundreds of PHCs from the analytical sample if the empirical specification also contains state fixed-effects (p. 6), which control for all unobserved state-specific shocks in a cross-sectional analysis? It is unclear why the authors feel that this adjustment to the sample (which reverse a statistically significant result) is justified while "any exclusions beyond this results in too many unjustified exclusions" (p. 14).

Response: We thank the reviewer for raising this concern. Our motivation for conducting the *sensitivity* analysis for the outcome variables was to investigate the unstable behavior of the estimate for PHCs without electricity in the In-Patient model. Our presentation of this analysis may have mistakenly come across as an effort to find an expected result. On the contrary, we have taken the effort to present a transparent analysis of the sensitivity of the main estimates to potential outliers. In the revised manuscript we have offered two reasons for why the exclusion of these PHCs maybe justified.

- First, since ZINB models contain large number of zeros and the data are highly skewed towards zero, these models can be highly sensitive to the presence of extreme outliers in the data. Indeed, these extreme outliers are defined as those values that exceed the 3^rd^ quartile by 3 times the IQR value, which in our data corresponds to PHCs with over 150 In-Patients (Page 15 and 16 in the manuscript). Based on this definition, any exclusion beyond this threshold would not be considered as extreme outlier and therefore their removal from the sample many not be justified. This is where our contention in the original draft that “*any exclusions beyond this results in too many unjustified exclusions*” was coming from.
- Second, the encephalitis outbreak was a *localized* (sub-state) event that occurred in some regions in Bihar and also in the adjacent state of Uttar Pradesh (this has been updated in the manuscript). These localized events cannot be adequately captured by the state level fixed effects, since they do not systematically affect all PHCs in a state. This discussion has been included in the revised manuscript in page 16.

In summary, while we do not advocate removing the PHCs from the sample to reverse a statistically significant result, we do believe we have provided a transparent explanation in the manuscript as to why the unstable result may be sensitive to outliers. In fact, in the main regression results presented in Table 3 as well as Table A1 in the appendix S1, we have also presented the results for the full model without any exclusions.

(III) What additional value do the ZINB and NB add relative to a conventional linear regression model? Without benchmark OLS estimates, seeing how the ZINB/NB estimates provide more precise results is difficult.

Response: We thank the reviewer for raising this point. As we have noted on page 7, all three of our outcome variables are count data which exhibit high over-dispersion. The distributions are highly skewed and non-normal. In such cases, OLS is known to produce biased and inefficient estimates. Use of NB models are recommended and the standard in such cases. We have included additional clarification and references to further stress this point on page 7. Furthermore, as noted before, the presence of large number of zeros in two of the variables make it necessary to use a zero-inflated model (ZINB) for these two variables. Since OLS is not appropriate in this context, these models have not been presented in the manuscript. However, we did run the OLS models for all three outcome variables, and the direction and significance of the focus variables do not change. These results are shown in Exhibits A and B in the supplementary information for reviewer #1.

(IV) Similarly, how do the inclusion of dozens of controls and interactions affect the coefficients on the main "focus variables" (FVs)? Without benchmark results included from a parsimonious specification (with only the FVs and the state fixed-effects), the stability (or lack thereof) of the estimates of interest is unclear.

Response: We thank the reviewer for raising this point. We had tested a range of intermediate models that were not included in the original manuscript. Based on this suggestion, we have updated table 3, which now includes the estimates from a parsimonious model (with only FVs and the state fixed-effects) as well as an intermediate model (with FVs, control variables and state fixed effects, but without interactions). These model specifications are mentioned on page 9. We have also added additional text to reflect that the direction and significance of estimates for No Electricity in the Deliveries and Out-Patient models are robust to model specification (page 14), whereas the estimate for No-electricity in the In-Patient model (page 15) as well as the estimates for Irregular Electricity in all three models (page 18) are not robust to model specification.

We further note that since many of the interactions included in the full model specification are statistically significant, the focal variable coefficients in the parsimonious models will be biased. Therefore, even though the estimates from all three model specifications are broadly consistent, we focus on the fully specified model for interpretation. This discussion is included on page 16 in the revised manuscript.

(V) Why do the authors use state fixed-effects instead of district fixed-effects? Presumably the District Level Household and Facility Survey contains information on the district where each PHC is located, and district fixed-effects would control for unobserved spatial characteristics more rigorously.

Response: The information on districts is available in the survey, and we did test district fixed effects model. Since there are over 500 districts in the sample, the district dummies consume significant degrees of freedom and *ex ante* it is expected that statistical significance of the coefficient estimates would suffer. When we ran models with district fixed effects, those models did not converge for two out of the three outcome variables and none of the variables appeared as significant in these models. While we are not able to include district fixed effects, we believe that inclusion of state fixed effects still enables us to capture important time-invariant heterogeneities across the states, because state governments in India have a substantial say in how their public health system functions. Thus, we would expect the health facilities in each state to share certain structural and functional similarities that vary from state to state, therefore justifying the state fixed effects. We therefore retain the model with state fixed effects. This is discussed on page 7 in the manuscript.


Additional comments
-------------------

3. "did not identify a single study in which linking energy access and health outcomes was the primary objective” - This needs to be clarified to indicate that it refers to access to electricity, specifically. SDG7 defines energy access in terms of access to electricity as well as access to clean cooking, and there is a broad literature connecting access to cleaner cooking solutions with health outcomes (see Jeuland & Pattanayak, 2012).

Response: We thank the reviewer for pointing out this clarification. A brief discussion on the linkages between energy access and health outcomes at the household level was in fact included in an earlier draft. We have included a footnote with citations on page 3 clarifying this distinction.

4. "The analysis presented in this paper provides robust empirical evidence..." (p. 3) - Why is it "robust"? Robust to what? Aside from sensitivity analyses relating to only the in-patient visits indicator, the paper does not present robustness tests for the main results.

Response: We thank the reviewer for raising this point. Though we had conducted several robustness checks, the language in the manuscript did not fully reflect this. To answer the question, we have conducted three types of robustness checks for the primary focal variable estimates.

- Robustness with respect to model specification (reflected in Table 4 and text on page 14)
- Robustness with respect to outliers (reflected in Tables 4, A2, A3 and text on page 16)
- Robustness with respect to sub-sample regressions (reflected in Table 5 and text on page 19)

We have summarized these on page 9 in the revised manuscript.

5. Why does the paper rely only on one round of the DLHS? Mani et. al. (2019), who the paper cites, appear to use both DLHS-3 as well as DLHS-4. Would the use of multiple rounds allow for a PHC- or district-level panel/repeated cross-section to be created? This would enable more sophisticated analyses that control for trends in electrification over time, which the authors also note are a concern.

Response: We thank the reviewer for this valuable suggestion. We found that DLHS-4 uses an updated version of the survey instrument used in DLHS-3. After comparing the two survey instruments, we note that despite similarities in many of the variables used in our analysis, there are variations in the format of data collected. For instance, DLHS-4 contains data on the number of staff available at each facility, many of which have a statistically significant relationship with the outcome variables in our analysis. However, DLHS-3 only has a binary indicator of staff availability instead of the number of staff. In light of such differences in the survey instruments, pooling the two datasets would tradeoff essential richness of data we are able to use in our current analysis. We recognize that adding data from DLHS-3 would add more variance to our data sample in a way that could potentially increase the efficiency of our estimates, while also helping with time fixed effects. However, we note that this will still not help us account for potential endogeneity due to omitted variables, which is the main concern as pointed out by the reviewer (please see discussion in response to the reviewer’s comment #1 above).

6. If urban PHCs are completing the same survey instrument for the DLHS (i.e., if they are reporting their access to electricity using the same five responses used by the rural PHCs), it is not immediately clear why they need to be removed from the analytical sample. Inclusion of a rural/urban fixed-effect in the main specification should account for unobserved context-specific differences between the two types of PHCs.

Response: We thank the reviewer for this suggestion. We have included the urban/rural fixed effect in the model specification. Doing so added 264 PHCs back into the analysis and did not change any of our findings.

7. "...models using the original five categories do not change the substantive results." (p. 5) - These results should also be presented in the paper (in an appendix).

Response: We thank the reviewer for this suggestion. These results have been included in the appendix.

8. "However, we are not concerned about endogeneity due to reverse causality in our focus variable, as the quantity of health service delivered in the prior month at the health center does not cause changes in the level of electricity access." (p. 8) - As noted in main comment #1, endogeneity is not limited to concerns about reverse causality. Some third, unobserved variable can independently induce variation in both the FVs as well as electricity access (omitted variable bias), and the current analysis does not capture that.

Response: We agree with the reviewer. That is why we specifically identified that we are not concerned about “endogeneity due to reverse causality…”. Omitted variable bias is always a valid concern in analyses like presented in this manuscript. Please see our response to main comment #1 above.

9. Why is "generator" a focus variable in Table 3 but not indicated as such in Table 2?

Response: We thank the reviewer for pointing out this oversight – generator availability has been included in Table 2, as well as the preceding text as a focal variable.

10. The World Health Organization uses the term "female health worker" (see <https://www.who.int/news-room/commentaries/detail/female-health-workers-drive-global-health>), not "lady health worker," which is used throughout the paper.

Response: We thank the reviewer for pointing out this language. One of the categories of staff in the Indian public health system is an LHV, which stands for Lady Health Visitor. We mistakenly refer to this category as lady health worker – and have rectified this in our manuscript (pages 8 and S1 Appendix Table A1). Another category of staff is an ANM, or an Auxiliary Nurse/Midwife. This cadre is also referred to as female health worker, as pointed out by the reviewer. Since they are more commonly referred to as ANMs in the Indian health system, we have retained this category as is.

11. "The importance of these alternate power sources in improving the health services is reinforced from our findings" (p. 18) - Please clarify how the paper's current findings reinforce the need for additional solar deployment. The previous section highlights the importance of lighting at night ("most of the deliveries happen in the evenings or at night" - p. 18), and intermittent PHS-level solar panels do not address that concern.

Response: We thank the reviewer for raising this question. Two main findings from our analysis are, ceteris paribus, (a) PHCs without electricity deliver lower number of health services, and (b) PHCs without electricity but with a generator conduct twice as many deliveries. These two points underscore the importance of alternate sources of electricity especially in electricity deficit PHCs. We further discuss in section 4.3 some reasons why having diesel generator as an alternate source is inconvenient and unsustainable for the PHCs. Decentralized renewable energy sources, of which solar is one technology option, have been recently evaluated and shown to improve health service availability, especially at night times (Javadi et al. 2020). The very objective of appropriately designed solar technologies with battery storage is to plug the intermittencies in grid electricity supply.

**Reference:** Javadi D, Ssempebwa J, Isunju JB, Yevoo L, Amu A, Nabiwemba E, et al. Implementation research on sustainable electrification of rural primary care facilities in Ghana and Uganda. Health Policy and Planning. 2020;35 (Supplement_2):ii124–36.

12. Typos:
- "Medical equipment that is..." not "medical equipment are" (used twice on p. 10)
- "consistently lower" not "consistently lesser" (p. 11)

Response: We thank the reviewer for pointing out these typos. They have been rectified in the manuscript.

References
----------

Jeuland, M. A., & Pattanayak, S. K. (2012). Benefits and Costs of Improved Cookstoves: Assessing the Implications of Variability in Health, Forest and Climate Impacts. PLoS ONE, 7(2), e30338. <https://doi.org/10.1371/journal.pone.0030338>

Mani S, Patnaik S, Dholakia HH. State of Electricity Access for Primary Health Centres in India, - Insights from the District Level Household and Facility Survey (DLHS-3 and DLHS-4) [Internet]. New Delhi: CEEW; 2019 Feb [cited 2019 Oct 18]. Available from: <https://www.ceew.in/sites/default/files/CEEW-The-State-of-Electricity-Access-for- Primary_0.pdf>

**Responses to Reviewer #2**

Reviewer #2: The paper is based on the nationally representative District Level Household Survey (DLHS-4) data and talks about the importance of electricity access for healthcare services. It is generally well written and relevant to PLOSONE readership, especially in the decade of action for attainment of access to energy for community services. I recommend acceptance for publication after addressing the following points:

Response: We thank the reviewer for this feedback.

1. Since the DLHS data is around a decade old, authors should continuously refer to the latest data (say, Rural Health Statistics or any other such data on cold chain services or appliance inventory or electrification status), wherever possible. This will make it more relevant to the current context in India. For instance, somewhere in the Introduction section, authors should also highlight the current situation of PHC electrification through the latest rural health statistics (RHS) data. That will show how has the situation of unelectrified PHCs changed in the last 7-8 years. Currently, there are only around 5% of the PHCs that are unelectrified in the country, and since the process of household electrification is over, the government should try and electrify these facilities as soon as possible.

Response: We thank the reviewer for this suggestion. In response, we have now referred to statistics from the latest Rural Health Statistics (2018-19) at different points in the manuscript to increase relevance to the current context, for example on pages 4, 13 and 21 in the manuscript. We also note that between 2012 to 2019, the number of unelectrified PHCs reduced by 41% from 1919 to 795. These 795 unelectrified PHCs still serve at least 24 million rural individuals. Unfortunately, the Rural Health Statistics report does not provide any information on PHCs with irregular electricity supply and alternate sources, so we are unable to make a comparative statement with regard to these variables. We further note that the results from our analysis are relevant not only at the PHC level, but also to the next lower level of the health system – the Sub-Centers. As we write in section 4.1, the current policy push towards upgrading these Sub-Centers and pushing some the health services from the PHCs down to the Sub-Centers highlight the importance of electricity infrastructure necessary at this level. According to the latest Rural Health Statistics, over 25% of the Sub-Centers were still unelectrified, serving over 196 million rural individuals. This makes the findings from this PHC-level study highly relevant at the Sub-Center level as well.

2. In Table 1 (page 5), in order to put these numbers into perspective, authors should also highlight Indian Public Health Standards (IPHS) numbers that a PHC is supposed to serve in Table 1. For instance, what does a number of 9.65 deliveries mean? Further, how many PHCs are able to meet the IPHS guidelines for Deliveries, Inpatient and Out-Patient?

Response: We thank the reviewer for this suggestion. The IPHS guidelines classify PHCs that have a load of more than 20 deliveries in a month as Type-A PHCs and those with less than 20 deliveries a month as Type-B PHCs that are eligible for more resources^[1]^. Further, IPHS guidelines consider a load of 40 out-patients per doctor per day as being essential for a PHC. In the revised manuscript we have contextualized our discussion in section 3.3 with reference to these guidelines.

3. There will be massive scope for multicollinearity among the independent variables, as authors themselves highlight in their paper. For example, "much of the essential medical equipment are electricity dependent" and "percentage of PHCs with at least one staff member available was lower among 11 PHCs with a lower level of electricity access." While specifying the model on page 6, the authors should also talk explore and discuss the degree of multicollinearity between the independent variables (say, through VIF). This will help in both getting rid of redundant variables and making the effect sizes seem more robust.

Response: We thank the reviewer for raising this concern. We have computed the variable inflation factors (VIF) for the variables in all three models and did not find any evidence of multicollinearity. These results are shown in Exhibit 1 in the supplementary information document for the reviewer. None of the variables (with the exception of state fixed-effects) reach the VIF threshold of 5, at which point multicollinearity is considered a serious issue that deserves further investigation. We have included a brief discussion on this in the revised manuscript (page 7) and the analysis itself is available in the updated code submitted along with the manuscript.

4. When the authors say "Out Patient services decrease noticeably as well in PHCs with irregular electricity supply" on page 11, I am assuming it comes from the results. If indeed that is the case, please refer to the Table number here. Also, authors should be careful while making such statements. It is not clear if Out-patient services decrease with poor electricity supply. You would need a time-series data to use the word 'decrease'. This is cross-sectional data. At best, it can only be said that PHCs with irregular electricity supply can serve a lesser number of OPD patients.

Response: We thank the reviewer for this suggestion. The particular line that the reviewer is referring to has been removed for conciseness. However, this suggestion has been taken into account for other similar language appearing in the manuscript. For example, see the language in page 14 of the revised manuscript, where we write “*We find that PHCs without electricity access are associated with 64 percent lower number of deliveries and 38 percent lower number of out-patients treated as compared to PHCs with regular electricity access*”

5. Visualization comment - In Figure 4 (page 12), authors should use a different colour for PHCs with regular electricity supply (maybe lighter grey).

Response: We thank the reviewer for this suggestion. The color has been modified slightly.

6. In page 14, authors mention “We conducted similar analyses for the other two outcome variables (deliveries and out-patients) and found that these models are not as sensitive to the outliers.” Just like Table 4, even these results on sensitivity analysis for deliveries and outpatients should be presented in the appendix.

Response: We thank the reviewer for this suggestion. These two tables have now been included in the appendix.

7. Authors should also include the result on the interaction of electricity supply from the grid with the availability of generator in Table 3. For example, an irregularly electrified PHC with a generator is better than another irregularly electrified PHC without a generator. In remote regions where it is challenging to extend electricity supply through the grid (or where grid fluctuations are higher), this result will be important to make a case for electricity backups.

Response: We thank the reviewer for the suggestion of including the interaction with generator in table 3. The reviewer has also highlighted this point in comment #10 and we agree this suggestion. In response, we have updated table 3 to include these interactions. We agree with the reviewer that the case for alternate sources of electricity is higher especially in health facilities without grid electricity. We had made the same observation on page 18 of the manuscript, where we write – “while having a generator does not seem to have a significant impact on the number of deliveries conducted overall, when there is no electricity having a generator was associated with doubling in the number of deliveries. This highlights the criticality of non-grid power sources in rural health facilities with deficient access to grid power.”

8. In Table 3 (page 15), there is no significant difference between PHCs with irregular electricity and regular electricity supply. What policy message can we derive out of this for the current context? Are you saying that even if you provide irregular electricity supply, it is not going to significantly hamper the service delivery? Some discussion on this would be useful. I think in order to make this analysis even more policy-relevant, authors should perform following three separate regressions apart from the one presented in Table 3 (even if they choose to show these results in the Appendix). This will also help in doing the robustness checks for their results:
a. A subsample regression analysis only between irregularly electrified and regularly electrified PHCs to see if you can see the difference now. If there is a significant difference, then please try to highlight why this difference got lost in the bigger model where you had all three categories together (in Table 3).
b. Again, a sub-sample regression between irregularly and not electrified PHCs to see how service deliveries differ between these two categories.

Response: We thank the reviewer for these important observations and suggestions. It was indeed a surprising finding for us not to find any statistically significant difference between the PHCs with regular and irregular electricity supply. The sub-sample regressions suggested by the reviewer were helpful in further testing and confirming this finding. PHCs without electricity access are associated with lower number of services compared individually to regularly as well as irregularly electrified PHCs. However, contrary to our (and reviewer #2’s) expectation, we do not find any statistically significant difference in the outcomes between regular and irregularly electrified PHCs. We suggest this may be because of the reliability in coding the irregular electricity categories. For example, in two PHCs experiencing the same quality of electricity supply, one respondent may answer “occasional power cut” whereas the other may answer “regular power cut”, depending on their relative contexts. This might introduce subjectivity in how the irregular electricity variable is coded, potentially masking the significance of certain types of irregularity. Even when we expand the analysis using the original five categories, the only significant result is with regard to the number of out-patients at PHCs with regular power cuts, but overall, we do not find significant relationships with regard to the irregularly electrified PHCs. We believe more accurate quantification of the reliability of electricity can provide better evidence on the relationship between reliability of supply and health services delivered. We have added 2 additional paragraphs on page 18 and 19 summarizing these findings, which we believe improves the transparency of the findings.

Despite this shortcoming in the data, we believe we make important contributions both in terms of policy relevance and methodology. The fundamental relationships we find around lack of electricity access and health services delivered are relevant not only for the remaining unelectrified PHCs that serve over 24 million people in India, but also to the very high proportion of yet unelectrified sub-centers that serve over 196 million people. These findings are also relevant to other parts of the developing world where unreliable electricity at public health facilities still affects hundreds of millions of poor and underserved communities. We discuss this in section 4.1 in the manuscript.

c. Since the problem of no electricity access for PHCs is largely going to be done away with soon, club PHCs with irregular electricity supply and no electricity supply (assuming that PHCs with irregular power supply are equally worse).
These regressions might help in coming up with certain important policy recommendations.

Response: We thank the reviewer for this suggestion. When we merge the no electricity category together under the “irregular electricity” category, we did find that PHCs with “irregular electricity” were associated with 16 percent lower number of deliveries and outpatients compared to PHCs with regular electricity. These results are shown in Exhibit 2 in the supplementary information for the reviewer. While these results could be relevant for policy consideration to focus on irregularly electrified PHCs, we believe this may in fact be somewhat misleading because we do find a difference between un-electrified and irregularly electrified PHCs in our analysis. Merging these two categories would in turn mask the observed heterogeneity in the results. In view of this consideration and the discussion in response to comment #8 above (regular vs. irregular electricity PHCs), we believe it is best to leave the irregular and no-electricity categories separate.

9. In page 15, I think there is something wrong with the sentence “Absent reliable public health
facilities many women resort to delivering at private facilities that are often farther away and
more expensive, further increasing their burden.”

Response: We thank the reviewer for pointing this out - we have modified this sentence to read better.

10. In page 16, authors write “Second – while having a generator does not seem to have a significant impact on the number of deliveries conducted overall when there is no electricity having a generator was associated with doubling in the number of deliveries. This highlights the criticality of non-grid power sources in rural health facilities with deficient access to grid power.” I believe these results on access to electricity through generators should also be shown in Table 3, as this is equally relevant.

Response: As noted in response to comment #7, we have now included the interaction estimates in table 3.

11. About page 18, section 4.3 – Decentralized Renewable Energy (DRE) may not be a panacea and may also have its own challenges. In many places, they may even not have worked out well. If the DRE systems fail, it will have substantial cost implications. Some discussion on that aspect would also be useful to make a more balanced point around DRE. How to make DRE a more workable solution?

Response: We thank the reviewer for raising this point. We agree that DRE based solutions do have their own challenges around implementation and sustainability. On page 24, we have added a new reference to a very recently published paper (Javadi et al. 2020) which discusses the necessity for other health system and implementation factors to be aligned in order for these solutions to be sustainable.

**Reference:** Javadi D, Ssempebwa J, Isunju JB, Yevoo L, Amu A, Nabiwemba E, et al. Implementation research on sustainable electrification of rural primary care facilities in Ghana and Uganda. Health Policy and Planning. 2020;35 (Supplement_2):ii124–36.

1. These are specified in pages 1 and 2 of the executive summary in the IPHS guidelines for PHCs, available here - <https://nhm.gov.in/images/pdf/guidelines/iphs/iphs-revised-guidlines-2012/primay-health-centres.pdf>
