# Supplementary material for: Reduced health services at under-electrified primary healthcare facilities: Evidence from India
Source: PLoS One. 2021 Jun 4;16(6):e0252705. doi: 10.1371/journal.pone.0252705 (PMC8177862; doi:10.1371/journal.pone.0252705)
Supplement: Attachment — Submitted filename: Response to Reviewers.docx [file pone.0252705.s004.docx]

Dear Dr. Zerriffi,

We take this opportunity to thank you and the two reviewers again for their positive feedback and further suggestions. We have implemented and reported both additional analysis that reviewer #1 suggested, regarding district fixed effects (point 2) and pooling data from DLHS-3 (point 3). As we have detailed in our responses below, you will see these additions reflected in new sections 3.4.4 and 3.4.5 (pages 21-23) in the revised manuscript. We find that our main results are robust to both these additional tests. Therefore, while the additional analyses do not change the overall conclusions of the study, they certainly increase our confidence in the results.

Both rounds of the review process have been helpful in strengthening the findings and we appreciate the constructive suggestions from the reviewers. We believe we have responded sincerely to all outstanding concerns flagged by the reviewers.

**Detailed Responses to Comments from Reviewers:**

Please note, the reviewer comments are in black, and our responses are in blue.

**Responses to Reviewer #1**

Reviewer #1: This is my second review of the manuscript. I appreciate the authors’ adjustments to the text of the paper to more appropriately characterize their findings as non-causal. However, a number of my methodological concerns, outlined below, have not been addressed.

1. The justification for the use of zero-inflated negative binomial (ZINB) models remains unclear. The paper notes that there is over-dispersion because “the variance is much greater than the mean” (p. 6). But over-dispersion of count data involves comparing conditional variances to conditional means (not unconditional moments, as shown in Table 1). Is this the case?

Response: We thank the reviewer for raising this point. Yes, it is the case that conditional variances are much greater than conditional means. Table 1, as well as the corresponding text on page 6/7 have been updated with the conditional values.

2. The authors’ insistence on ZINB models also limits their ability to deploy district fixed-effects (FEs); as noted in the reviewer response document, ZINB models with district FEs fail to converge in their case. The use of these FEs is essential for rigorously accounting for unobserved spatial heterogeneity (including the concerns articulated around the localized outbreaks of acute encephalitis). The authors should consider the following Stata commands: reghdfe (for linear models with high-dimensional fixed effects, available at http://scorreia.com/software/reghdfe/) and ppmlhdfe (the corresponding command for Poisson models, available at http://scorreia.com/software/ppmlhdfe/). Both use efficient algorithms to absorb rather than estimate high-dimensional FEs and, in so doing, preserve degrees of freedom. At a minimum, report results from the linear model with the district FEs included. There is no need to report the coefficient for each estimated FE (indeed, neither reghdfe nor ppmlhdfe generate report these coefficients by default).

Response: We thank the reviewer for these suggestions. As articulated in the manuscript as well as in our previous response to the reviewers, our use of ZINB models as well as state fixed effects is driven by methodological considerations and data limitations. We do agree with the reviewer that district fixed effects could help account for some (although not all) of the unobserved spatial heterogeneity. To compare the state and district fixed effect models, we used OLS models instead of the ZINB models and retained the NB model for out-patient outcome variable since that one did converge. We have presented this analysis in section 3.4.4 and table 6 on page 20 and 21 in the revised manuscript. We find that the estimates are broadly robust to the type of fixed effect specified, and we do not observe any substantial changes to our overall findings. We also used a high-dimensional fixed effect linear model *felm* in R (<https://cran.r-project.org/web/packages/lfe/lfe.pdf>, available in the code), which produced very similar results as the normal linear model with fixed effects.

3. The authors’ reluctance to use the DLHS–3 in combination with DLHS–4 to generate a PHC-level panel is puzzling. The differences in how data are collected across the two waves do not rule out the possibility of pooling them. For instance, if DLHS–4 contains the total number and type of staff at the PHC while DLHS–3 only includes a binary variable for whether a particular type of staff member is present, a corresponding binary variable can be created in DHLS–4. The resulting panel admittedly contains less when it comes to that particular control variable but considerably richer as it enables controls for time trends and, perhaps most importantly, the use of PHC FEs if the same PHC is tracked in both survey rounds and there is temporal variation in access to electricity, i.e., access changes in some PHCs. Indeed, PHC FEs will fully account for the omitted variable bias that is almost certainly present in these analyses due to time-invariant characteristics, and the authors’ claim in the response that “this will still not help us account for potential endogeneity due to omitted variables” is incorrect. The tradeoffs between the panel and cross-sectional analyses can be articulated in the text; the authors don't need to pick only one approach.

Response: We thank the reviewer for imploring us to include data from DLHS-3. We agree that a panel dataset with PHC-level fixed effect would’ve allowed for greater control of the omitted variable bias. Unfortunately, the two surveys were based on different sampling units and therefore covered different PHCs. Still, we agree that pooling the data from both rounds would allow us to include a time fixed effect. We have presented this analysis in section 3.4.5 and table 7 on page 21 and 22 of the revised manuscript. Consistent with the other results, we find that the deliveries and out-patient coefficients are robust to the dataset specification and the inclusion of the time fixed effect. This analysis certainly strengthens our confidence in the overall findings, and we thank the reviewer again for encouraging us to include it.

4. My note in my original review that the concept of endogeneity is not limited to reverse causality concerns was intended to specifically highlight that the authors cannot rule out reverse causality. Consider, for instance, the following scenario: regional variation in population growth leads to an increase in PHC utilization (e.g., more deliveries) in some PHCs and not others. The government observes these trends and, in order to respond to this demand, makes investments in infrastructure at these PHCs, including electricity, i.e., utilization --> electricity.

Response: We thank the reviewer for flagging this concern again. We do agree with the reviewer that IF the probability of reverse causality were high, it may jeopardize the core findings in our paper. After giving this concern much consideration, we believe that while we cannot fully rule out a scenario where higher utilization may lead to electricity access (and hence our subdued claims regarding causality), we believe that the likelihood of such a scenario is fairly low whereas the reverse argument, that electricity access may trigger higher healthcare demand, is far more plausible. We offer the following explanation.

The electricity variable in the DLHS dataset measures availability of grid electricity and the quality (reliability) of the available electricity. Historically, rural electrification policies prioritized villages with higher populations or villages with higher agricultural activity (for energizing pump sets). More recent electrification efforts all aimed for 100% village electrification, perhaps prioritizing villages closer to existing grid lines and then extending to villages farther away. PHCs usually (in most cases) got electrified as part of this village electrification process, but it is also not uncommon for the village to be electrified but the PHC is not. To our knowledge, there haven’t been any targeted policy efforts that have systematically prioritized electrification of PHCs based on healthcare demands. There may be some exceptions and one-off cases such as the scenario the reviewer has mentioned, but that is unlikely to be widespread. PHCs also have some discretionary funds that they can use as they wish, but these are almost always used to procure emergency medicine supplies, equipment or minor repairs, and in rare cases generators or fuels. These funds are insufficient for extending power lines and getting electrified. In summary, it may be true that villages with higher population (which could relate to a greater number of people treated at the PHC) could have influenced better electricity availability. We do control for this population variable in all the models. It is far less likely that a trend in increased healthcare demand influenced an improvement in electricity availability. We believe the reverse - that improved electricity availability (together with other factors) could catalyze improved healthcare demand, is far more likely. The essence of this discussion is reflected in the additional text in section 2.4 on page 10.

**Responses to Reviewer #2**

Reviewer #2: The authors have done a thorough job in revising the manuscript to address the comments made in the previous review round and have provided a clear and complete documentation of the revisions made. The revised manuscript makes the important supplementary information/analyses much more transparent, which adds much value to this manuscript. I have no further major revisions to suggest and recommend a couple of minor revisions as I outline below. I congratulate them for their good job.

Response: We thank the reviewer for this positive feedback.

1. In page 4-5, when authors say “To put this in context, as of 2019 there were still 795 PHCs without electricity supply. These PHCs without electricity supply cumulatively served at least 24 million rural individuals.”, it would be important to mention as to what does this mean in terms of percentages. For instance, 24 million rural individuals represent what proportion of India’s overall population, and similar 795 represent what proportion of unelectrified PHCs. This will help in indentifying the significance of the issue.

Response: We thank the reviewer for this suggestion. The percentage value has been included in the revised manuscript.

2. In response to my first comment from the first round, authors respond “We also note that between 2012 to 2019, the number of unelectrified PHCs reduced by 41% from 1919 to 795.” It would be important to highlight what does this progress mean in terms of percentages. Depending on the data availability, I would suggest them to add a time series line graph (somewhere in the annexure) that shows the progress in PHC electrification in India in the past 12-13 years (decline in percentage of unelectrified PHCs on Y-axis and year on the X-axis). They will use DLHS-3 proportion for the year 2007-08 and DLHS-4 proportion for 2012-13. For the remaining years, they can refer to yearly rural health statistics from MoHFW’s website.

Response: We thank the reviewer for this suggestion. The figure has been included in the supplementary information (S1 Appendix Figure A1) and mentioned in the revised manuscript on page 5.
